# Thiol-ene "Click" Synthesis and Pharmacological Evaluation of *C*-Glycoside sp^2^-Iminosugar Glycolipids

**DOI:** 10.3390/molecules24162882

**Published:** 2019-08-08

**Authors:** Elena M. Sánchez-Fernández, M. Isabel García-Moreno, Raquel García-Hernández, José M. Padrón, José M. García Fernández, Francisco Gamarro, Carmen Ortiz Mellet

**Affiliations:** 1 Department of Organic Chemistry, Faculty of Chemistry, University of Seville, C/Profesor García González 1, 41012 Seville, Spain; 2 Instituto de Parasitología y Biomedicina “López-Neyra”, IPBLN-CSIC, Parque Tecnológico de Ciencias de la Salud, 18016 Granada, Spain; 3 BioLab, Instituto Universitario de Bio-Orgánica Antonio González (IUBO AG), Centro de Investigaciones Biomédicas de Canarias (CIBCAN), Universidad de La Laguna, 38206 La Laguna, Spain; 4 Instituto de Investigaciones Químicas (IIQ), CSIC - University of Sevilla, Avda. Américo Vespucio 49, 41092 Sevilla, Spain

**Keywords:** sp^2^-Iminosugars, *C*-glycosides, glycolipids, glycomimetics, glycosidase inhibitors, Leishmaniasis, cancer

## Abstract

The unique stereoelectronic properties of sp^2^-iminosugars enable their participation in glycosylation reactions, thereby behaving as true carbohydrate chemical mimics. Among sp^2^-iminosugar conjugates, the sp^2^-iminosugar glycolipids (sp^2^-IGLs) have shown a variety of interesting pharmacological properties ranging from glycosidase inhibition to antiproliferative, antiparasitic, and anti-inflammatory activities. Developing strategies compatible with molecular diversity-oriented strategies for structure–activity relationship studies was therefore highly wanted. Here we show that a reaction sequence consisting in stereoselective *C*-allylation followed by thiol-ene “click” coupling provides a very convenient access to α-*C*-glycoside sp^2^-IGLs. Both the glycone moiety and the aglycone tail can be modified by using sp^2^-iminosugar precursors with different configurational profiles (d-*gluco* or d-*galacto* in this work) and varied thiols, as well as by oxidation of the sulfide adducts (to the corresponding sulfones in this work). A series of derivatives was prepared in this manner and their glycosidase inhibitory, antiproliferative and antileishmanial activities were evaluated in different settings. The results confirm that the inhibition of glycosidases, particularly α-glucosidase, and the antitumor/leishmanicidal activities are unrelated. The data are also consistent with the two later activities arising from the ability of the sp^2^-IGLs to interfere in the immune system response in a cell line and cell context dependent manner.

## 1. Introduction

Since their conception in the mid-1990s, sp^2^-iminosugars have consolidated as a unique class of glycomimetics in terms of chemical and structural versatility. Examples on record include piperidine [1,2,3,4], pyrrolidine [5,6], pyrrolizidine [7,8], indolizidine [9,10,11,12,13,14], and *nor*-tropane cores [15,16,17] with varied hydroxylation profiles. The presence of a pseudoamide-type nitrogen, with substantial sp^2^-hybridized character, at the position of the ring oxygen in monosaccharides greatly facilitates the incorporation of substituents at the endocyclic heteroatom, providing a very convenient manner to modulate their properties as regulators of carbohydrate processing enzymes. This strategy has been exploited for the development of glycosidase activity enhancers as pharmacological chaperone candidates against several lysosomal storage disorders [18,19,20], including Gaucher [21,22,23,24,25,26], Fabry [27,28], G_M1_-gangliosidosis [29,30], Tay–Sachs [31], and α-mannosidosis [32] diseases. The pseudoamide functional group additionally influences the stereoelectronic properties at the pseudoanomeric region, which translates into an exacerbated anomeric effect that imparts a high chemical stability to axially-oriented heteroatom substituents. Thus, sp^2^-iminosugars exist in reducing form (anomeric OH) in aqueous solution and can engage in glycosylation reactions, thereby behaving as true chemical sugar mimics wherein the pseudoamide function exerts strict control of the stereochemical outcome to provide exclusively the α-anomer [33,34]. A variety of *O*-, *S*-, *N*-, and even *C*-glycoside derivatives [35,36], including sp^2^-iminosugar disaccharide mimetics [37], multivalent systems [38,39,40,41], glycopeptides (sp^2^-IGPs) [42], and glycolipids (sp^2^-IGLs) [43], have been prepared in this manner in single diastereomeric form. For comparison, *C*-glycosides are the only representatives that are stable in the case of classical iminosugars, but their stereoselective synthesis generally requires rather elaborated reaction sequences [44,45,46,47,48].

**Figure 1:**
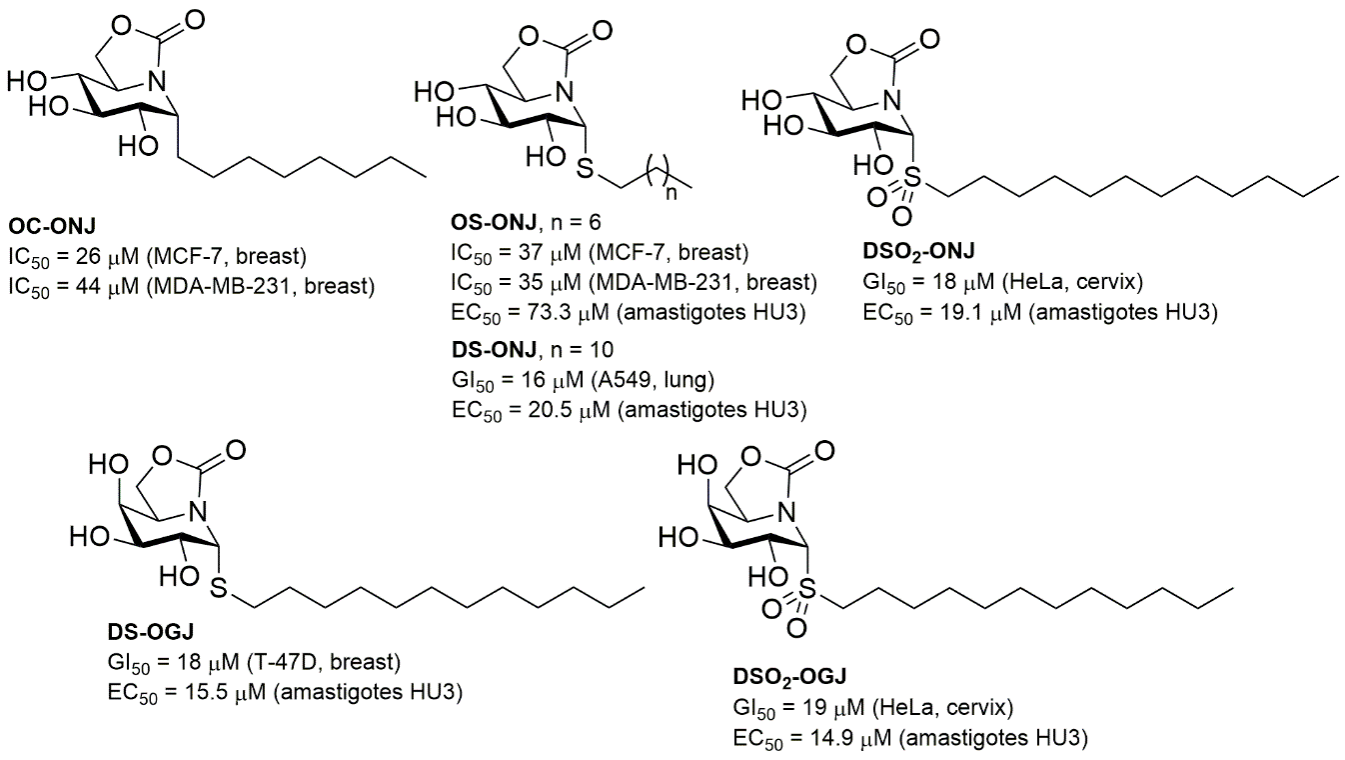
Chemical structures and biological activities of featured sp^2^-IGLs.

**Figure 2:**
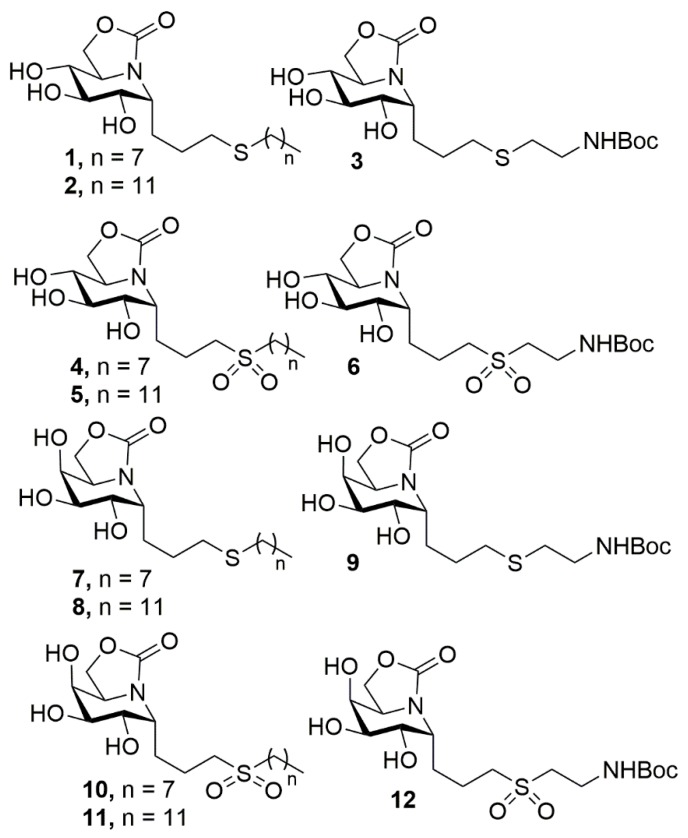
Chemical structures of the pseudo-α-*C*-glycoside sp^2^-IGLs prepared in this work.

The compatibility of the synthetic methodologies for the preparation of sp^2^-iminosugar conjugates with structural diversity-oriented strategies has contributed decisively to expand their range of biological activities. Notably, sp^2^-IGLs have shown to be potent antitumor, antileishmanial, and anti-inflammatory agents [49,50] depending on the nature of the aglycone moiety. For instance, the *C*-octyl pseudo-α-glycoside 5*N*,6*O*-oxomethylidenenojirimycin (ONJ) derivative OC-ONJ (Figure 1) exhibited selective antimitotic, proapoptotic, and antimetastatic activities against breast carcinoma in vitro (noninvasive MCF-7 and invasive MDA-MB-231 cell lines) and in vivo (mice) [51,52]. Replacement of the pseudoanomeric carbon atom by sulfur (OS-ONJ; Figure 1) additionally led to modest antiparasitic activity against intracellular amastigotes of *Leishmania donovani* (EC_50_ = 73.3 μM) [53]. Increasing the length of the *S*-alkyl chain up to twelve carbon atoms (DS-ONJ) and oxidation to the corresponding sulfone derivative (DSO_2_-ONJ) considerably improved the potency as leishmanicidal (up to 5-fold) without reducing the antiproliferative efficiency against different human tumor cell lines (GI_50_ < 20 μM). Although initially these behaviors were ascribed to the ability of inhibiting α-glucosidase, further evidence suggested that this might not be the case, as illustrated by the fact that the 5*N*,6*O*-oxomethylidenenojirimycin (OGJ) epimers (DS-OGJ and DSO_2_-OGJ, respectively, displayed similar antiproliferative and antiparasitic trends (Figure 1). Here we report the synthesis of new sp^2^-IGLs that combine the key structural features previously encountered responsible for the mentioned biological activities, namely a *C*-glycosidic linkage and the presence of sulfide or sulfone functionalities in the aglycone moiety by making use of the thiol-ene “click” reaction [54,55,56,57,58,59]. Precisely, the ONJ (**1**–**6**) and OGJ (**7**–**12**) derivatives (Figure 2) have been prepared and evaluated in parallel for their glycosidase inhibitory, antiproliferative and antileishmanial activities in an attempt to ascertain whether or not the mechanisms at play are concurrent.

## 2. Results and Discussion

### 2.1. Synthesis

**Scheme 1:**
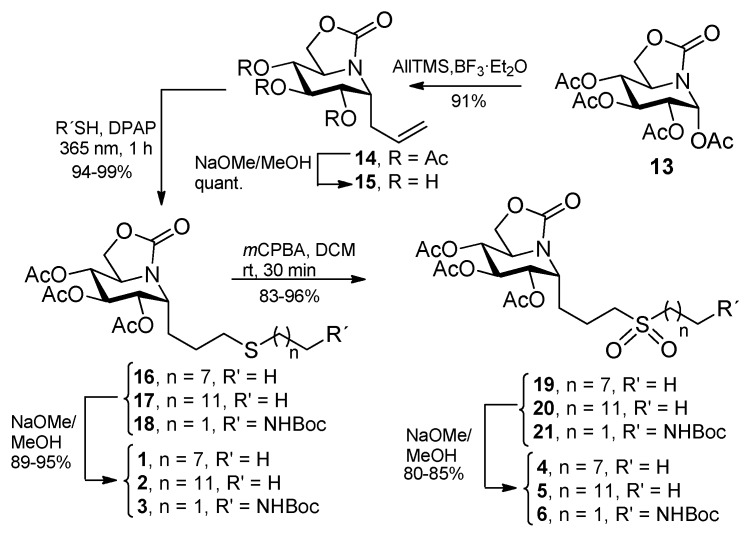
Synthesis of new ONJ sp^2^-IGLs **1**–**6**.

**Scheme 2:**
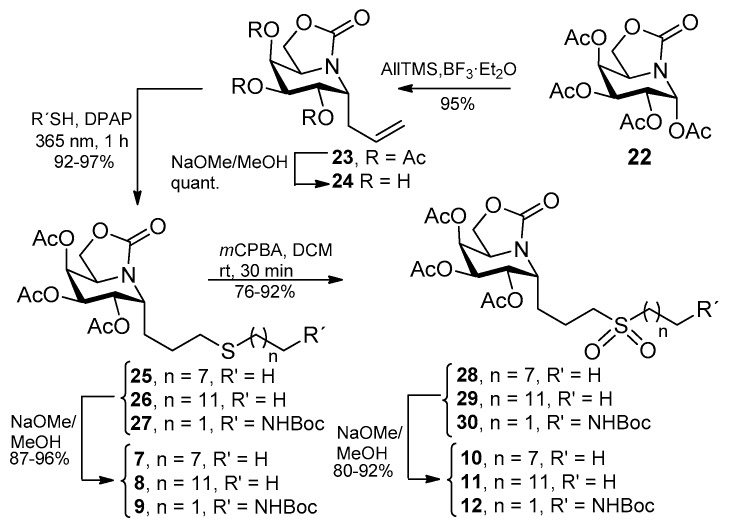
Synthesis of new OGJ sp^2^-IGLs (**7**–**12**).

The *C*-allylation reaction of the ONJ tetraacetate **13 [33]** by treatment with an excess of allyltrimethylsilane [60] and boron trifluoride etherate (BF_3_·OEt_2_) at 80 °C proceeded with total α-stereoselectivity to give the pivotal precursor **14** in high yield (Scheme 1). An aliquot of **14** was deacetylated to afford the unprotected *C*-allyl ONJ **15**, used as control in subsequent pharmacological tests. The corresponding NMR data were in full agreement with the proposed structure. Particularly, the vicinal coupling constant value between the anomeric H-1 proton and H-2 (6.0 and 6.6 Hz for **14** and **15**, respectively) support their gauche relative disposition characteristic of α-*gluco* arrangements in the ^4^*C*_1_ chair conformation. In order to access the target sp^2^-IGLs, photoinduced thiol-ene coupling reactions [61] of **14** with octanethiol, dodecanethiol, and 2-[*N*-(*tert*-butoxycarbonyl)amino]ethanethiol were conducted. The reaction conditions implied irradiation at 365 nm in the presence of a catalytic amount of 2,2-dimethoxy-2-phenyl-acetophenone (DPAP) in dry DMF, leading to the expected anti-Markovnikov sulfide adducts **16**, **17**, and **18**, respectively, in over 90% yield. Conventional deacetylation reactions provided the fully unprotected α-*C*-glycoside derivatives **1**, **2**, and **3** (Scheme 1). Compounds **16**–**18** were further oxidized with *m*-chloroperoxybenzoic acid (*m*CPBA) [53] to the corresponding sulfones **19**–**21**, which after removal of the acetate groups gave the amphiphilic sp^2^-IGLs **4**, **5**, and **6** (Scheme 1). A parallel reaction sequence starting from the OGJ tetraacetate **22**, let access the corresponding α-*C*-allyl glycoside (**23**, **24**) and α-*C*-glycoside sp^2^-IGL sulfide (**25**–**27** and **7**–**9**) and sulfone derivatives (**28**–**30** and **10**–**12**) with a substitution pattern of stereochemical complementarity to α-*C*-galactopyranosides (Scheme 2).

### 2.2. Inhibitory Properties against Commercial Enzymes

The inhibitory properties of the new sp^2^-IGLs **1**–**12** and the α-C-allyl ONJ and OGJ glycosides **15**, and **24** were examined against a panel of commercial glycosidases that included the α-glucosidases maltase (*Saccharomyces cerevisiae*) and amyloglucosidase (*Aspergillus niger*), the β-glucosidases from almonds and bovine liver, α-galactosidase from green coffee beans, β-galactosidase from *Escherichia coli*, α-mannosidase from Jack beans, and β-mannosidase from *Helix pomatia*. The corresponding inhibition constant (*K*_i_) values are collected in Table 1.

None of ONJ sp^2^-IGLs **1**–**6** showed inhibition towards α- or β-mannosidase or α- or β-galactosidase, in agreement with their d-*gluco* configurational pattern. They behaved instead as low-micromolar to nanomolar inhibitors of yeast maltase (*K*_i_ 2.6–0.28 μM). The presence of the lipidic aglycone was determinant for the inhibitory potency, since the parent α-*C*-allyl derivative **15** was a 30- to 100-fold weaker inhibitor of this enzyme (*K*_i_ 79 μM). The sulfone derivatives **4**–**6** behaved as less potent maltase inhibitors than the corresponding sulfides **1**–**3**, probably because of their moderated lipophilicity. Compounds **1**–**6** were much weaker inhibitors of β-glucosidase (bovine liver), with *K*_i_ values in the 54 to 400 μM range, meaning α:β anomeric selectivity ratios about 100:1. Neither epimeric OGJ sp^2^-IGLs **7**–**12** nor α-allyl OGJ glycoside **24** behaved as inhibitors of the α-galactosidase used in this assay, in spite of the configurational complementarity with the natural substrates. This is consistent with previous reports on the inhibitory properties of *galacto*-configured sp^2^-iminosugars showing that α-galactosidase inhibition is totally cancelled after engaging the primary hydroxyl in closing de five-membered ring [62]. As expected, the OGJ α-*C*-glycosides did not inhibit α-glucosidase, β-galactosidase or α- or β-mannosidase, although some representatives exhibited modest inhibition of the mammalian β-glucosidase (*K*_i_ 53 to 422 μM), an enzyme known to accept both β-d-*gluco* and β-d-*galacto* substrates [63].

### 2.3. Antiproliferative Activity

**Figure 3:**
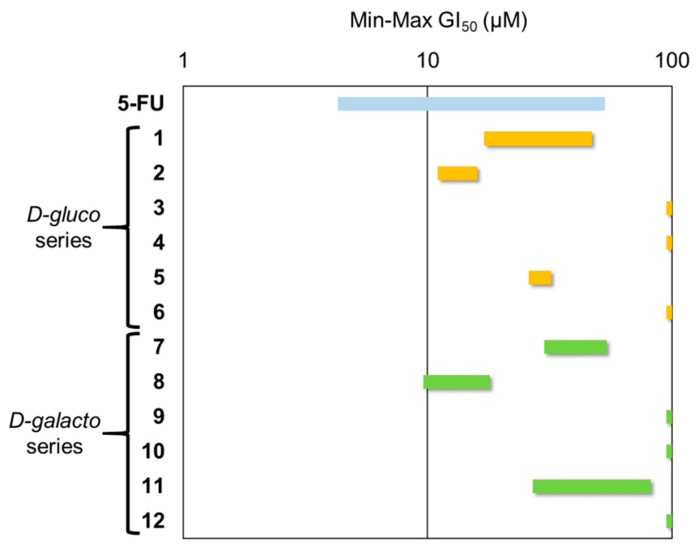
GI_50_ range plot of tested compounds. 5-Fluorouracil (5-FU) was used as reference drug.

The antiproliferative activity of all the sp^2^-iminosugar *C*-glycosides (**1**–**12**) was evaluated against a panel of six representative human solid tumor cell lines including lung (A549 and SW1573), breast (HBL-100, T-47D), cervix (HeLa), and colon (WiDr). The results, expressed as the concentration to achieve 50% growth inhibition (GI_50_), are shown in Figure 3; only GI_50_ values below 100 μM are considered significant.

The new α-*C*-glycoside sp^2^-IGLs included in this study were found not to affect the viability and mortality of the breast normal cell line (MCF-10A) at concentrations up to 100 μM. The ensemble of data revealed a strong dependence of the antiproliferative activity against tumor cells on the nature of the aglycone substituent. Comparatively, the effect of the configurational hydroxylation profile of the glycone moiety (d-*gluco* for the ONJ derivatives **1**–**6**; d-*galacto* for the OGJ derivatives **7**–**12**) is much less marked. The carbamate-containing sp^2^-IGLs **3**, **6**, **9**, and **12** did not show any significant antiproliferative activity in this assay. Conversely, the octyl (**1**,**7**) and dodecyl sulfides (**2**,**8**) were active against all the assayed tumor cell lines, with GI_50_ values ranging from 14 to 54 μM for the first pair and from 9.6 to 19 μM for the second pair, highlighting an increase in the antiproliferative activity with lipophilicity. This trend becomes much more pronounced in the sulfone series: whereas the octylsufonyl sp^2^-IGLs **4** and **10** did not achieve 50% tumor cell growth inhibition below the 100 μM concentration threshold, dodecylsulfonyl homologs **5** and **11** exhibited GI_50_ values in the range of 26 to 82 μM.

Inhibition of the α-glycosidases involved in *N*-glycoprotein biosynthesis, including α-glucosidases, has been invoked to explain the antitumor effects of some classical iminosugar glycomimetics [64,65]. Although the α-glucosidase inhibitory potency and antiproliferative activities of the ONJ derivatives both increased with aglycone lipophilicity, at least for the sulfide and sulfone representatives, close inspection of the data discards any direct relationship between them in the case of the sp^2^-IGLs. For instance, compounds **2** and **8** have very similar broad range antiproliferative profiles (Figure 3), but whereas **2** is a nanomolar competitive inhibitor of maltase, **8** did not inhibit this enzyme (Table 1). Even more revealing, compound **3**, the most potent α-glucosidase inhibitor of the library (*K*_i_ 0.28 μM), was out of range in the antiproliferative assay. These evidences strongly suggest that the mechanism responsible for the antiproliferative outcome of sp^2^-IGLs in cancer cell lines is glycosidase-independent.

### 2.4. Antileishmanial Activity and Cellular Toxicity 

The leishmanicidal activity of all the synthesized sp^2^-iminosugar *C*-glycosides (**1**–**12**) was evaluated against intracellular amastigotes of *Leishmania donovani* MHOM/ET/67/HU3 line with luciferase gene integrated into the parasite genome [66]. To this end, the concentration of compound required to inhibit the growth of parasites by 50% (50% effective concentration; EC_50_) was determined, with a threshold established at 20 μM (Table 2). A strong influence of the nature of the lipid chain α-linked to the pseudoanomeric position of the bicyclic core in the sp^2^-IGLs was inferred from the corresponding data. Thus, the 3-thiadodecyl (**1** and **7**) or the 3-thia-6-[*N*-(*tert*-butoxycarbonyl)amino]hexyl α-*C*-glycosides (**3** and **9**) promoted potent inhibition of the parasite growth, with EC_50_ values in the low micromolar range (7–15 μM), regardless of the configurational pattern of the sp^2^-iminosugar aglycone moiety (ONJ for **1** and **3**; DGJ for **7** and **9**). The longer-chain sp^2^-IGLs **2** and **8** were over the 20 μM concentration threshold in this assay. In the sulfone series, the results evidenced some disparity between the ONJ and OGJ epimers. Thus, the octylsulfonyl and dodecylsulfonyl segment-bearing derivatives **5** (EC_50_ 11.44 ± 5.08 μM) and **10** (EC_50_ 13.81 ± 1.72 μM) were the only representatives with EC_50_ values below the 20 μM threshold in the first and second case, respectively. Although the antileishmanial activity is modest as compared to the reference drug amphotericin B (AmB, Table 2), all sp^2^-IGLs exhibited lower toxicities in the monocytic leukemia (THP-1) cell line, broadly used as a model for human monocytes to assess potential toxic side-effects of antileishmanial drug candidates, and in human lung fibroblast MRC-5 cells (Table 2).

While it is apparent that the antiparasitic activity data in Table 2 do not correlate with the α-glucosidase inhibitory activity data in Table 1, it is less obvious to discern from the ensemble of results whether or not the antiproliferative and antileishmanial activities of the sp^2^-IGLs share common molecular mechanisms. The fact that both cancer and parasite infection lead to subversion of the innate immune system suggests that the compounds act as immunoregulators, as reported for other drugs displaying anticancer and antileishmanial behaviors, e.g., the alkyl lipid ethers miltefosine and perifosine [67]. Indeed, the sp^2^-IGL representative DSO_2_-ONJ (Figure 1), which also displayed antiproliferative and antileishmanial activities [43,53], was found to behave as a toll-like receptor-4 (TLR4) antagonist in LPS-induced inflammation models in microglia and dendritic cells, as well as in a mouse model of acute inflammation, displaying potent anti-inflammatory activity [49]. Biochemical and computational data pointed at the p38 mitogen activated protein kinase (MAPK), a master regulator of the immune response, as the putative target. Considering that p38 is also involved in cancer and parasite infection, it seems likely that sp^2^-IGL binding to p38 is responsible for the ensemble of the observed pharmacological properties. Notwithstanding, a comparative analysis of the results collected in Figure 3 and Table 2 reveals significant differences in the antiproliferative and antileishmanial activity trends of the new sp^2^-IGL **1**–**12** as a function of their structure. In both cases, the efficacy depends essentially of the aglycone nature, whereas the configuration of the glycone configuration—ONJ or OGJ—is much less influential. Yet, whereas the antitumor potency follows the tendency dictated by aglycone lipophilicity, the antiparasitic efficiency is less predictable and more sensitive to the aglycone structure. For instance, the derivatives combining a sulfide and a carbamate group in the lipid chain (**3** and **9**), which did not display significant antiproliferative activity against any of the solid tumor cells assayed (see previous section), are low micromolar antileishmanial agents (EC_50_ 14.96 ± 1.28 and 13.39 ± 1.13 μM, respectively). In contrast, the oxidized sulfone counterparts **6** and **12** were over the 20 μM threshold in the antileishmanial assayed. It should be considered that other factors, such as membrane-crossing or membrane-insertion capabilities, can strongly influence the biological activities of glycolipids in a cell line and cell-context dependent manner and can be also at the origin of the differences observed between both assays.

In summary, the results here presented demonstrate the suitability of the *C*-allylation of sp^2^-iminosugars in combination with the thiol-ene click reaction to access α-*C*-glycoside sp^2^-IGLs in high yield and with total stereocontrol, providing a versatile strategy to assess their pharmacological properties. The glycosidase inhibitory, antiproliferative and antileishmanial activities can be then optimized either individually or concertedly. The biological data support that the capabilities to inhibit glycosidases and the abilities to act as immunoregulators in the context of cancer or parasite infection are independent, opening the possibility of purposely designing multitarget compounds. It is conceivable, for instance, that sp^2^-IGLs behaving concomitantly as immunostimulants and inhibitors of a glycosidase involved in cancer or parasite cell metabolism will lead to more efficient therapeutics. Work in this direction is currently sought in our laboratories.

## 3. Materials and Methods

### 3.1. General Methods

Reagents and solvents were purchased from commercial sources and used without further purification. Optical rotations were measured with a JASCO P-2000 polarimeter, using a sodium lamp (λ = 589 nm) at 22 °C in 1 cm or 1 dm tubes. NMR experiments were performed at 300 (75.5), 400 (100.6) and 500 (125.7) MHz. 1-D TOCSY, as well as 2-D COSY and HMQC, experiments were carried out to assist signal assignment. For ESI mass spectra, 0.1 pm sample concentrations were used, the mobile phase consisting of 50% aq MeCN at 0.1 mL/min. Thin-layer chromatography was performed on precoated TLC plates, silica gel 30F-245, with visualization by UV light and also with 10% H_2_SO_4_ or 0.2% *w*/*v* cerium (IV) sulfate–5% ammonium molybdate in 2 m H_2_SO_4_, or 0.1% ninhydrin in EtOH. Column chromatography was performed on Chromagel (silice 60 AC.C 70–200 μm). Deacetylation reactions were carried out by using Zemplén procedure, e.i., addition of NaOMe (0.1 equiv. per mol of acetate) in MeOH at room temperature and neutralization with solid CO_2_, evaporation of the solvent and purification by column chromatography. All compounds were purified to ≥95% purity as determined by elemental microanalysis results obtained on a CHNS-TruSpect^®^ Micro elemental analyzer (Instituto de Investigaciones Químicas de Sevilla, Spain) from vacuum-dried samples. The analytical results for C, H, N and S were within ± 0.4 of the theoretical values. (1*R*)-1,2,3,4-Tetra-*O*-acetyl-5*N*,6*O*-oxomethylidenegluco(galacto)nojirimycin (**13** and **22**, respectively) were prepared according reported procedures [33,68].

Inhibition constant (*K*_i_) values were determined by spectrophotometrically measuring the residual hydrolytic activities of the glycosidases against the respective *o*- (for β-galactosidase from *E. coli*) or *p*-nitrophenyl α- or β-d-glycopyranoside (for other glycosidases) in the presence of the iminosugars. Each assay was performed in phosphate buffer or phosphate-citrate buffer (for α- or β-mannosidase and amyloglucosidase) at the optimal pH for the enzymes. The reactions were initiated by addition of the enzyme to a solution of the substrate in the absence or presence of various inhibitor concentrations. The mixture was incubated for 10–30 min at 37 °C or 55 °C (for amyloglucosidase) and the reaction was quenched by addition of 1 m Na_2_CO_3_. Reaction times were appropriate to obtain 10–20% conversion of the substrate in order to achieve linear rates. The absorbance of the resulting mixture was determined at 405 nm. Approximate value of *K*_i_ was determined from the slope of Dixon plots using a fixed concentration of substrate (around the *K*_m_ value for the different glycosidases) and various concentrations of inhibitor. Full *K*_i_ determinations and enzyme inhibition mode were determined from the slope of Lineweaver–Burk plots and double reciprocal analysis. Representative examples of Dixon and Lineweaver–Burk plots are shown in the Supplementary materials.

### 3.2. Statistical Analysis

All results are expressed as mean ± SD of three independent experiments, each conducted in triplicate. The measurements were statistically analyzed using the Student’s *t*-test for comparing 2 groups. The level of significance was set at *p* < 0.05.

### 3.3. General Procedure for the Synthesis of the Allylated ONJ and OGJ Precursors ***14*** and ***23***

To a solution of **13**/**22** (2.20 mmol) in anhydrous CH_3_CN (36 mL) under Ar atmosphere, allyltrimethylsilane (3.5 mL, 22 mmol) and BF_3_.Et_2_O (2.8 mL) were added at rt and the mixture was then heated at 80 °C for 20 min. The reaction mixture was diluted with EtOAc (50 mL) and quenched with saturated aqueous NaHCO_3_ (2 × 30 mL). The organic phase was separated and the aqueous phase extracted with EtOAc (3 × 25 mL). The combined organic layer was washed with water (25 mL) and brine (25 mL), dried (MgSO_4_) and concentrated under reduced pressure. The resulting residue was purified by column chromatography using the solvent indicated in each case to give the corresponding *C*-allylated pseudo-α-glycosides.

(1*R*)-2,3,4-Tri-*O*-acetyl-1-allyl-5*N*,6*O*-oxomethylidene-1-deoxynojirimycin (**14**). Purification by column chromatography (1:1 EtOAc-cyclohexane) afforded **14**. Yield**:** 712 mg (91%); *R_f_* = 0.60 (2:1 EtOAc-cyclohexane). [α]_D_ + 34.0 (*c* 1.0 in DCM); ^1^H NMR (500 MHz, CDCl_3_) δ 5.72 (dddd, 1 H, *J*_c,d_ = 17.0 Hz, *J*_c,e_ = 10.0 Hz, *J*_c,b_ = 8.0 Hz, *J*_c,a_ = 5.5 Hz, C*H*(c)=CH_2_), 5.34 (t, 1 H, *J*_2,3_ = *J*_3,4_ = 10.0 Hz, H-3), 5.14 (d, 1 H, CH=C*H*_2_(d)), 5.11 (d, 1 H, CH=C*H*_2_(e)), 5.06 (dd, 1 H, *J*_1,2_ = 6.0 Hz, H-2), 4.93 (t, 1 H, *J*_4,5_ = 10.0 Hz, H-4), 4.41–4.33 (m, 2 H, H-1, H-6a), 4.22 (dd, 1 H, *J*_6a,6b_ = 9.5 Hz, *J*_5,6b_ = 5.0 Hz, H-6b), 3.81 (ddd, 1 H, *J*_5,6a_ = 8.5 Hz, H-5), 2.52 (dt, 1 H, *J*_a,b_ = 15.0 Hz, *J*_a,1_ = 5.0 Hz, C*H*_2(_a)CH=CH_2_), 2.34 (ddd, 1 H, *J*_b,1_ = 11.5 Hz, C*H*_2_(b)CH=CH_2_), 2.05–2.02 (3 s, 9 H, MeCO); ^13^C NMR (125.7 MHz, CDCl_3_) δ 170.0-169.2 (CO ester), 156.2 (CO carbamate), 132.8 (*C*H=CH_2_), 118.5 (CH=*C*H_2_), 72.5 (C-4), 69.9–69.5 (C-2, C-3), 65.7 (C-6), 52.1 (C-5), 50.0 (C-1), 30.7 (*C*H_2_CH=CH_2_), 20.6 (*Me*CO); ESIMS: *m*/*z* 378.28 [M + Na]^+^; Anal. Calcd for C_16_H_21_NO_8_: C 54.08, H 5.96, N 3.94; found: C 54.22, H 6.08, N 3.71. 

(1*R*)-2,3,4-Tri-*O*-acetyl-1-allyl-5*N*,6*O*-oxomethylidene-1-deoxygalactonojirimycin (**23**). Purification by column chromatography (1:2 → 2:1 EtOAc-cyclohexane) afforded **23**. Yield: 344 mg (95%); *R_f_* = 0.63 (2:1 EtOAc-cyclohexane); [α]_D_ + 64.7 (*c* 1.0 in DCM); ^1^H NMR (300 MHz, CDCl_3_) δ 5.71 (dddd, 1 H, *J*_c,d_ = 17.1 Hz, *J*_c,e_ = 10.2 Hz, *J*_c,b_ = 8.1 Hz, *J*_c,a_ = 5.4 Hz, C*H*(c)=CH_2_), 5.38 (t, 1 H, *J*_3,4_ = *J*_4,5_ = 2.1 Hz, H-4), 5.30 (dd, 1 H, *J*_2,3_ = 10.8 Hz, *J*_1,2_ = 6.6 Hz, H-2), 5.12 (m, 3 H, H-3, CH=C*H*_2_), 4.49 (ddd, 1 H, *J*_1,b_ = 11.1 Hz, *J*_1,a_ = 4.5 Hz, H-1), 4.31 (t, 1 H, *J*_6a,6b_ = *J*_5,6a_ = 8.5 Hz, H-6a), 4.02 (m, 2 H, H-5, H-6b), 2.48 (m, 1 H, C*H*_2(_a)CH=CH_2_), 2.27 (ddd, 1 H, *J*_a,b_ = 15.0 Hz, *J*_b,H1_ = 11.5 Hz, C*H*_2_(b)CH=CH_2_), 2.15–1.98 (3 s, 9 H, MeCO); ^13^C NMR (75.5 MHz, CDCl_3_) δ 170.5-169.3 (CO ester), 156.6 (CO carbamate), 133.2 (*C*H=CH_2_), 118.3 (CH=*C*H_2_), 68.9 (C-4), 68.5 (C-3), 66.5 (C-2), 62.6 (C-6), 51.1 (C-5), 50.3 (C-1), 30.4 (*C*H_2_CH=CH_2_), 20.7-20.6 (*Me*CO); ESIMS: *m*/*z* 378.26 [M + Na]^+^; Anal. Calcd for C_16_H_21_NO_8_: C 54.08, H 5.96, N 3.94. Found: C 54.11, H 6.03, N 3.90.

(1*R*)-1-Allyl-5*N*,6*O*-oxomethylidene-1-deoxynojirimycin (**15**). Compound **15** was obtained by conventional *O*-deacetylation of **14** (51 mg, 0.14 mmol), as described in general methods, followed by purification by column chromatography (25:1 EtOAc-MeOH). Yield: 33 mg (quant); *R_f_* = 0.26 (25:1 EtOAc-MeOH); [α]_D_ + 22.0 (*c* 1.1 in MeOH); ^1^H NMR (300 MHz, CD_3_OD) δ 5.76 (dddd, 1 H, *J*_c,d_ = 17.1 Hz, *J*_c,e_ = 10.2 Hz, *J*_c,a_ = 8.1 Hz, *J*_c,b_ = 5.7 Hz, C*H*(c)=CH_2_), 5.14 (dd, 1 H, *J*_d,b_ = 1.2 Hz, CH=C*H*_2_(d)), 5.05 (d, 1 H, CH=C*H*_2_(e)), 4.43 (t, 1 H, *J*_6a,6b_ = *J*_5,6a_ = 8.5 Hz, H-6a), 4.27 (dd, 1 H, *J*_5,6b_ = 4.2 Hz, H-6b), 4.00 (ddd, 1 H, *J*_1,a_ = 12.3 Hz, *J*_1,2_ = 5.4 Hz, *J*_1,b_ = 3.6 Hz, H-1), 3.65 (ddd, 1 H, *J*_4,5_ = 9.5 Hz, H-5), 3.57–3.43 (m, 2 H, H-2, H-3), 3.24 (t, 1 H, *J*_3,4_ = 9.0 Hz, H-4), 2.66 (dddd, 1 H, *J*_a,b_ = 15.0 Hz, C*H*_2_(b)CH=CH_2_), 2.21 (ddd, 1 H, C*H*_2_(a)CH=CH_2_); ^13^C NMR (75.5 MHz, CD_3_OD) δ 159.5 (CO), 136.0 (*C*H=CH_2_), 117.7 (CH=*C*H_2_), 75.5 (C-4), 74.7–72.0 (C-2, C-3), 67.5 (C-6), 55.2–55.0 (C-1, C-5), 30.5 (CH_2_CH=CH_2_); ESIMS: *m*/*z* 252.2 [M + Na]^+^; Anal. Calcd for C_10_H_15_NO_5_: C 52.40, H 6.60, N 6.11. Found: C 52.28, H 6.73, N 5.87.

(1*R*)-1-Allyl-5*N*,6*O*-oxomethylidene-1-deoxygalactonojirimycin (**24**). Compound **24** was obtained by conventional *O*-deacetylation of **23** (33 mg, 0.09 mmol) followed by purification by column chromatography (30:1 EtOAc-MeOH). Yield: 21 mg (quant); *R_f_* = 0.22 (30:1 EtOAc-MeOH); [α]_D_ − 3.0 (*c* 1.0 in MeOH); ^1^H NMR (300 MHz, CDCl_3_) δ 5.78 (dddd, 1 H, *J*_c,d_ = 18.3 Hz, *J*_c,e_ = 10.2 Hz, *J*_c,b_ = 8.3 Hz, *J*_c,a_ = 5.6 Hz, C*H*(c)=CH_2_), 5.15 (m, 1 H, CH=C*H*_2_(d)), 5.06 (m, 1 H, CH=C*H*_2_(e)), 4.41 (dd, 1 H, *J*_6a,6b_ = 8.6 Hz, *J*_5,6a_ = 4.6 Hz, H-6a), 4.34 (t, 1 H, *J*_5,6b_ = 8.6 Hz, H-6b), 4.08 (m, 1 H, H-1), 4.02 (ddd, 1 H, *J*_4,5_ = 2.3 Hz, H-5), 3.96 (dd, 1 H, *J*_2,3_ = 9.9 Hz, *J*_1,2_ = 6.4 Hz, H-2), 3.82 (t, 1 H, *J*_3,4_ = 2.3 Hz, H-4), 3.65 (dd, 1 H, H-3), 2.65 (m, 1 H, C*H*_2_(b)CH=CH_2_), 2.24 (ddd, 1 H, *J*_a,b_ = 14.6 Hz, *J*_b,H1_ = 12.0 Hz, C*H*_2_(a)CH=CH_2_); ^13^C NMR (75.5 MHz, CDCl_3_) δ 160.3 (CO), 136.4 (*C*H=CH_2_), 117.6 (CH=*C*H_2_), 71.5 (C-3), 71.1 (C-4), 68.1 (C-2), 64.8 (C-6), 54.9 (C-5), 54.5 (C-1), 30.1 (*C*H_2_CH=CH_2_); ESIMS: *m*/*z* 252.20 [M + Na]^+^; Anal. Calcd for C_10_H_15_NO_5_: C 52.40, H 6.60, N 6.11. Found: C 52.09, H 6.38, N 5.85. 

### 3.4. General Procedure for the Synthesis of ONJ (***1**–**3***) and OGJ Derivatives (***7**–**9***) by Photoinduced Thiol-Ene Reaction 

To a stirred solution of the corresponding pseudo-α-C-allyl glycosides **14**/**23** (142 mg, 0.40 mmol) and 2,2-dimethoxy-2-phenyl-acetophenone (DPAP) (31 mg, 0.12 mmol) in anhydrous DMF (2.5 mL), the corresponding thiol (1.20 mmol) was added. The mixture was deoxygenated for 30 min and then irradiated at rt under UVA lamp (λ 365 nm) for 1 h. The solvent was removed and the residue was purified by column chromatography using (1:5 → 1:3 →1:1 EtOAc:cyclohexane) as eluent.

(1*R*)-2,3,4-Tri-O-acetyl-1-[3-(octylthio)propyl]-5N,6O-oxomethylidene-1-deoxynojirimycin (**16**). Yield: 70 mg (99%); *R_f_*_=_ 0.74 (2:1 EtOAc-cyclohexane); [α]_D_ + 42.2 (c 1.0 in DCM); ^1^H NMR (300 MHz, CDCl_3_) δ 5.31 (t, 1 H, *J*_2,3_ = *J*_3,4_ = 10.0 Hz, H-3), 5.01 (dd, 1 H, *J*_1,2_ = 6.3 Hz, H-2), 4.94 (t, 1 H, *J*_4,5_ = 10.0 Hz, H-4), 4.38 (dd, 1 H, *J*_6a,6b_ = 9.0 Hz, *J*_5,6a_ = 8.1 Hz, H-6a), 4.31–4.20 (m, 1 H, H-1), 4.25 (dd, 1 H, *J*_5,6b_ = 4.5 Hz, H-6b), 3.85 (1 H, ddd, H-5), 2.66–2.43 (m, 4 H, CH_2_SCH_2_), 2.00–1.96 (3 s, 9 H, MeCO), 1.90–1.20 (m, 16 H, CH_2_), 0.87 (t, 3 H, ^3^*J*_H,H_ = 7.0 Hz, CH_3_); ^13^C NMR (75.5 MHz, CDCl_3_) δ 169.9–169.2 (CO ester), 156.3 (CO carbamate), 72.3 (C-4), 69.8 (C-3), 69.6 (C-2), 65.6 (C-6), 52.1 (C-5), 50.7 (C-1), 32.2–22.6 (CH_2_), 20.6 (MeCO), 14.1 (CH_3_); ESIMS: *m*/*z* 524.39 [M + Na]^+^; Anal. Calcd for C_24_H_39_NO_8_S: C 57.46, H 7.84, N 2.79, S, 6.39. Found: C 57.61, H 8.00, N 2.48, S, 6.13. 

(1*R*)-2,3,4-Tri-*O*-acetyl-1-[3-(dodecylthio)propyl]-5*N*,6*O*-oxomethylidene-1-deoxynojirimycin (**17**). Yield: 118 mg (96%); *R_f_* = 0.58 (1:1 EtOAc-cyclohexane). [α]_D_ + 37.5 (c 1.1 in DCM); ^1^H NMR (300 MHz, CDCl_3_) δ 5.30 (t, 1 H, *J*_2,3_ = *J*_3,4_ = 9.6 Hz, H-3), 5.00 (dd, 1 H, *J*_1,2_ = 6.3 Hz, H-2), 4.94 (t, 1 H, *J*_4,5_ = 9.6 Hz, H-4), 4.37 (dd, 1 H, *J*_6a,6b_ = 9.0 Hz, *J*_5,6a_ = 8.1 Hz, H-6a), 4.30–4.20 (m, 1 H, H-1), 4.23 (dd, 1 H, *J*_5,6b_ = 4.5 Hz, H-6b), 3.84 (1 H, ddd, H-5), 2.65–2.42 (m, 4 H, CH_2_SCH_2_), 2.05–2.01 (3 s, 9 H, MeCO), 1.90–1.20 (m, 24 H, CH_2_), 0.86 (t, 3 H, ^3^*J*_H,H_ = 7.0 Hz, CH_3_); ^13^C NMR (75.5 MHz, CDCl_3_) δ 169.9–169.2 (CO ester), 156.3 (CO carbamate), 72.3 (C-4), 69.8 (C-3), 69.5 (C-2), 65.6 (C-6), 52.1 (C-5), 50.7 (C-1), 32.2–22.7 (CH_2_), 20.6–20.5 (MeCO), 14.1 (CH_3_); ESIMS: *m*/*z* 580.43 [M + Na]^+^; Anal. Calcd for C_28_H_47_NO_8_S: C 60.30, H 8.49, N 2.51, S 5.75. Found: C 60.54, H 8.63, N 2.36, S 5.49. 

(1*R*)-2,3,4-Tri-*O*-acetyl-1-[3-(*N*-tert-butoxycarbonylaminoethylthio)propyl]-5*N*,6*O*-oxomethylidene-1-deoxynojirimycin (**18**). Yield: 218 mg (94%); *R_f_* = 0.39 (1:1 EtOAc-cyclohexane); [α]_D_ + 36.5 (c 1.1 in DCM); ^1^H NMR (300 MHz, CDCl_3_) δ 5.30 (t, 1 H, *J*_2,3_ = *J*_3,4_ = 9.6 Hz, H-3), 5.00 (dd, 1 H, *J*_1,2_ = 6.0 Hz, H-2), 4.95 (t, 1 H, *J*_4,5_ = 9.6 Hz, H-4), 4.40 (dd, 1 H, *J*_6a,6b_ = 9.3 Hz, *J*_5,6a_ = 8.1 Hz, H-6a), 4.30–4.20 (m, 2 H, H-1, H-6b), 3.84 (ddd, 1 H, *J*_5,6b_ = 4.2 Hz, H-5), 3.28 (q, 2 H, *J* = 6.5 Hz, CH_2_NHBoc), 2.70–2.46 (m, 4 H, CH_2_SCH_2_), 2.06–2.01 (3 s, 9 H, MeCO), 1.90–1.57 (m, 4 H, CH_2_), 1.43 (s, 9 H, CMe_3_); ^13^C NMR (75.5 MHz, CDCl_3_) δ 169.9–169.2 (CO ester), 156.3, 155.8 (CO carbamate), 79.4 (CMe_3_), 72.3 (C-4), 69.8 (C-3), 69.6 (C-2), 65.6 (C-6), 52.0 (C-5), 50.5 (C-1), 39.8 (CH_2_NHBoc), 32.1, 30.9 (CH_2_), 28.4 (CMe_3_), 25.3, 24.0 (CH_2_), 20.7–20.6 (MeCO); ESIMS: *m*/*z* 555.28 [M + Na]^+^; Anal. Calcd for C_23_H_36_N_2_O_10_S: C 51.87, H 6.81, N 5.26, S 6.02. Found: C 51.71, H 6.99, N 4.97, S 5.78. 

(1*R*)-2,3,4-Tri-*O*-acetyl-1-[3-(octylthio)propyl]-5*N*,6*O*-oxomethylidene-1-deoxygalactonojirimycin (**25**). Yield: 188 mg (94%); *R_f_* = 0.59 (1:1 EtOAc-cyclohexane); [α]_D_ + 77.7 (c 1.0 in DCM); ^1^H NMR (300 MHz, CDCl_3_) δ 5.37 (t, 1 H, *J*_3,4_ = *J*_4,5_ = 2.7 Hz, H-4), 5.25 (dd, 1 H, *J*_2,3_ = 10.9 Hz, *J*_1,2_ = 6.3 Hz, H-2), 5.10 (dd, 1 H, H-3), 4.34 (m, 1 H, H-1), 4.33 (t, 1 H, *J*_6a,6b_ = *J*_5,6a_ = 9.0 Hz, H-6a), 4.10 (m, 1 H, H-5), 4.01 (dd, 1 H, *J*_5,6b_ = 3.5 Hz, H-6b), 2.52 (m, 4 H, CH_2_SCH_2_), 2.14–1.95 (3 s, 9 H, MeCO), 1.83–1.47 (m, 6 H, CH_2_), 1.32 (m, 10 H, CH_2_), 0.84 (t, 3 H, ^3^*J*_H,H_ = 7.0 Hz, CH_3_); ^13^C NMR (75.5 MHz, CDCl_3_) δ 170.4–169.4 (CO ester), 156.8 (CO carbamate), 69.0 (C-4), 68.4 (C-3), 66.5 (C-2), 62.3 (C-6), 51.0 (C-5), 50.8 (C-1), 32.1–22.6 (CH_2_), 20.7–20.5 (MeCO), 14.1 (CH_3_); ESIMS: *m*/*z* 524.33 [M + Na]^+^; Anal. Calcd for C_24_H_39_NO_8_S: C 57.46, H 7.84, N 2.79. S, 6.39. Found: C 57.57, H 8.03, N 2.56, S 6.04.

(1*R*)-2,3,4-Tri-*O*-acetyl-1-[3-(dodecylthio)propyl]-5*N*,6*O*-oxomethylidene-1-deoxygalactonojirimycin (**26**). Yield: 205 mg (92%); *R_f_* = 0.69 (1:1 EtOAc-cyclohexane); [α]_D_ + 63.8 (c 1.0 in DCM); ^1^H NMR (300 MHz, CDCl_3_) δ 5.34 (t, 1 H, *J*_3,4_ = *J*_4,5_ = 2.7 Hz, H-4), 5.23 (dd, 1 H, *J*_2,3_ = 10.9 Hz, *J*_1,2_ = 6.4 Hz, H-2), 5.07 (dd, 1 H, H-3), 4.30 (m, 1 H, H-1), 4.29 (t, 1 H, *J*_6a,6b_ = *J*_5,6a_ = 8.9 Hz, H-6a), 4.04 (m, 1 H, H-5), 3.97 (dd, 1 H, *J*_5,6b_ = 3.5 Hz, H-6b), 2.49 (m, 4 H, CH_2_S), 2.11–1.93 (3 s, 9 H, MeCO), 1.85–1.45 (m, 6 H, CH_2_), 1.25 (m, 18 H, CH_2_), 0.81 (t, 3 H, ^3^*J*_H,H_ = 7.0 Hz, CH_3_); ^13^C NMR (75.5 MHz, CDCl_3_) δ 170.5–169.4 (CO ester), 156.8 (CO carbamate), 69.0 (C-4), 68.4 (C-3), 66.5 (C-2), 62.8 (C-6), 51.1 (C-5), 50.9 (C-1), 32.2–22.7 (CH_2_), 20.7–20.5 (MeCO), 14.1 (CH_3_); ESIMS: *m*/*z* 580.38 [M + Na]^+^; Anal. Calcd for C_28_H_47_NO_8_S: C 60.30, H 8.49, N 2.51, S 5.75. Found: C, 60.17, H 8.42, N 2.29, S 5.60.

(1*R*)-2,3,4-Tri-*O*-acetyl-1-[3-(*N*-tert-butoxycarbonylaminoethylthio)propyl]-5*N*,6*O*-oxomethylidene-1-deoxygalactonojirimycin (**27**). Yield: 207 mg (97%); *R_f_* = 0.44 (2:1 EtOAc-cyclohexane); [α]_D_ + 64.1 (c 1.0 in DCM); ^1^H NMR (300 MHz, CDCl_3_) δ 5.34 (t, 1 H, *J*_3,4_ = *J*_4,5_ = 2.7 Hz, H-4), 5.23 (dd, 1 H, *J*_2,3_ = 10.9 Hz, *J*_1,2_ = 6.4 Hz, H-2), 5.10 (dd, 1 H, H-3), 4.88 (bs, 1 H, NH), 4.33 (m, 1 H, H-1), 4.33 (t, 1 H, *J*_6a,6b_ = *J*_5,6a_ = 8.9 Hz, H-6a), 4.04 (m, 1 H, H-5), 3.97 (dd, 1 H, *J*_5,6b_ = 3.5 Hz, H-6b), 3.23 (q, 2 H, ^3^*J*_H,H_ = 6.5 Hz, CH_2_NHBoc), 2.50 (m, 4 H, CH_2_SCH_2_), 2.11–1.93 (3 s, 9 H, MeCO), 1.80–1.50 (m, 4 H, CH_2_), 1.38 (s, 9 H, CMe_3_); ^13^C NMR (75.5 MHz, CDCl_3_) δ 170.4–169.4 (CO ester), 156.8, 155.8 (CO carbamate), 79.4 (CMe_3_), 69.0 (C-4), 68.3 (C-3), 66.5 (C-2), 62.8 (C-6), 51.1 (C-5), 50.7 (C-1), 39.8 (CH_2_NHBoc), 32.1, 30.9 (CH_2_), 28.4 (CMe_3_), 25.4, 23.6 (CH_2_), 20.8-20.6 (MeCO); ESIMS: *m*/*z* 555.30 [M + Na]^+^. Anal. Calcd for C_23_H_36_N_2_O_10_S: C 51.87, H 6.81, N 5.26, S 6.02. Found: C 51.60, H 6.95, N 5.09, S 5.71.

(1*R*)-1-[3-(Octylthio)propyl)-5*N*,6*O*-oxomethylidene-1-deoxynojirimycin (**1**). Compound **1** was obtained by conventional O-deacetylation of **16** (70 mg, 0.14 mmol), as described in general methods, followed by purification by column chromatography (40:1 EtOAc-MeOH). Yield: 50 mg (95%); *R_f_* = 0.47 (30:1 EtOAc-MeOH); [α]_D_ + 43.9 (c 1.1 in MeOH); ^1^H NMR (300 MHz, CD_3_OD) δ 4.45 (t, 1 H, *J*_6a,6b_ = *J*_5,6a_ = 9.0 Hz, H-6a), 4.29 (dd, 1 H, *J*_5,6b_ = 4.0 Hz, H-6b), 3.96–3.86 (m, 1 H, H-1), 3.66 (ddd, 1 H, *J*_4,5_ = 9.6 Hz, H-5), 3.53–3.40 (m, 2 H, H-2, H-3), 3.28–3.18 (m, 1 H, H-4), 2.66–2.46 (m, 4 H, CH_2_SCH_2_), 2.07–1.93 (m, 1 H, CH_2_), 1.72–1.23 (m, 15 H, CH_2_), 0.90 (t, 3 H, ^3^*J*_H,H_ = 7.0 Hz, CH_3_); ^13^C NMR (75.5 MHz, CD_3_OD) δ 159.6 (CO), 75.4 (C-4), 74.6–72.1 (C-2, C-3), 67.5 (C-6), 55.3 (C-1, C-5), 33.0–23.7 (CH_2_), 14.4 (CH_3_); ESIMS: *m*/*z* 398.38 [M + Na]^+^; Anal. Calcd for C_18_H_33_NO_5_S: C 57.57, H 8.86, N 3.73, S 8.54. Found: C 57.22, H 8.76, N 3.52, S 8.16. 

(1*R*)-1-[3-(Dodecylthio)propyl]-5*N*,6*O*-oxomethylidene-1-deoxynojirimycin (**2**). Compound **2** was obtained by conventional *O*-deacetylation of **17** (59 mg, 0.10 mmol), as described in general methods, followed by purification by column chromatography (30:1 EtOAc-MeOH). Yield: 41mg (89%); *R_f_* = 0.47 (30:1 EtOAc-MeOH); [α]_D_ + 42.6 (c 1.0 in MeOH); ^1^H NMR (400 MHz, CD_3_OD) δ 4.45 (dd, 1 H, *J*_6a,6b_ = 9.0 Hz, *J*_5,6a_ = 8.4 Hz, H-6a), 4.29 (dd, 1 H, *J*_5,6b_ = 4.0 Hz, H-6b), 3.95–3.88 (m, 1 H, H-1), 3.66 (ddd, 1 H, *J*_4,5_ = 9.5 Hz, H-5), 3.51–3.41 (m, 2 H, H-2, H-3), 3.23 (bt, 1 H, H-4), 2.65–2.48 (m, 4 H, CH_2_SCH_2_), 2.06–1.95 (m, 1 H, CH_2_), 1.70–1.25 (m, 23 H, CH_2_), 0.90 (t, 3 H, ^3^*J*_H,H_ = 7.0 Hz, CH_3_); ^13^C NMR (100.6 MHz, CD_3_OD) δ 159.7 (CO), 75.4 (C-4), 74.6–72.2 (C-2, C-3), 67.5 (C-6), 55.3 (C-1, C-5), 33.1–23.7 (CH_2_), 14.4 (CH_3_); ESIMS: *m*/*z* 454.40 [M + Na]^+^; Anal. Calcd for C_22_H_41_NO_5_S: C 61.22, H 9.57, N 3.25, S 7.43. Found: C 60.88, H 9.33, N 2.99, S 7.15. 

(1*R*)-1-[3-(*N*-*tert*-Butoxycarbonylaminoethylthio)propyl]-5*N*,6*O*-oxomethylidene-1-deoxynojirimycin (**3**). Compound **3** was obtained by conventional O-deacetylation of **18** (66 mg, 0.12 mmol), as described in general methods, followed by purification by column chromatography (30:1 EtOAc-MeOH). Yield: 47 mg (92%); *R_f_* = 0.45 (30:1 EtOAc-MeOH); [α]_D_ + 36.2 (c 1.0 in MeOH); ^1^H NMR (300 MHz, CD_3_OD) δ 4.47 (t, 1 H, *J*_6a,6b_ = *J*_5,6a_ = 8.5 Hz, H-6a), 4.29 (dd, 1 H, *J*_5,6b_ = 4.0 Hz, H-6b), 3.97–3.82 (m, 1 H, H-1), 3.66 (ddd, 1 H, *J*_4,5_ = 9.6 Hz, H-5), 3.53–3.40 (m, 2 H, H-2, H-3), 3.28–3.11 (m, 3 H, H-4, CH_2_NHBoc), 2.70–2.50 (m, 4 H, CH_2_SCH_2_), 2.05–1.91 (m, 1 H, CH_2_), 1.72–1.49 (m, 3 H, CH_2_), 1.44 (s, 9 H, CMe_3_); ^13^C NMR (75.5 MHz, CD_3_OD) δ 159.7, 158.3 (CO), 80.1 (CMe_3_), 75.4 (C-4), 74.6–72.1 (C-2, C-3), 67.5 (C-6), 55.3 (C-1, C-5), 41.4 (CH_2_NHBoc), 32.5–24.5 (CH_2_), 28.8 (CMe_3_); ESIMS: *m*/*z* 429.31 [M + Na]^+^; Anal. Calcd for C_17_H_30_N_2_O_7_S: C 50.23, H 7.44, N 6.89, S 7.89. Found: C 50.37, H 7.60, N 6.58, S 7.61. 

(1*R*)-1-[3-(Octylthio)propyl]-5*N*,6*O*-oxomethylidene-1-deoxygalactonojirimycin (**7**). Compound **7** was obtained by conventional *O*-deacetylation of **25** (103 mg, 0.21 mmol) followed by purification by column chromatography (30:1 EtOAc-MeOH). Yield: 74 mg (96%); *R_f_* = 0.19 (30:1 EtOAc-MeOH); [α]_D_ + 31.0 (c 1.1 in MeOH); ^1^H NMR (300 MHz, CD_3_OD) δ 4.31 (dd, 1 H, *J*_6a,6b_ = 8.6 Hz, *J*_5,6a_ = 4.6 Hz, H-6a), 4.26 (t, 1 H, *J*_5,6b_ = 8.6 Hz, H-6b), 3.91 (m, 2 H, H-1, H-5), 3.81 (dd, 1 H, *J*_2,3_ = 9.7 Hz, *J*_1,2_ = 6.4 Hz, H-2), 3.71 (t, 1 H, *J*_3,4_ = *J*_4,5_ = 2.3 Hz, H-4), 3.51 (dd, 1 H, H-3), 2.46 (m, 4 H, CH_2_SCH_2_), 1.95–1.36 (m, 6 H, CH_2_), 1.26 (m, 10 H, CH_2_), 0.80 (t, 3 H, ^3^*J*_H,H_ = 6.9 Hz, CH_3_); ^13^C NMR (75.5 MHz, CD_3_OD) δ 160.4 (CO), 71.4 (C-3), 71.1 (C-4), 68.1 (C-2), 64.9 (C-6), 55.1 (C-5), 54.6 (C-1), 33.0–23.7 (CH_2_), 14.5 (CH_3_); ESIMS: *m*/*z* 398.36 [M + Na]^+^; Anal. Calcd for C_18_H_33_NO_5_S: C 57.57, H 8.86, N 3.73, S 8.54. Found: C 57.26, H 8.64, N 3.46, S 8.23. 

(1*R*)-1-[3-(Dodecylthio)propyl]-5*N*,6*O*-oxomethylidene-1-deoxygalactonojirimycin (**8**). Compound **8** was obtained by conventional *O*-deacetylation of **26** (86 mg, 0.15 mmol) followed by purification by column chromatography (30:1 EtOAc-MeOH). Yield: 58 mg (87%); *R_f_* = 0.31 (30:1 EtOAc-MeOH); [α]_D_ + 27.2 (c 1.1 in MeOH); ^1^H NMR (300 MHz, CD_3_OD) δ 4.43 (dd, 1 H, *J*_6a,6b_ = 8.6 Hz, *J*_5,6a_ = 4.4 Hz, H-6a), 4.37 (t, 1 H, *J*_5,6b_ = 8.6 Hz, H-6b), 4.02 (m, 2 H, H-1, H-5), 3.93 (dd, 1 H, *J*_2,3_ = 9.7 Hz, *J*_1,2_ = 6.4 Hz, H-2), 3.82 (t, 1 H, *J*_3,4_ = *J*_4,5_ = 2.3 Hz, H-4), 3.62 (dd, 1 H, H-3), 2.65–2.49 (m, 4 H, CH_2_SCH_2_), 2.04–1.38 (m, 6 H, CH_2_), 1.37 (m, 18 H, CH_2_), 0.98 (t, 3 H, ^3^*J*_H,H_ = 6.9 Hz, CH_3_); ^13^C NMR (75.5 MHz, CD_3_OD) δ 160.4 (CO), 71.4 (C-3), 71.1 (C-4), 68.1 (C-2), 64.9 (C-6), 55.1 (C-5), 54.6 (C-1), 33.1–23.7 (CH_2_), 14.4 (CH_3_); ESIMS: *m*/*z* 454.39 [M + Na]^+^; Anal. Calcd for C_22_H_41_NO_5_S: C 61.22, H 9.57, N 3.25, S 7.43. Found: C 60.95, H 9.28, N 2.98, S 7.14. 

(1*R*)-1-[3-(*N*-*tert*-Butoxycarbonylaminoethylthio)propyl]-5*N*,6*O*-oxomethylidene-1-deoxygalactonojirimycin (**9**). Compound **9** was obtained by conventional *O*-deacetylation of **27** (72 mg, 0.14 mmol) followed by purification by column chromatography (30:1 EtOAc-MeOH). Yield: 52 mg (95%); *R_f_* = 0.15 (30:1 EtOAc-MeOH); [α]_D_ + 26.2 (c 1.0 in MeOH); ^1^H NMR (300 MHz, CD_3_OD) δ 4.31 (dd, 1 H, *J*_6a,6b_ = 8.5 Hz, *J*_5,6a_ = 4.9 Hz, H-6a), 4.27 (t, 1 H, *J*_5,6b_ = 8.5 Hz, H-6b), 3.95 (m, 2 H, H-1, H-5), 3.82 (dd, 1 H, *J*_2,3_ = 9.7 Hz, *J*_1,2_ = 6.4 Hz, H-2), 3.71 (t, 1 H, *J*_3,4_ = *J*_4,5_ = 2.3 Hz, H-4), 3.51 (dd, 1 H, H-3), 3.11 (t, 2 H, ^3^*J*_H,H_ = 6.9 Hz, CH_2_NHBoc), 2.59–2.41 (m, 4 H, CH_2_SCH_2_), 1.93–1.40 (m, 6 H, CH_2_), 1.34 (s, 9 H, CMe_3_); ^13^C NMR (75.5 MHz, CD_3_OD) δ 160.4, 158.3 (CO), 80.1 (CMe_3_), 71.4 (C-3), 71.1 (C-4), 68.1 (C-2), 64.9 (C-6), 55.1 (C-5), 54.6 (C-1), 41.4 (CH_2_NHBoc), 32.5, 32.2 (CH_2_), 28.8 (CMe_3_), 27.5, 24.0 (CH_2_); ESIMS: *m*/*z* 429.26 [M + Na]^+^; Anal. Calcd for C_17_H_30_N_2_O_7_S: C 50.23, H 7.44, N 6.89, S 7.89. Found: C 50.01, H 7.33, N 6.58, S 7.62. 

### 3.5. General Procedure for the Synthesis of Sulfone Derivatives of ONJ ***4**–**6*** and OGJ ***10**–**12***

To a solution of the corresponding sulfide precursor (**16**–**18**, **25**–**27**) (0.13 mmol) in DCM (3 mL), 70% mCPBA (0.26 mmol) was added at 0 °C. The reaction mixture was stirred for 30–60 min, diluted with DCM (50 mL), washed with aqueous NaHCO_3_ (2 × 10 mL), brine (10 mL), dried (MgSO_4_), and concentrated. The resulting residue was purified by column chromatography using the solvent indicated in each case.

(1*R*)-2,3,4-Tri-*O*-acetyl-1-[3-(octylsulfonyl)propyl]-5*N*,6*O*-oxomethylidene-1-deoxynojirimycin (**19**). Column chromatography (1:1 EtOAc-cyclohexane). Yield: 179 mg (96%); *R_f_* = 0.50 (2:1 EtOAc-cyclohexane); [α]_D_ + 41.3 (c 0.9 in DCM); ^1^H NMR (300 MHz, CDCl_3_) δ 5.27 (t, 1 H, *J*_2,3_ = *J*_3,4_ = 9.6 Hz, H-3), 5.03 (dd, 1 H, *J*_1,2_ = 6.0 Hz, H-2), 4.96 (t, 1 H, *J*_4,5_ = 9.6 Hz, H-4), 4.40 (t, 1 H, *J*_6a,6b_ = *J*_5,6a_ = 8.5 Hz, H-6a), 4.32–4.20 (m, 1 H, H-1), 4.24 (dd, 1 H, *J*_5,6b_ = 4.5 Hz, H-6b), 3.86 (ddd, 1 H, H-5), 3.14–2.86 (m, 4 H, CH_2_SO_2_CH_2_), 2.04–1.99 (3 s, 9 H, MeCO), 1.97–1.18 (m, 16 H, CH_2_), 0.85 (t, 3 H, ^3^*J*_H,H_ = 7.0 Hz, CH_3_); ^13^C NMR (75.5 MHz, CDCl_3_) δ 169.9–169.3 (CO ester), 156.5 (CO carbamate), 72.2 (C-4), 69.6 (C-3), 69.3 (C-2), 65.8 (C-6), 53.4 (CH_2_SO_2_), 52.0 (C-5), 50.9 (CH_2_SO_2_), 50.2 (C-1), 31.7–18.2 (CH_2_), 20.6–20.5 (MeCO), 14.1 (CH_3_); ESIMS: *m*/*z* 556.29 [M + Na]^+^; Anal. Calcd for C_24_H_39_NO_10_S: C 54.02, H 7.37, N 2.62, S 6.01. Found: C 54.24, H 7.50, N 2.36, S 5.77.

(1*R*)-2,3,4-Tri-*O*-acetyl-1-[3-(dodecylsulfonyl)propyl]-5*N*,6*O*-oxomethylidene-1-deoxynojirimycin (**20**). Column chromatography (1:1 EtOAc-cyclohexane). Yield: 46.5 mg (87%); *R_f_* = 0.52 (2:1 EtOAc-cyclohexane); [α]_D_ + 30.7 (c 1.1 in DCM); ^1^H NMR (300 MHz, CDCl_3_) δ 5.27 (t, 1 H, *J*_2,3_ = *J*_3,4_ = 10.0 Hz, H-3), 5.03 (dd, 1 H, *J*_1,2_ = 6.0 Hz, H-2), 4.95 (t, 1 H, *J*_4,5_ = 9.6 Hz, H-4), 4.40 (t, 1 H, *J*_6a,6b_ = *J*_5,6a_ = 8.5 Hz, H-6a), 4.33–4.20 (m, 1 H, H-1), 4.24 (dd, 1 H, *J*_5,6b_ = 4.5 Hz, H-6b), 3.86 (ddd, 1 H, H-5), 3.15–2.87 (m, 4 H, CH_2_SO_2_CH_2_), 2.04–2.00 (3 s, 9 H, MeCO), 1.96–1.18 (m, 24 H, CH_2_), 0.85 (t, 3 H, ^3^*J*_H,H_ = 7.0 Hz, CH_3_); ^13^C NMR (75.5 MHz, CDCl_3_) δ 169.9–169.3 (CO ester), 156.5 (CO carbamate), 72.2 (C-4), 69.6 (C-3), 69.3 (C-2), 65.8 (C-6), 53.4 (CH_2_SO_2_), 52.0 (C-5), 50.9 (CH_2_SO_2_), 50.2 (C-1), 31.9–18.2 (CH_2_), 20.6–20.5 (MeCO), 14.1 (CH_3_); ESIMS: *m*/*z* 612.47 [M + Na]^+^; Anal. Calcd for C_28_H_47_NO_10_S: C 57.03, H 8.03, N 2.38, S 5.44. Found: C 57.28, H 8.17, N 2.06, S 5.24. 

(1*R*)-2,3,4-Tri-*O*-acetyl-1-[3-(*N*-*tert*-butoxycarbonylaminoethylsulfonyl)propyl]-5*N*,6*O*-oxomethylidene-1-deoxynojirimycin (**21**). Column chromatography (1:1 EtOAc-cyclohexane). Yield: 60 mg (83%); *R_f_* = 0.23 (2:1 EtOAc-cyclohexane); [α]_D_ + 30.7 (c 1.1 in DCM); ^1^H NMR (300 MHz, CDCl_3_) δ 5.30 (t, 1 H, *J*_2,3_ = *J*_3,4_ = 10.2 Hz, H-3), 5.04 (dd, 1 H, *J*_1,2_ = 6.3 Hz, H-2), 4.97 (t, 1 H, *J*_4,5_ = 9.5 Hz, H-4), 4.43 (t, 1 H, *J*_6a,6b_ = *J*_5,6a_ = 8.4 Hz, H-6a), 4.34–4.22 (m, 2 H, H-1, H-6b), 3.87 (ddd, 1 H, *J*_5,6b_ = 4.5 Hz, H-5), 3.60 (q, 2 H, *J* = 6.0 Hz, CH_2_NHBoc), 3.23–2.93 (m, 4 H, CH_2_SO_2_CH_2_), 2.06–2.01 (3 s, 9 H, MeCO), 1.98–1.76 (m, 4 H, CH_2_), 1.43 (s, 9 H, CMe_3_); ^13^C NMR (75.5 MHz, CDCl_3_) δ 169.9–169.3 (CO ester), 156.6, 155.7 (CO carbamate), 80.0 (CMe_3_), 72.2 (C-4), 69.6 (C-3), 69.3 (C-2), 65.9 (C-6), 53.0 (CH_2_SO_2_), 52.0, 51.9 (C-5, CH_2_SO_2_), 50.0 (C-1), 34.4 (CH_2_NHBoc), 28.3 (CMe_3_), 23.9 (CH_2_), 20.6–20.5 (MeCO), 18.1 (CH_2_); ESIMS: *m*/*z* 587.22 [M + Na]^+^; Anal. Calcd for C_23_H_36_N_2_O_12_S: C 48.93, H 6.43, N 4.96, S 5.68. Found: C 49.14, H 6.50, N 4.79, S 5.46. 

(1*R*)-2,3,4-Tri-*O*-acetyl-1-[3-(octylsulfonyl)propyl]-5*N*,6*O*-oxomethylidene-1-deoxygalactonojirimycin (**28**). Column chromatography (1:1 → 2:1 EtOAc-cyclohexane). Yield: 79 mg (92%); *R_f_* = 0.34 (2:1 EtOAc-cyclohexane); [α]_D_ + 5.6 (c 1.0 in DCM); ^1^H NMR (300 MHz, CDCl_3_) δ 5.42 (t, 1 H, *J*_3,4_ = *J*_4,5_ = 2.7 Hz, H-4), 5.32 (dd, 1 H, *J*_2,3_ = 10.9 Hz, *J*_1,2_ = 6.5 Hz, H-2), 5.13 (dd, 1 H, H-3), 4.43 (m, 1 H, H-1), 4.42 (t, 1 H, *J*_6a,6b_ = *J*_5,6a_ = 8.9 Hz, H-6a), 4.14 (m, 1 H, H-5), 4.07 (dd, 1 H, *J*_5,6b_ = 3.5 Hz, H-6b), 3.16–2.93 (m, 4 H, CH_2_SO_2_CH_2_), 2.20–2.02 (3 s, 9 H, MeCO), 2.12–1.78 (m, 6 H, CH_2_), 1.37 (m, 10 H, CH_2_), 0.90 (t, 3 H, ^3^*J*_H,H_ = 7.0 Hz, CH_3_); ^13^C NMR (75.5 MHz, CDCl_3_) δ 170.4–169.5 (CO ester), 157.0 (CO carbamate), 68.9 (C-4), 68.3 (C-3), 66.4 (C-2), 63.0 (C-6), CH_2_SO_2_ (53.4), 51.0 (C-5), 50.9 (C-1), 50.4 (CH_2_SO_2_), 31.9–21.9 (CH_2_), 20.7–20.6 (MeCO), 18.4 (CH_2_), 14.0 (CH_3_); ESIMS: *m*/*z* 556.27 [M + Na]^+^; Anal. Calcd for C_24_H_39_NO_10_S: C 54.02, H 7.37, N 2.62, S 6.01. Found: C 53.88, H 7.42, N 2.41, S 5.69. 

(1*R*)-2,3,4-Tri-*O*-acetyl-1-[3-(dodecylsulfonyl)propyl]-5*N*,6*O*-oxomethylidene-1-deoxygalactonojirimycin (**29**). Column chromatography (1:1 → 2:1 EtOAc-cyclohexane). Yield: 72 mg (76%); *R_f_* = 0.45 (2:1 EtOAc-cyclohexane); [α]_D_ + 5.8 (c 1.0 in DCM); ^1^H NMR (300 MHz, CDCl_3_) δ 5.34 (m, 1 H, H-4), 5.23 (dd, 1 H, *J*_2,3_ = 10.9 Hz, *J*_1,2_ = 6.4 Hz, H-2), 5.10 (dd, 1 H, *J*_3,4_ = 2.6 Hz, H-3), 4.34 (m, 1 H, H-1), 4.33 (t, 1 H, *J*_6a,6b_ = *J*_5,6a_ = 9.0 Hz, H-6a), 4.08 (m, 1 H, H-5), 3.98 (dd, 1 H, *J*_5,6b_ = 3.4 Hz, H-6b), 3.13–2.85 (m, 4 H, CH_2_SO_2_CH_2_), 2.11–1.93 (3 s, 9 H, MeCO), 1.84–1.30 (m, 6 H, CH_2_), 1.19 (m, 18 H, CH_2_), 0.81 (t, 3 H, ^3^*J*_H,H_ = 6.9 Hz, CH_3_); ^13^C NMR (75.5 MHz, CDCl_3_) δ 170.4, 170.0, 169.5 (CO ester), 157.0 (CO carbamate), 68.9 (C-4), 68.2 (C-3), 66.4 (C-2), 62.9 (C-6), 53.5 (CH_2_SO_2_), 51.0 (C-5), 50.8 (C-1), 50.4 (CH_2_SO_2_), 31.9–21.9 (CH_2_), 20.7–20.5 (MeCO), 18.4 (CH_2_), 14.1 (CH_3_); ESIMS: *m*/*z* 612.26 [M + Na]^+^; Anal. Calcd for C_24_H_47_NO_10_S: C 57.03, H 8.03, N 2.38, S 5.44. Found: C 57.14, H 8.23, N 2.17, S 5.16.

(1*R*)-2,3,4-Tri-*O*-acetyl-1-[3-(*N*-*tert*-butoxycarbonylaminoethylsulfonyl)propyl]-5*N*,6*O*-oxomethylidene-1-deoxygalactonojirimycin (**30**). Column chromatography (1:1 → 2:1 → 3:1 EtOAc-cyclohexane). Yield: 77 mg (85%); *R_f_* = 0.24 (2:1 EtOAc-cyclohexane); [α]_D_ + 44.8 (c 1.1 in DCM); ^1^H NMR (300 MHz, CDCl_3_) δ 5.34 (m, 1 H, H-4), 5.23 (dd, 1 H, *J*_2,3_ = 10.9 Hz, *J*_1,2_ = 6.4 Hz, H-2), 5.05 (dd, 1 H, *J*_3,4_ = 2.6 Hz, H-3), 4.37 (m, 1 H, H-1), 4.36 (t, 1 H, *J*_6a,6b_ = *J*_5,6a_ = 9.0 Hz, H-6a), 4.08 (m, 1 H, H-5), 3.98 (dd, 1 H, *J*_5,6b_ = 3.4 Hz, H-6b), 3.54 (q, 2 H, ^3^*J*_H,H_ = 6.0 Hz, CH_2_NHBoc), 3.15–2.90 (m, 4 H, CH_2_SO_2_CH_2_), 2.12–1.93 (3 s, 9 H, MeCO), 1.99–1.66 (m, 4 H, CH_2_), 1.37 (s, 9 H, CMe_3_); ^13^C NMR (75.5 MHz, CDCl_3_) δ 170.4–169.5 (CO ester), 157.0, 155.8 (CO carbamate), 80.0 (CMe_3_), 68.9 (C-4), 68.2 (C-3), 66.3 (C-2), 63.0 (C-6), 53.0, 51.9 (CH_2_SO_2_), 51.0 (C-5), 50.3 (C-1), 34.5 (CH_2_NHBoc), 29.7 (CH_2_), 28.3 (CMe_3_), 23.5 (CH_2_), 20.7–20.5 (MeCO); ESIMS: *m*/*z* 587.24 [M + Na]^+^; Anal. Calcd for C_23_H_36_N_2_O_12_S: C 48.93, H 6.43, N 4.96, S 5.68. Found: C 49.11, H 6.51, N 4.72, S 5.46.

(1*R*)-1-[3-(Octylsulfonyl)propyl]-5*N*,6*O*-oxomethylidene-1-deoxynojirimycin (**4**). Compound **4** was obtained by conventional *O*-deacetylation of **19** (96 mg, 0.18 mmol), as described in general methods, followed by purification by column chromatography (30:1 EtOAc-MeOH). Yield: 62 mg (85%); *R_f_* = 0.27 (30:1 EtOAc-MeOH); [α]_D_ + 51.8 (c 0.8 in MeOH); ^1^H NMR (300 MHz, CD_3_OD) δ 4.48 (t, 1 H, *J*_6a,6b_ = *J*_5,6a_ = 8.5 Hz, H-6a), 4.30 (dd, 1 H, *J*_5,6b_ = 3.9 Hz, H-6b), 3.93 (ddd, 1 H, *J*_1,CH_ = 11.7 Hz, *J*_1,2_ = 5.7 Hz, *J*_1,CH´_ = 3.3 Hz, H-1), 3.69 (ddd, 1 H, *J*_4,5_ = 9.6 Hz, H-5), 3.55–3.40 (m, 2 H, H-2, H-3), 3.29–3.00 (m, 5 H, H-4, CH_2_SO_2_CH_2_), 2.10–1.58 (m, 6 H, CH_2_), 1.52–1.22 (m, 10 H, CH_2_), 0.91 (t, 3 H, ^3^*J*_H,H_ = 7.0 Hz, CH_3_); ^13^C NMR (75.5 MHz, CD_3_OD) δ 159.7 (CO), 75.3 (C-4), 74.6, 72.0 (C-2, C-3), 67.6 (C-6), 55.2 (C-5), 55.0 (C-1), 53.5, 52.6 (CH_2_SO_2_CH_2_), 32.9–19.8 (CH_2_), 14.4 (CH_3_); ESIMS: *m*/*z* 430.32 [M + Na]^+^; Anal. Calcd for C_18_H_33_NO_7_S: C 53.05, H 8.16, N 3.44, S 7.87. Found: C 52.81, H 7.98, N 3.11, S 7.49.

(1*R*)-1-[3-(Dodecylsulfonyl)propyl]-5*N*,6*O*-oxomethylidene-1-deoxynojirimycin (**5**). Compound **5** was obtained by conventional *O*-deacetylation of **20** (34 mg, 0.058 mmol), as described in general methods, followed by purification by column chromatography (50:1 → 30:1 → 15:1 EtOAc-MeOH). Yield: 22 mg (83%); *R_f_* = 0.33 (30:1 EtOAc-MeOH); [α]_D_ + 34.4 (c 0.9 in MeOH); ^1^H NMR (300 MHz, CD_3_OD) δ 4.48 (t, 1 H, *J*_6a,6b_ = *J*_5,6a_ = 8.5 Hz, H-6a), 4.30 (dd, 1 H, *J*_5,6b_ = 3.9 Hz, H-6b), 3.93 (ddd, 1 H, ^3^*J*_1,CH_ = 11.7 Hz, *J*_1,2_ = 5.7 Hz, ^3^*J*_1,CH´_ = 3.0 Hz, H-1), 3.69 (ddd, 1 H, *J*_4,5_ = 10.0 Hz, H-5), 3.55–3.39 (m, 2 H, H-2, H-3), 3.29–3.14 (m, 2 H, H-4, CH_2_SO_2_), 3.12–3.00 (m, 3 H, CH_2_SO_2_CH_2_), 2.10–1.25 (m, 24 H, CH_2_), 0.90 (t, 3 H, ^3^*J*_H,H_ = 7.0 Hz, CH_3_); ^13^C NMR (75.5 MHz, CD_3_OD) δ 159.8 (CO), 75.4 (C-4), 74.6, 72.0 (C-2, C-3), 67.6 (C-6), 55.3 (C-5), 55.1 (C-1), 53.5, 52.7 (CH_2_SO_2_CH_2_), 33.1–19.8 (CH_2_), 14.4 (CH_3_); ESIMS: *m*/*z* 486.41 [M + Na]^+^. Anal. Calcd for C_22_H_41_NO_7_S: C 56.99, H 8.91, N 3.02, S 6.91. Found: C 56.67, H 8.73, N 2.78, S 6.53.

(1*R*)-1-[3-(*N*-*tert*-Butoxycarbonylaminoethylsulfonyl)propyl]-5*N*,6*O*-oxomethylidene-1-deoxynojirimycin (**6**). Compound **6** was obtained by conventional *O*-deacetylation of **21** (35.6 mg, 0.06 mmol), as described in general methods, followed by purification by column chromatography (30:1 → 10:1 EtOAc:MeOH). Yield: 22 mg (80%); *R_f_* = 0.33 (10:1 EtOAc-MeOH); [α]_D_ + 26.5 (c 1.0 in MeOH); ^1^H NMR (300 MHz, CD_3_OD) δ 4.49 (dd, 1 H, *J*_6a,6b_ = 9.0 Hz, *J*_5,6a_ = 8.1 Hz, H-6a), 4.30 (dd, 1 H, *J*_5,6b_ = 3.9 Hz, H-6b), 3.93 (ddd, 1 H, ^3^*J*_1,CH_ = 11.7 Hz, *J*_1,2_ = 5.7 Hz, ^3^*J*_1,CH´_ = 3.0 Hz, H-1), 3.69 (ddd, 1 H, *J*_4,5_ = 9.6 Hz, H-5), 3.54–3.39 (m, 4 H, H-2, H-3, CH_2_NHBoc), 3.29–3.05 (m, 5 H, H-4, CH_2_SO_2_CH_2_), 2.10–1.58 (m, 4 H, CH_2_), 1.44 (s, 9 H, CMe_3_); ^13^C NMR (75.5 MHz, CD_3_OD) δ 159.8 (CO), 80.6 (CMe_3_), 75.3 (C-4), 74.6, 72.0 (C-2, C-3), 67.6 (C-6), 55.3 (C-5), 55.1 (C-1), 53.5, 53.1 (CH_2_SO_2_CH_2_), 35.3 (CH_2_NHBoc), 28.7 (CMe_3_), 24.6–19.8 (CH_2_); ESIMS: *m*/*z* 461.22 [M + Na]^+^; Anal. Calcd for C_17_H_30_N_2_O_9_S: C 46.57, H 6.90, N 6.39, S 7.31. Found: C 46.23, H 6.69, N 6.08, S 6.95.

(1*R*)-1-[3-(Octylsulfonyl)propyl]-5*N*,6*O*-oxomethylidene-1-deoxygalactonojirimycin (**10**). Compound **10** was obtained by conventional *O*-deacetylation of **28** (73 mg, 0.14 mmol) followed by purification by column chromatography (50:1 → 30:1 → 15:1 EtOAc-MeOH). Yield: 50 mg (90%); *R_f_* = 0.30 (9:1 EtOAc-MeOH); [α]_D_ + 29.6 (c 1.0 in MeOH); ^1^H NMR (500 MHz, 3:7 CDCl_3_-CD_3_OD) δ 4.43 (dd, 1 H, *J*_6a,6b_ = 8.6 Hz, *J*_5,6a_ = 4.3 Hz, H-6a), 4.39 (t, 1 H, *J*_5,6b_ = 8.6 Hz, H-6b), 4.00 (m, 2 H, H-1, H-5), 3.95 (dd, 1 H, *J*_2,3_ = 10.0 Hz, *J*_1,2_ = 6.5 Hz, H-2), 3.82 (t, 1 H, *J*_3,4_ = 2.8 Hz, H-4), 3.60 (dd, 1 H, H-3), 3.21 (ddd, 1 H, ^2^*J*_H,H_ = 15.1 Hz, ^3^*J*_H,H_ = 9.7 Hz, ^3^*J*_H,H_ = 5.6 Hz,CHSO_2_), 3.03 (m, 3 H, CHSO_2_, CH_2_SO_2_), 2.06–1.61 (m, 6 H, CH_2_), 1.50–1.28 (m, 10 H, CH_2_), 0.90 (t, 3 H, ^3^*J*_H,H_ = 7.1 Hz, CH_3_); ^13^C NMR (125.7 MHz, 3:7 CDCl_3_-CD_3_OD) δ 160.0 (CO), 71.0 (C-3), 70.5 (C-4), 67.5 (C-2), 64.5 (C-6), 55.3 (C-5), 54.1 (C-1), 53.4, 52.4 (CH_2_SO_2_CH_2_), 32.5–19.6 (CH_2_), 14.3 (CH_3_); ESIMS: *m*/*z* 430.32 [M + Na]^+^; Anal. Calcd for C_18_H_33_NO_7_S: C 53.05, H 8.16, N 3.44, S 7.87. Found: C 52.74, H 7.89, N 3.19, S 7.51.

(1*R*)-1-[3-(Dodecylsulfonyl)propyl]-5*N*,6*O*-oxomethylidene-1-deoxygalactonojirimycin (**11**). Compound **11** was obtained by conventional *O*-deacetylation of **29** (56 mg, 0.09 mmol) followed by purification by column chromatography (50:1 → 30:1 → 15:1 EtOAc-MeOH). Yield: 35 mg (80%); *R_f_* = 0.30 (30:1 EtOAc-MeOH); [α]_D_ + 26.0 (c 1.1 in MeOH); ^1^H NMR (500 MHz, 3:7 CDCl_3_-CD_3_OD) δ 4.44 (dd, 1 H, *J*_6a,6b_ = 8.6 Hz, *J*_5,6a_ = 4.2 Hz, H-6a), 4.39 (t, 1 H, *J*_5,6b_ = 8.6 Hz, H-6b), 4.00 (m, 2 H, H-1, H-5), 3.95 (dd, 1 H, *J*_2,3_ = 10.0 Hz, *J*_1,2_ = 6.5 Hz, H-2), 3.82 (t, 1 H, *J*_3,4_ = 2.8 Hz, H-4), 3.60 (dd, 1 H, H-3), 3.21 (ddd, 1 H, ^2^*J*_H,H_ = 15.1 Hz, ^3^*J*_H,H_ = 9.6 Hz, ^3^*J*_H,H_ = 5.6 Hz,CHSO_2_), 3.03 (m, 3 H, CHSO_2_, CH_2_SO_2_), 2.03–1.63 (m, 6 H, CH_2_), 1.50–1.29 (m, 18 H, CH_2_), 0.89 (t, 3 H, ^3^*J*_H,H_ = 6.8 Hz, CH_3_); ^13^C NMR (125.7 MHz, 3:7 CDCl_3_-CD_3_OD) δ 160.0 (CO), 71.1 (C-3), 70.6 (C-4), 67.5 (C-2), 64.6 (C-6), 54.4 (C-5), 54.2 (C-1), 53.4, 52.4 (CH_2_SO_2_CH_2_), 32.7–19.6 (CH_2_), 14.4 (CH_3_); ESIMS: *m*/*z* 486.38 [M + Na]^+^; Anal. Calcd for C_22_H_41_NO_7_S: C 56.99, H 8.91, N 3.02, S 6.91. Found: C 56.87, H 8.79, N 2.85, S 6.70.

(1*R*)-1-[3-(*N*-*tert*-Butoxycarbonylaminoethylsulfonyl)propyl]-5*N*,6*O*-oxomethylidene-1-deoxygalactonojirimycin (**12**). Compound **12** was obtained by conventional *O*-deacetylation of **30** (66 mg, 0.12 mmol) followed by purification by column chromatography (30:1 → 10:1 → 5:1 EtOAc-MeOH). Yield: 47 mg (92%); *R_f_* = 0.29 (9:1 EtOAc-MeOH); [α]_D_ + 20.7 (c 1.0 in MeOH); ^1^H NMR (500 MHz, 3:7 CDCl_3_-CD_3_OD) δ 4.35 (dd, 1 H, *J*_6a,6b_ = 8.5 Hz, *J*_5,6a_ = 4.7 Hz, H-6a), 4.31 (t, 1 H, *J*_5,6b_ = 8.5 Hz, H-6b), 3.92 (m, 2 H, H-1, H-5), 3.85 (dd, 1 H, *J*_2,3_ = 9.7 Hz, *J*_1,2_ = 6.5 Hz, H-2), 3.72 (t, 1 H, *J*_3,4_ = 2.7 Hz, H-4), 3.50 (dd, 1 H, H-3), 3.44 (t, 2 H, ^3^*J*_H,H_ = 6.7 Hz, CH_2_NHBoc), 3.15 (m, 3 H, CHSO_2_, CH_2_SO_2_), 2.98 (ddd, 1 H, ^2^*J*_H,H_ = 14.2 Hz, ^3^*J*_H,H_ = 9.3 Hz, ^3^*J*_H,H_ = 5.9 Hz,CHSO_2_), 1.99–1.47 (m, 4 H, CH_2_), 1.35 (s, 9 H, CMe_3_); ^13^C NMR (75.5 MHz, 3:7 CDCl_3_-CD_3_OD) δ 160.0, 157.6 (CO), 80.5 (CMe_3_), 71.0 (C-3), 70.5 (C-4), 67.5 (C-2), 64.5 (C-6), 54.3 (C-5), 54.1 (C-1), 53.2, 52.9 (CH_2_SO_2_CH_2_), 35.0 (CH_2_NHBoc), 28.6 (CMe_3_), 23.7, 19.5 (CH_2_); ESIMS: *m*/*z* 461.25 [M + Na]^+^; Anal. Calcd for C_17_H_30_N_2_O_9_S: C 46.57, H 6.90, N 6.39, S 7.31. Found: C 46.28, H 6.69, N 6.11, S 7.03.

### 3.6. General Procedure for Antiproliferative Assays

All reagents were used as purchased from commercial suppliers without further purification. The human solid tumor cell lines A549 (lung), HBL-100 (breast), HeLa (cervix), SW1573 (lung), T-47D (breast), and WiDr (colon) were used in this study. These cell lines were a kind gift from Prof. G. J. Peters (VU Medical Center, Amsterdam, The Netherlands).

#### Chemosensitive Testing

Cells were inoculated onto 96-well microtiter plates in a volume of 100 μL per well at densities of 2500 (A549, HBL-100, HeLa, and SW1573) and 5000 (T-47D and WiDr) cells per well, based on their doubling times. Compounds were initially dissolved in DMSO at 400 times the desired final maximum test concentration. Control cells were exposed to an equivalent concentration of DMSO (0.25% *v*/*v*, negative control). Each agent was tested in triplicate at different dilutions in the range of 1 to 100 μM. The drug treatment started on day 1 after plating. Drug incubation times were 48 h, after which cells were precipitated with 25 μL ice-cold TCA (50% *w*/*v*) and fixed for 60 min at 4 °C. Then the SRB assay was performed. The optical density (OD) of each well was measured at 530 nm, using BioTek’s PowerWave XS Absorbance Microplate Reader. Values were corrected for background OD from wells only containing medium. The antiproliferative activity for each compound, expressed as GI50 values, was calculated according to NCI formulas [69].

### 3.7. General Procedure for Antileishmanial Assays 

#### 3.7.1. Leishmania Culture Conditions

For the biological assays, stock solutions of the synthesized compounds in DMSO at 10 mM were prepared. Triton X-100, paraformaldehyde, 3-(4,5-dimethyltriazol-2-yl)-2,5-diphenyltetrazolium bromide (MTT), resazurin and phorbol 12-myristate 13-acetate (PMA), were purchased from Sigma-Aldrich (St. Louis, MO, USA). Amphotericine was purchased from Zentaris GmbH (Frankfurt am Main, Germany). DMNPE-luciferin {D-luciferin-1[-(4,5-dimethoxy-2-nitrophenyl) ethyl ester]}, hygromycin B, and 4,6-diamidino-2-phenylindole dihydrochloride (DAPI) were purchased from Invitrogen (Carlsbad, CA, USA). Kit Luciferase Assay System was purchased from Promega. L-glutamine and penicillin/streptomycin were obtained from Gibco.

#### 3.7.2. Cell lines Culture and Determination of Cellular Toxicity 

Human myelomonocytic cell line THP-1 was grown at 37 °C and 5% CO_2_ in RPMI-1640 supplemented with 10% iFBS, 2 mM glutamate, 100 U/mL penicillin, and 100 μg/mL streptomycin; 3 × 10^4^ cells/well in 96-well plates were differentiated to macrophages with 20 ng/mL of PMA treatment for 48 h followed by 24 h of culture in fresh medium [70]. Cellular toxicity of all compounds was determined using the colorimetric MTT-based assay after incubation at 37 °C for 72 h in the presence of increasing concentrations of compounds [71]. The results are expressed as EC_50_ values, as the concentration of compound that reduce cell growth by 50% versus untreated control cells.

#### 3.7.3. Susceptibility Analysis in Intracellular Leishmania Amastigotes.

Macrophage-differentiated THP-1 cells, which are considered a suitable model for human macrophages, were plated at a density of 3 × 10^4^ or 3 × 10^5^ macrophages/well in 96-well white polystyrene microplates or 24-well tissue culture chamber slides, respectively, and were infected at a macrophage/parasite ratio of 1:10 with *L. donovani promastigotes*. Twenty-four hours after infection at 35 °C and 5% CO_2_, extracellular parasites were removed by washing with serum-free medium. Infected cell cultures were then incubated at different compound concentrations in RPMI 1640 medium plus 10% iFBS at 37 °C with 5% CO_2_ for 72 h. To determine the susceptibility of *L. donovani* LUC amastigotes, infected macrophages maintained in 96-well plates were lysed and then luminescence intensity was measured as indicative of the intracellular parasite growth, using the Luciferase Assay System Kit (Promega, Madison, Wis.) according to the instructions of the supplier. To determine the susceptibility of *L. donovani* HU3 amastigotes, infected macrophages maintained in 24-well plates were fixed for 30 min at 4 °C with 2.5% paraformaldehyde phosphate-buffered saline (PBS; 1.2 mM KH_2_PO_4_, 8.1 mM Na_2_HPO_4_, 130 mM NaCl, and 2.6 mM KCl adjusted to pH 7) and permeabilized with 0.1% Triton X-100 in PBS for 30 min. Intracellular parasites and macrophages were detected by nuclear staining with ProLong^®^ Gold antifade reagent plus DAPI (Invitrogen). The percentage infection and mean number of amastigotes from infected macrophages were determined with 200 macrophages/well.

## Figures and Tables

**Table 1:** Glycosidase inhibitory activities (*K*_i_, μM) of the new ONJ (**1**–**6**, **15**) and OGJ (**7**–**12**, **24**) *C*-glycoside derivatives. Values represent the mean ± SD (three independent determinations). Inhibition was competitive in all cases.

Compound ^a^	α-Glcase (Yeast Maltase)	β-Glcase (Bovine Liver)
**15**	79 ± 8	n.i.
**1**	0.34 ± 0.02	400 ± 20
**2**	0.74 ± 0.03	54 ± 5
**3**	0.28 ± 0.02	151 ± 10
**4**	2.6 ± 0.3	172 ± 14
**5**	2.5 ± 0.2	85 ± 18
**6**	0.75 ± 0.02	342 ± 20
**7**	n.i.	n.i.
**8**	n.i.	53 ± 12
**9**	n.i.	n.i.
**10**	n.i.	422 ± 22
**11**	n.i.	134 ± 10
**12**	n.i.	770 ± 45
**24**	n.i.	n.i.

^a^ No inhibition was observed for any compound at 1 mM concentration on *Aspergillus niger* amyloglucosidase, almond β-glucosidase, green coffee bean α-galactosidase, E. *coli* β-galactosidase, Jack bean α-mannosidase, and *Helix pomatia* β-mannosidase.

**Table 2:** Drug susceptibility profile of the *C*-glycosides sp^2^-IGLs **1**–**12** for intracellular amastigotes of *L. donovani* (EC_50_ values, μM) and cellular toxicity (MTT assay) in THP-1 and MRC-5 cells (EC_50_ values, μM). Data for the reference drug AmB are also shown ^a^.

Compound	Intracellular Amastigotes	THP-1 (SI)	MRC-5 (SI)
**1**	7.68 ± 0.29	130.96 ± 4.29 (17.06)	32.20 ± 1.03 (4.19)
**2**	>20	72.24 ± 4.18 (<3.61)	19.92 ± 2.76 (<1)
**3**	14.96 ± 1.28	>200	133.89 ± 6.02 (8.95)
**4**	>20	>200	188.98 ± 15.59 (<9.45)
**5**	11.44 ± 5.08	100.43 ± 10.87 (8.78)	71.49 ± 3.75 (6.25)
**6**	>20	>200	155.18 ± 3.10 (<7.76)
**7**	10.58 ± 1.51	127.55 ± 5.71 (12.06)	60.44 ± 2.80 (5.72)
**8**	>20	68.30 ± 3.04 (<3.42)	32.08 ± 1.94 (<1.60)
**9**	13.39 ± 1.13	>200	139.92 ± 17.31 (10.44)
**10**	13.81 ± 1.72	>200	184.97 ± 21.25 (13.40)
**11**	>20	>200	161.89 ± 20.18 (<8.10)
**12**	>20	>200	181.51 ± 14.93 (<9.08)
**AmB**	0.15 ± 0.01	20.07 ± 4.43 (133.80)	16.93 ± 2.44 (112.87)

^a^ Parasites were grown as described in the Experimental section for 72 h at 37 °C in the presence of increasing concentrations of compounds. THP-1 and MRC-5 cells were grown as described in the Experimental section for 72 h at 37 °C, in the presence of increasing concentrations of compounds. Cell viability was determined using the luciferase assay (intracellular amastigotes). AmB was used as the reference antileishmanial agent. Data are means of EC_50_ ± SD from three independent experiments. SI: Selectivity Index (EC_50_ THP-1 or MRC-5/EC_50_ parasite).

## Supplementary Materials

The following are available online at https://www.mdpi.com/1420-3049/24/16/2882/s1. Figures S1–S28: ^1^H and ^13^C NMR spectra of the new compounds **1–12, 15, 24**, Figures S29–S45: selected Dixon and Lineweaver–Burk plots for *K*i determinations and Figure S46: Antiproliferative activity (GI_50_) of new compounds.
