# Supplementary material for: Thiol-ene "Click" Synthesis and Pharmacological Evaluation of C-Glycoside sp2-Iminosugar Glycolipids
Source: Molecules. 2019 Aug 8;24(16):2882. doi: 10.3390/molecules24162882 (PMC6720825; doi:10.3390/molecules24162882)
Supplement: Supplementary file 1 [file molecules-24-02882-s001.pdf]

Thiol-ene "click" synthesis and pharmacological evaluation of C-glycoside sp^2-iminosugar glycolipids

Elena M. Sánchez-Fernández[1,*], M. Isabel García-Moreno[1], Raquel García-Hernández[2], José M. Padrón[3], José M. García Fernández[4], Francisco Gamarro[2], Carmen Ortiz Mellet[1,*]

Supplementary Information

List of contents

**S1 to S28**	NMR spectra of new *C*-glycoside sp^2-iminosugars.
**S29 to S45**	Dixon and Lineweaver-Burk plots for K_i determination
**S46**	Anti-proliferative activity (GI_{50}) of new *C*-glycoside sp^2-iminosugars

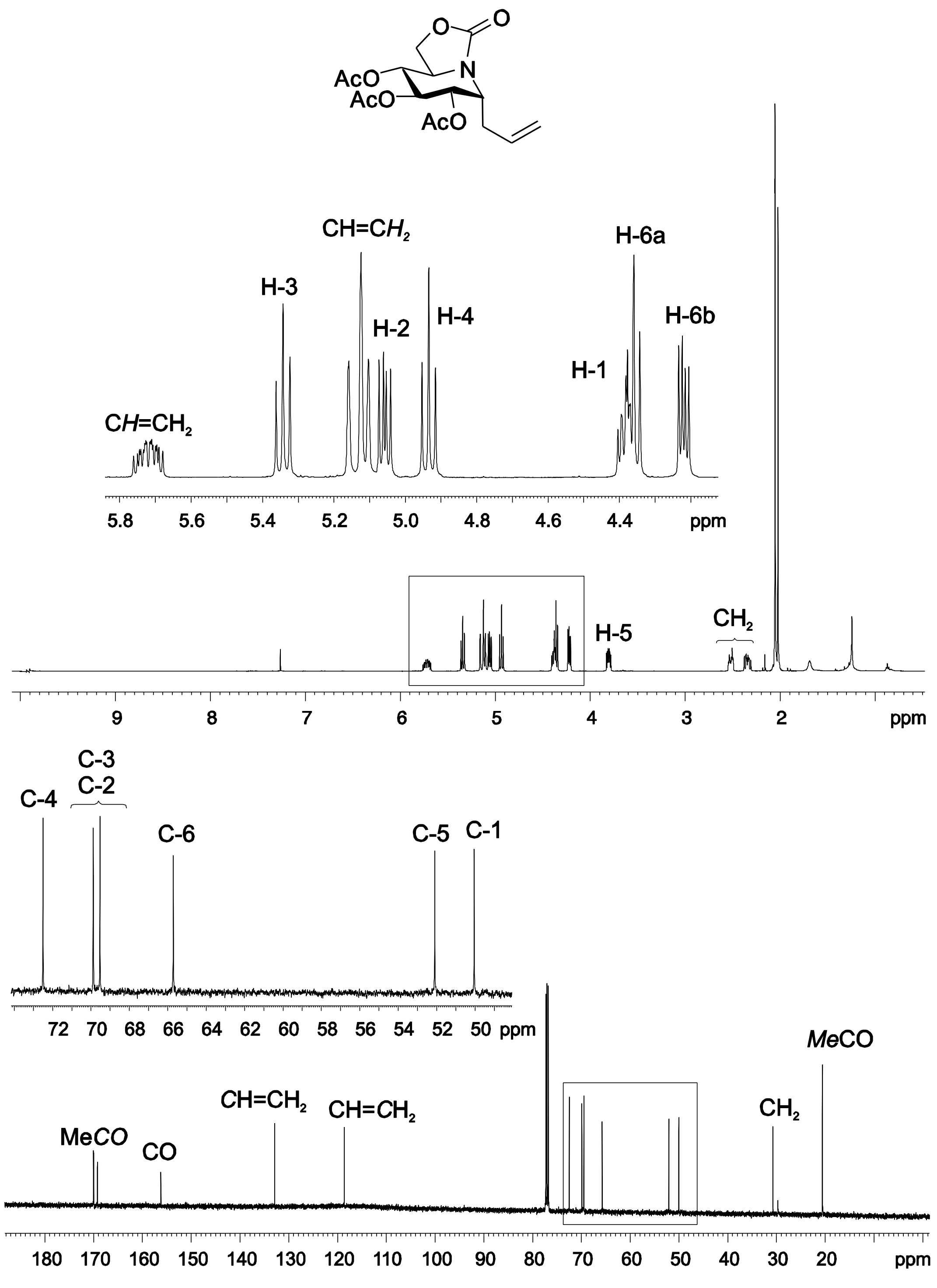

Figure S1. ^{1}H and ^{13}C NMR spectra (500 MHz and 125.7 MHz, $CDCl_3$) of **14**.

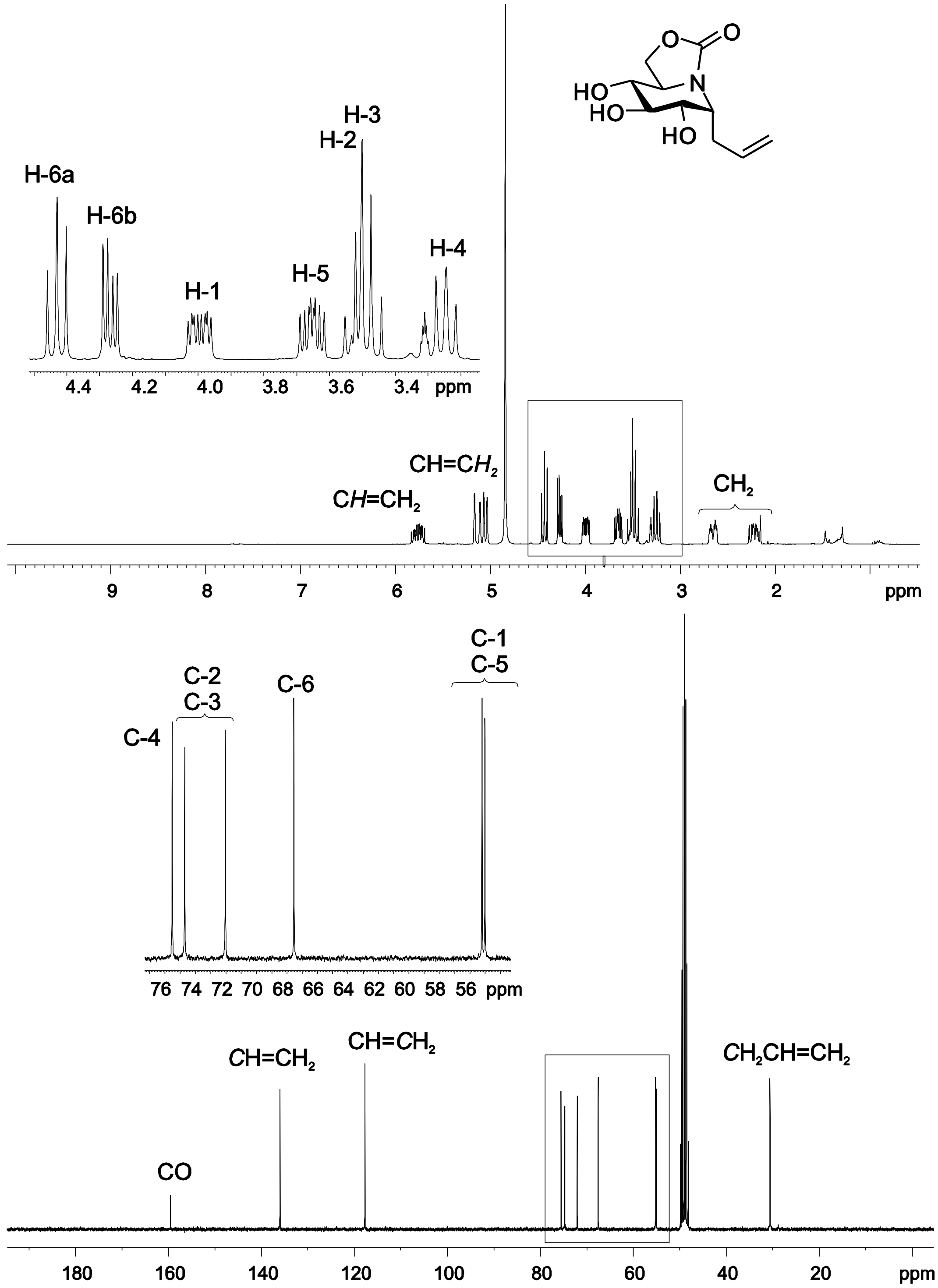

Figure S2. ^{1}H and ^{13}C NMR spectra (300 MHz and 75.5 MHz, CD_3OD) of **15**.

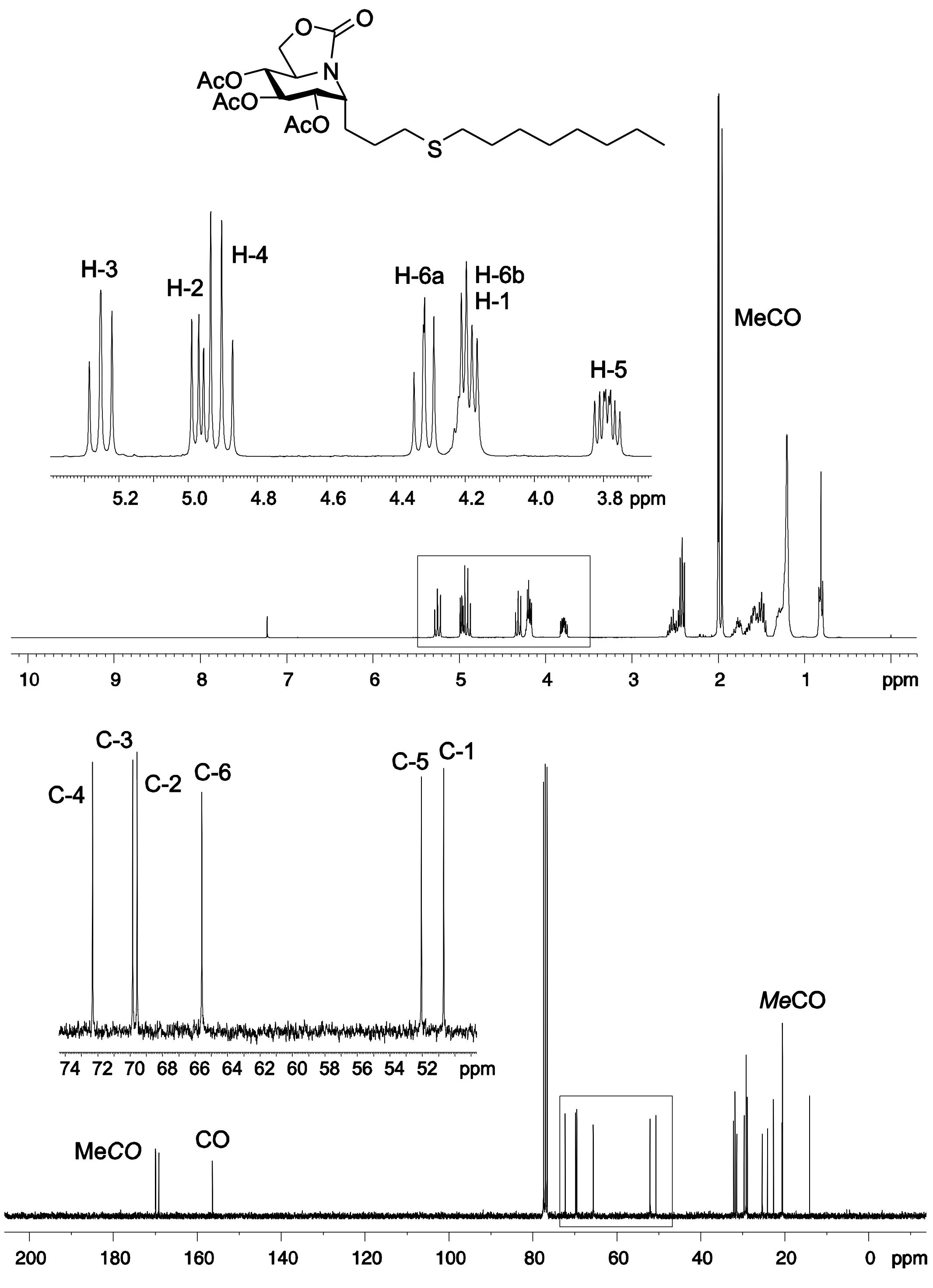

Figure S3. ^{1}H and ^{13}C NMR spectra (300 MHz and 75.5 MHz, $CDCl_3$) of **16**.

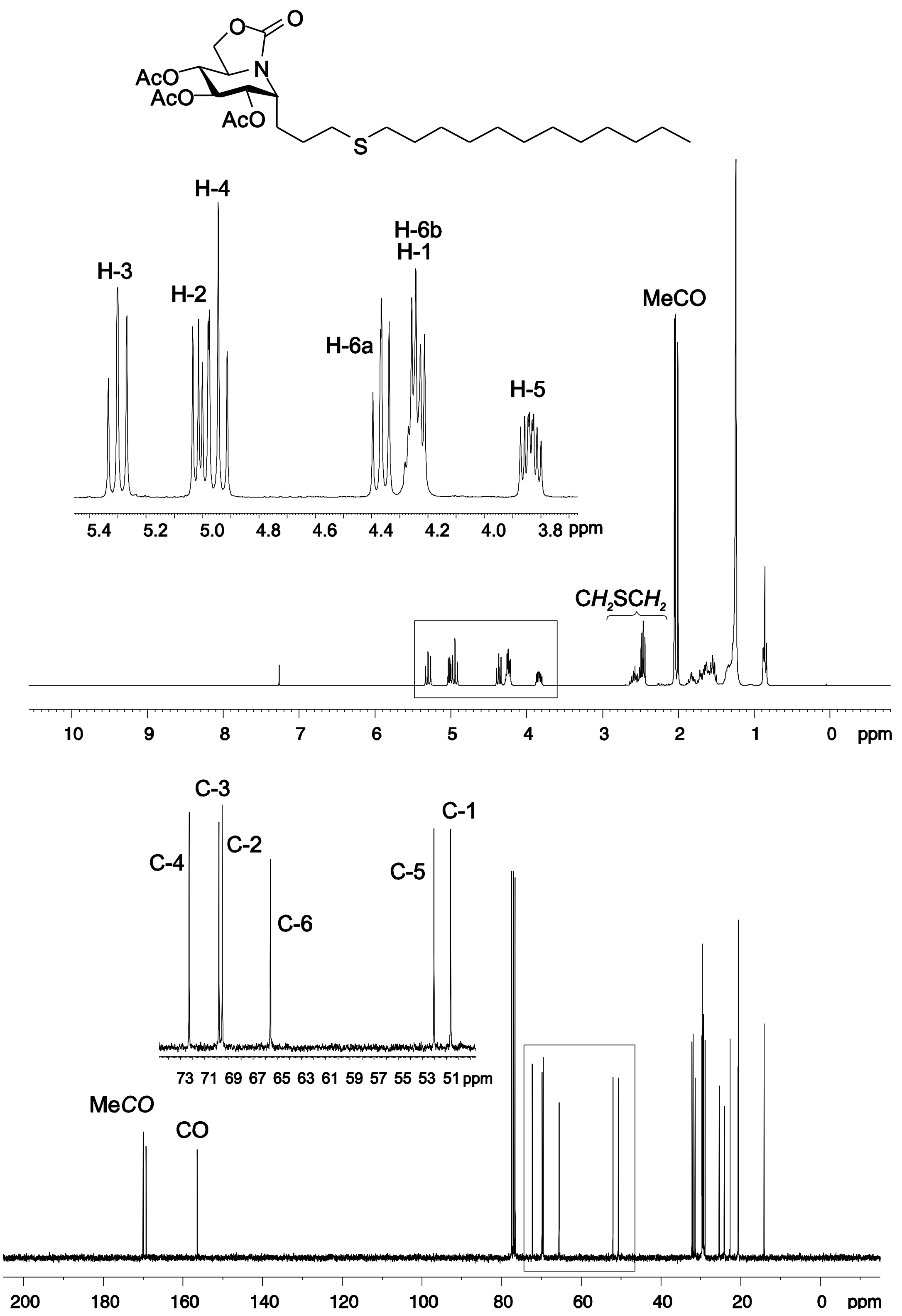

Figure S4. ^{1}H and ^{13}C NMR spectra (300 MHz and 75.5 MHz, $CDCl_3$) of **17**.

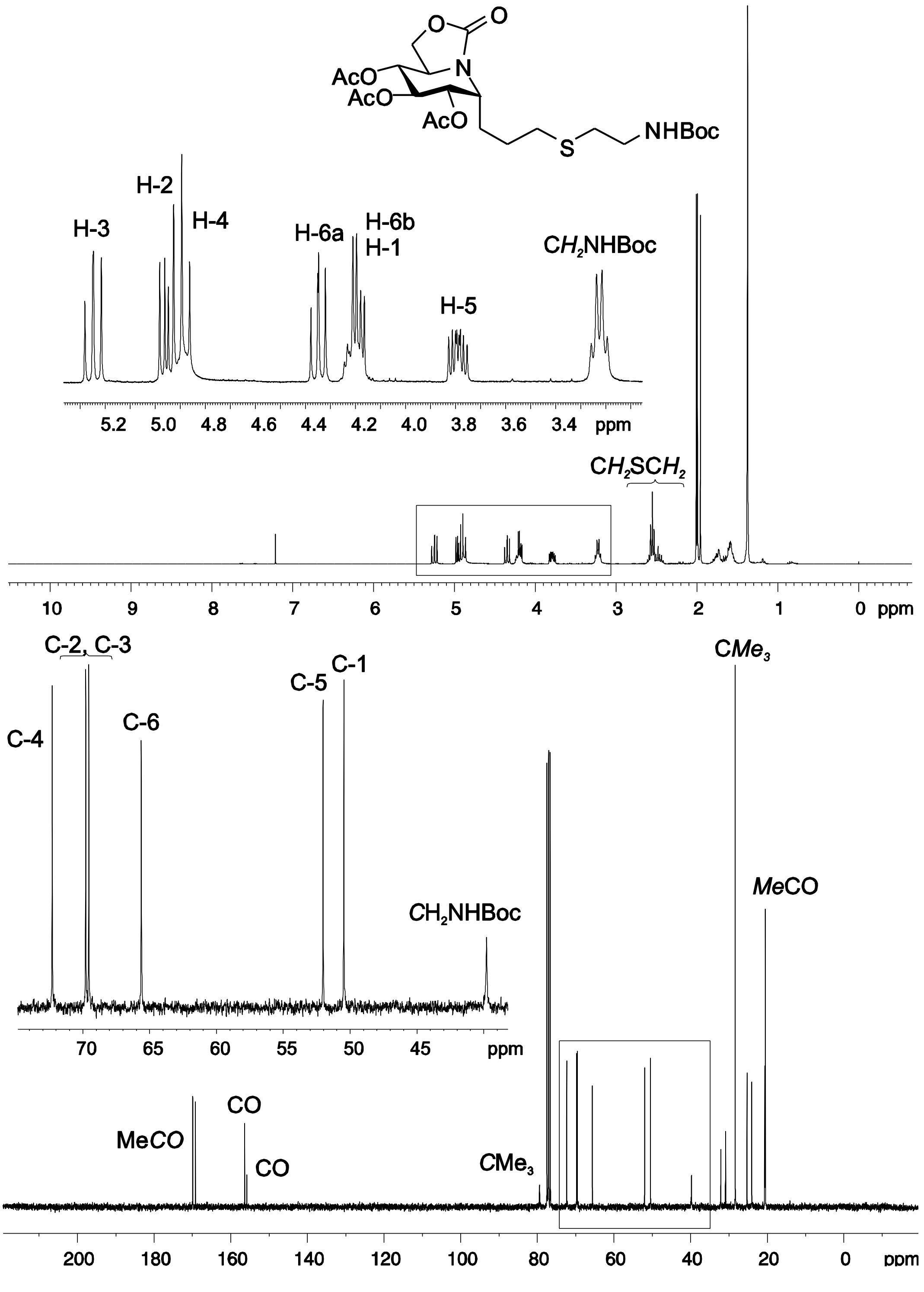

Figure S5. ^{1}H and ^{13}C NMR spectra (300 MHz and 75.5 MHz, $CDCl_3$) of **18**.

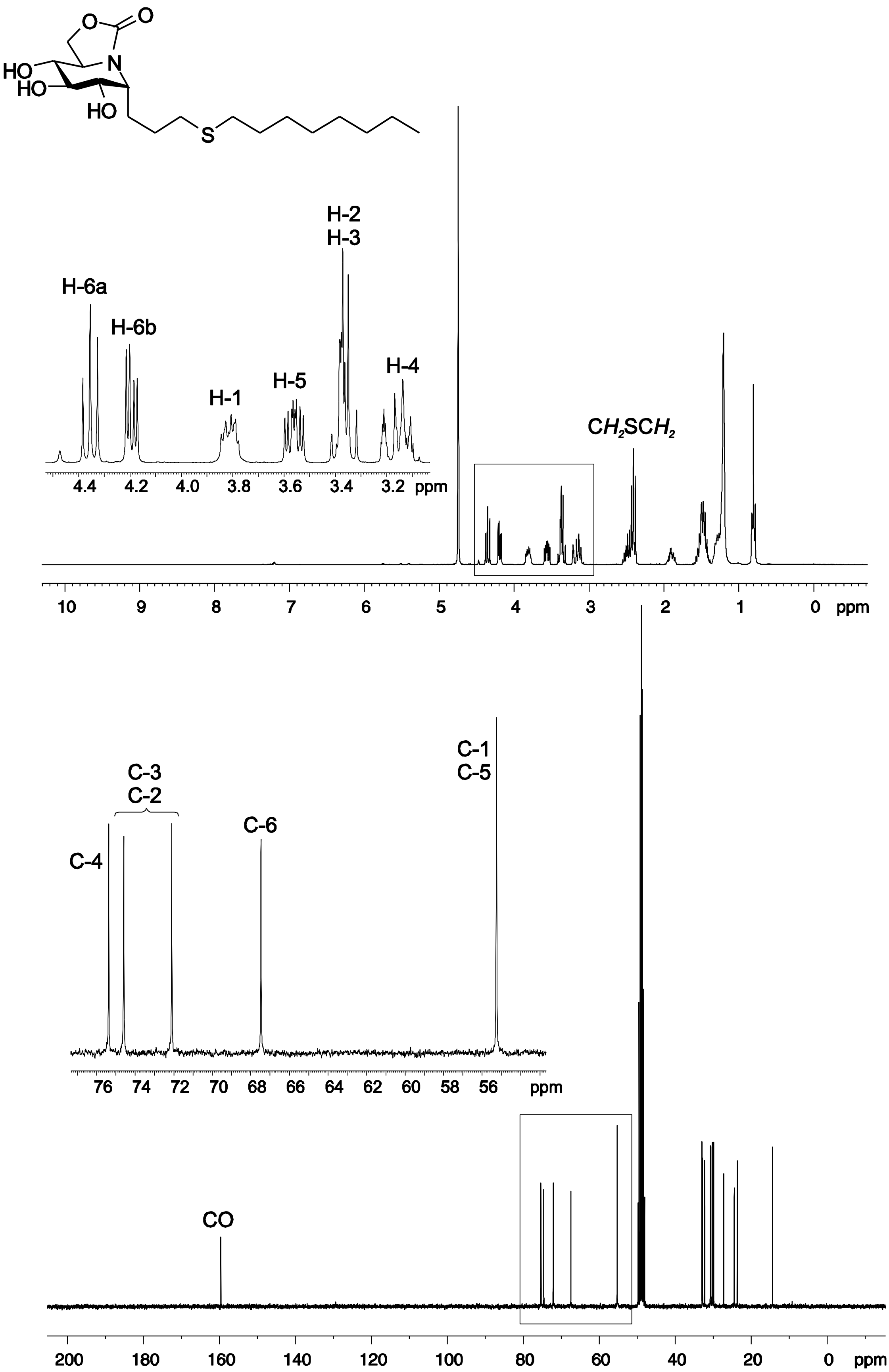

Figure S6. ^{1}H and ^{13}C NMR spectra (300 MHz and 75.5 MHz, CD_3OD) of **1**.

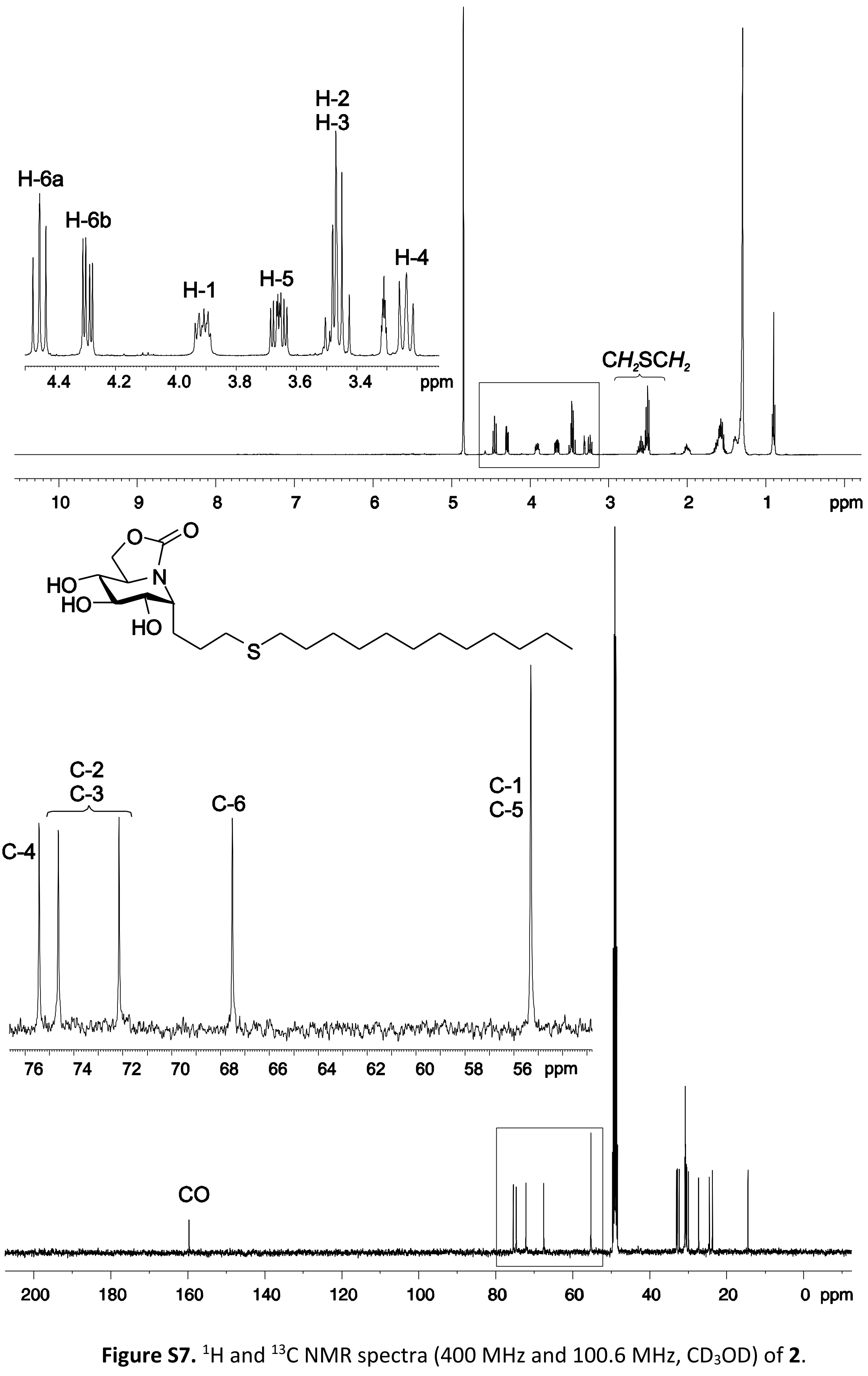

Figure S7. ^{1}H and ^{13}C NMR spectra (400 MHz and 100.6 MHz, CD_3OD) of **2**.

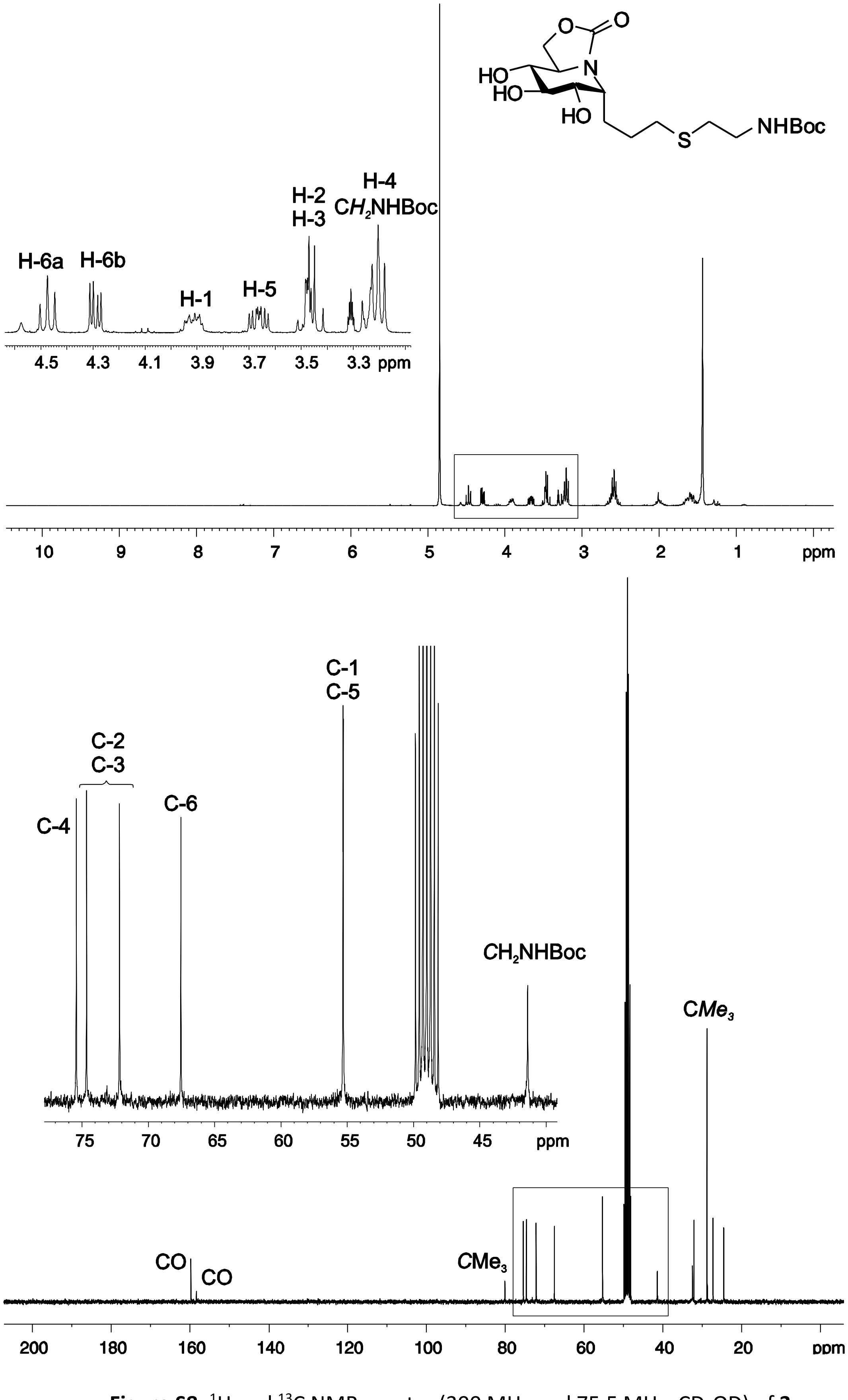

Figure S8. ^{1}H and ^{13}C NMR spectra (300 MHz and 75.5 MHz, CD_3OD) of **3**.

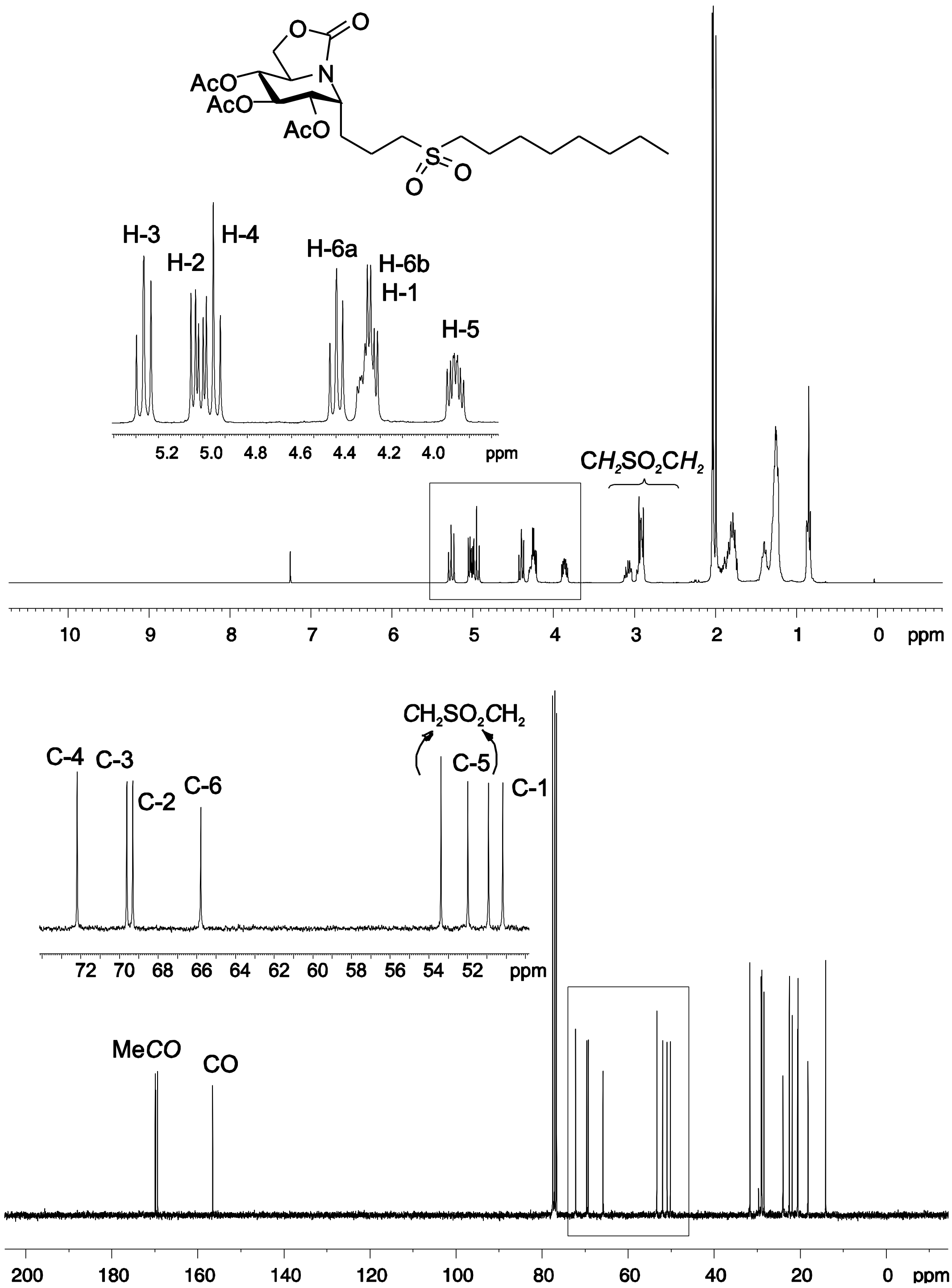

Figure S9. ^{1}H and ^{13}C NMR spectra (300 MHz and 75.5 MHz, $CDCl_3$) of **19**.

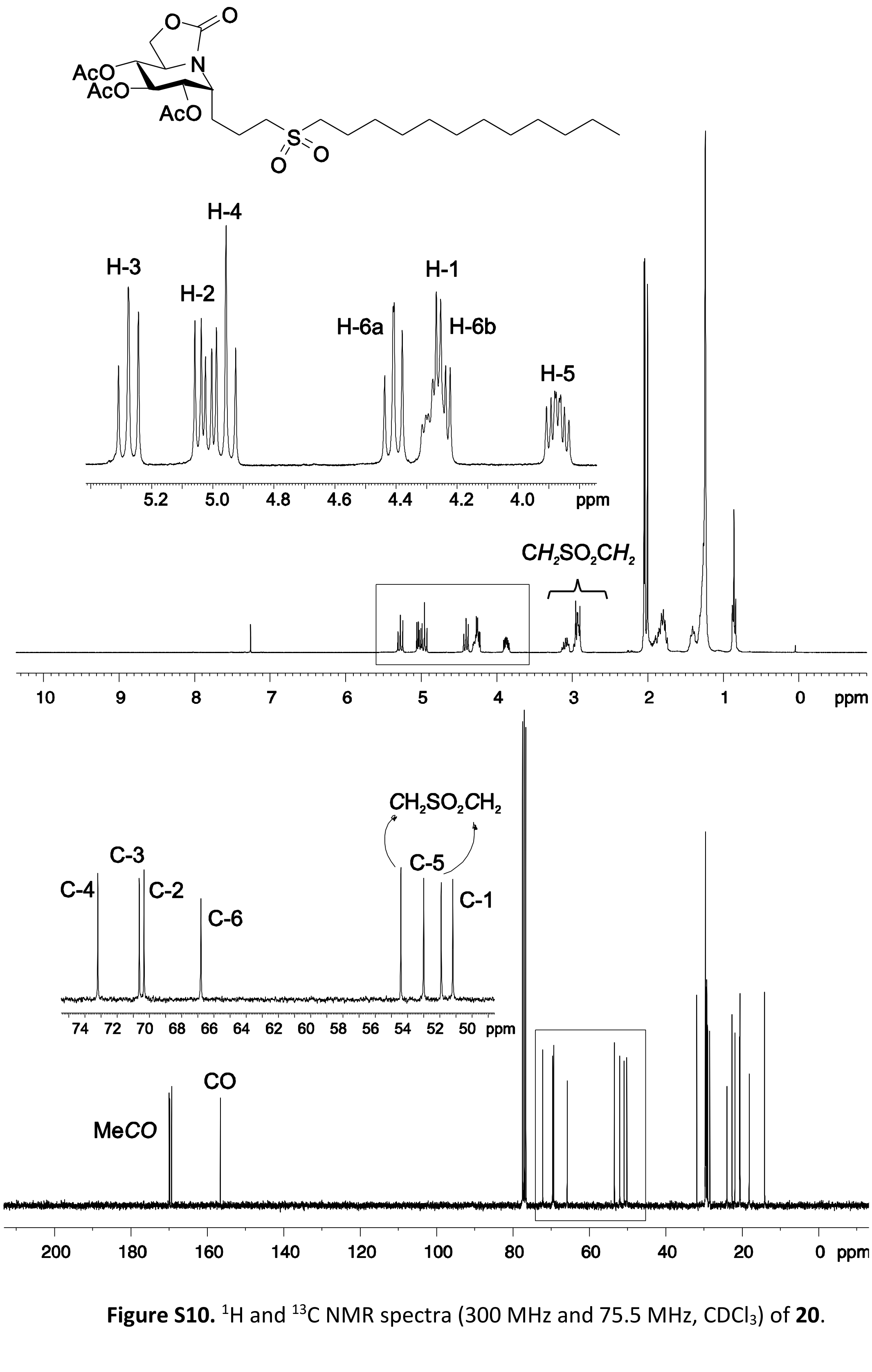

Figure S10. ^{1}H and ^{13}C NMR spectra (300 MHz and 75.5 MHz, $CDCl_3$) of **20**.

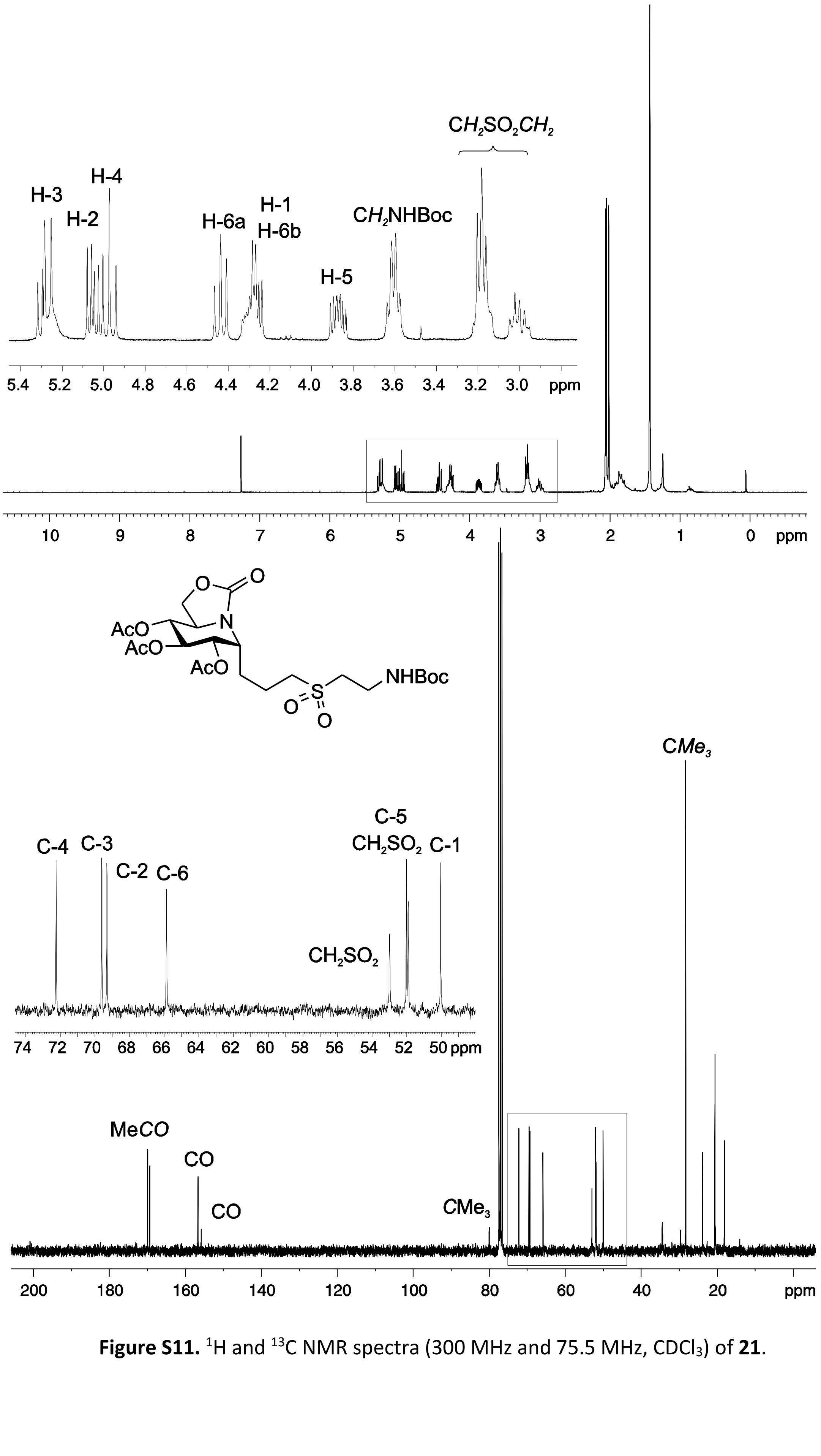

Figure S11. ^{1}H and ^{13}C NMR spectra (300 MHz and 75.5 MHz, $CDCl_3$) of **21**.

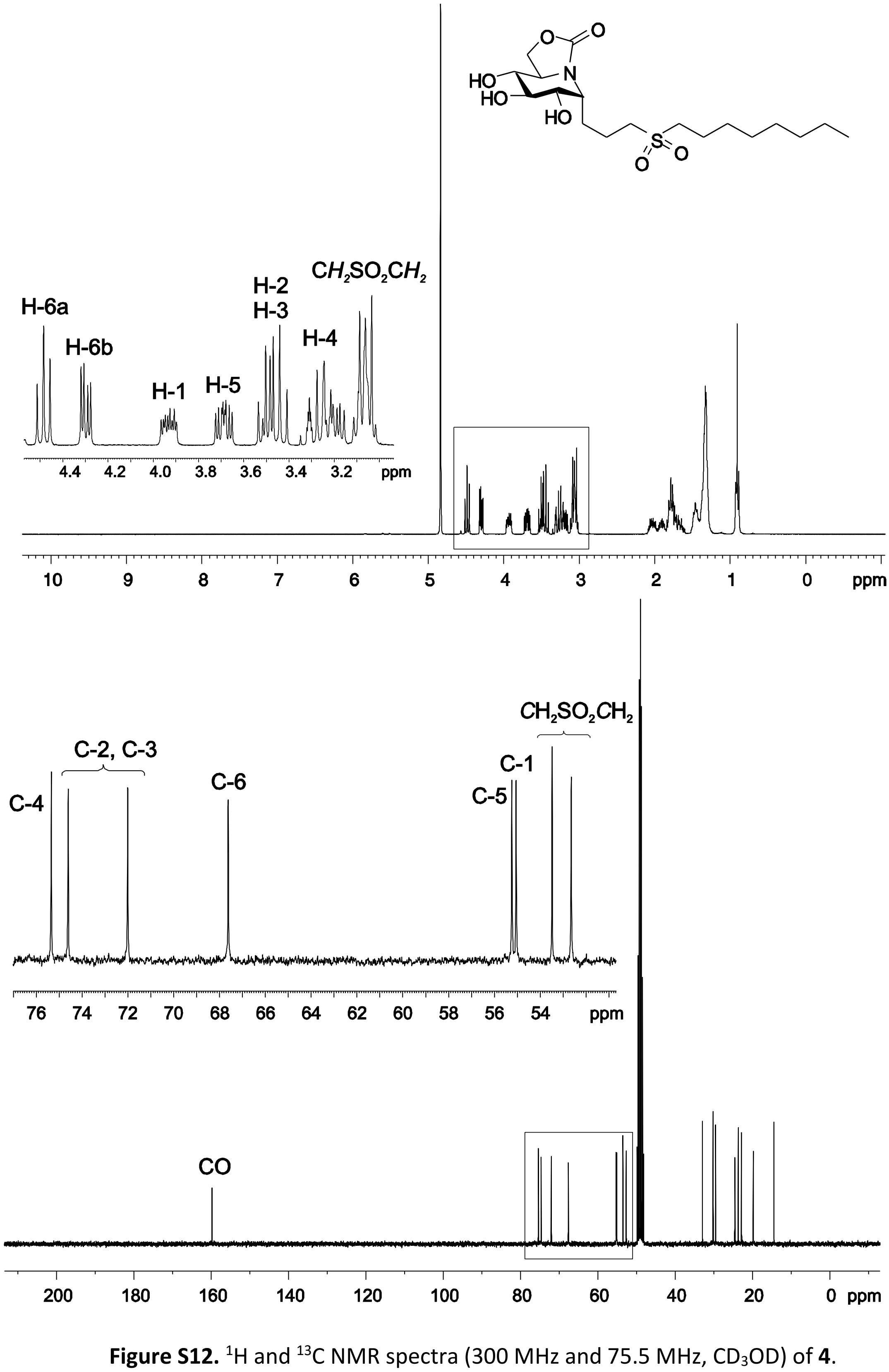

Figure S12. ^{1}H and ^{13}C NMR spectra (300 MHz and 75.5 MHz, CD_3OD) of **4**.

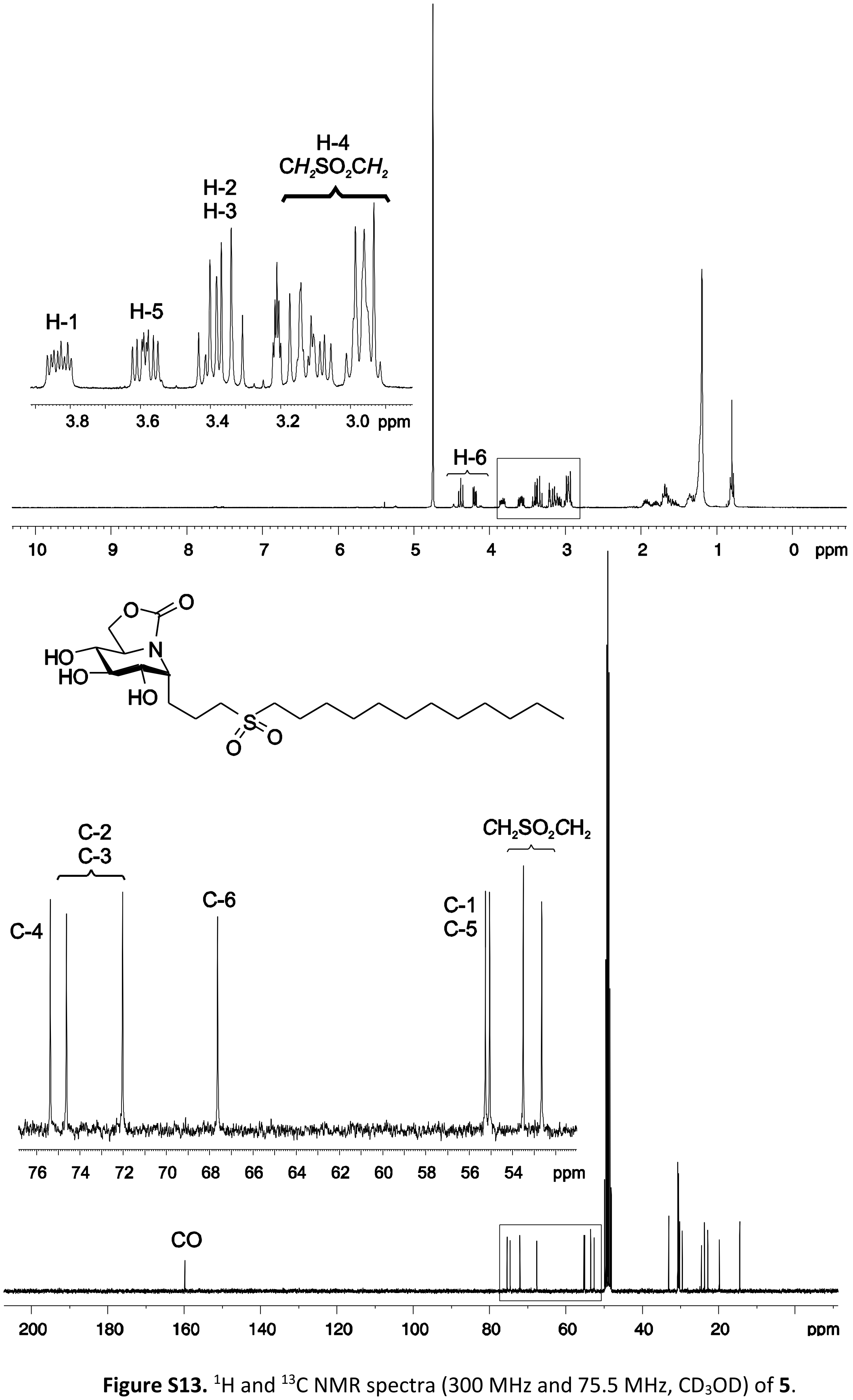

Figure S13. ^{1}H and ^{13}C NMR spectra (300 MHz and 75.5 MHz, CD_3OD) of **5**.

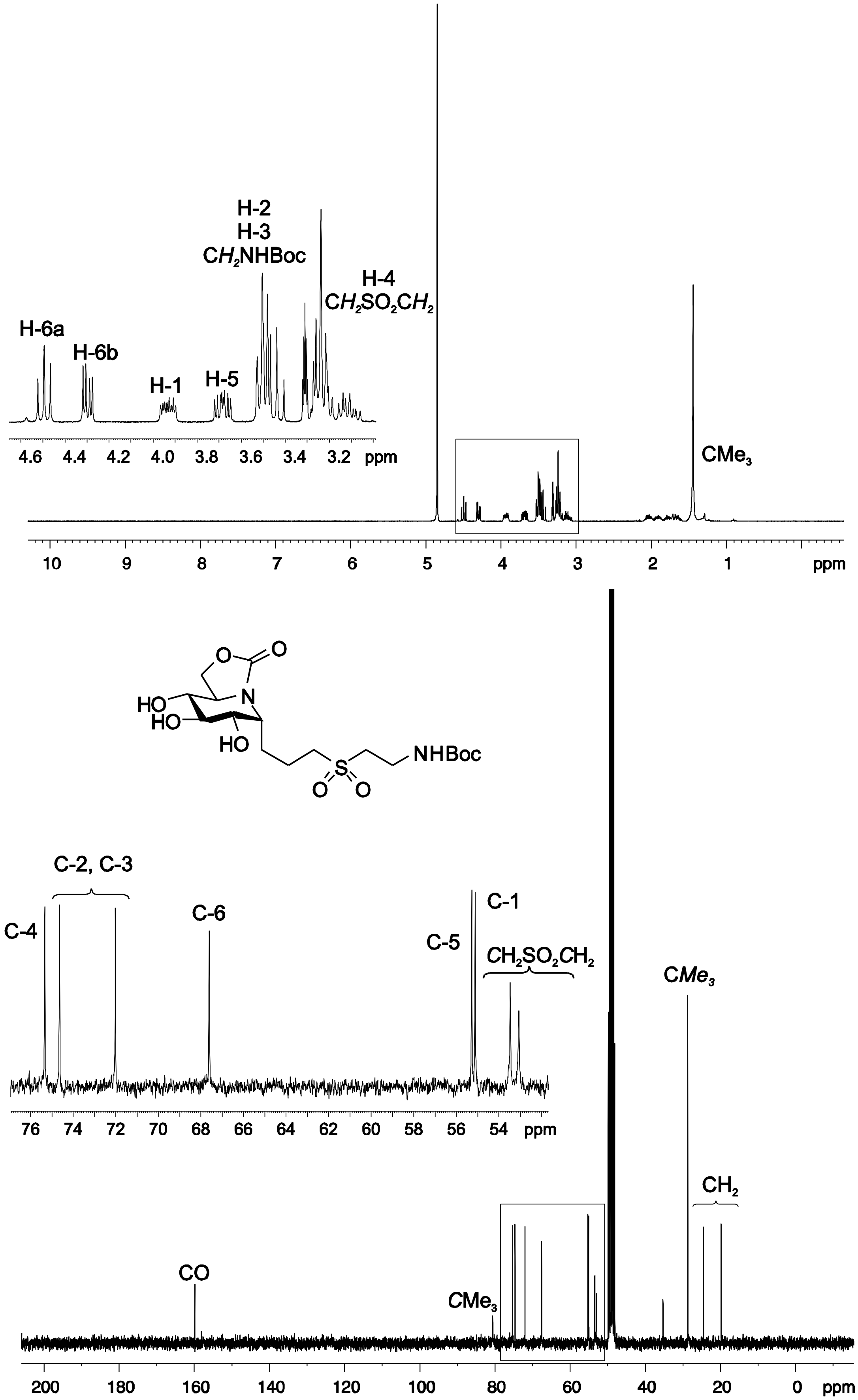

Figure S14. ^{1}H and ^{13}C NMR spectra (300 MHz and 75.5 MHz, CD_3OD) of **6**

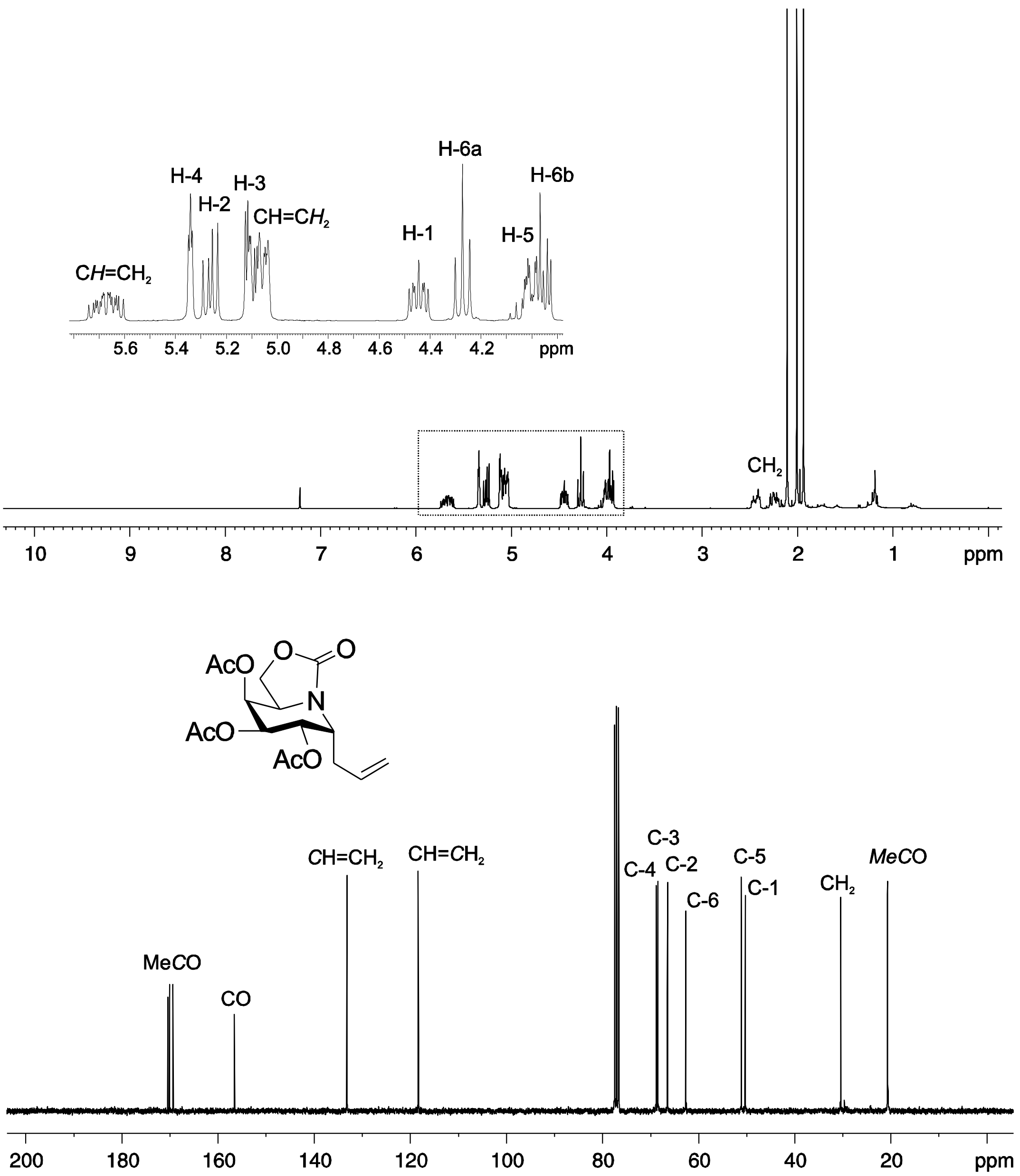

Figure S15. ^{1}H and ^{13}C NMR spectra (300 MHz and 75.5 MHz, $CDCl_3$) of **23**.

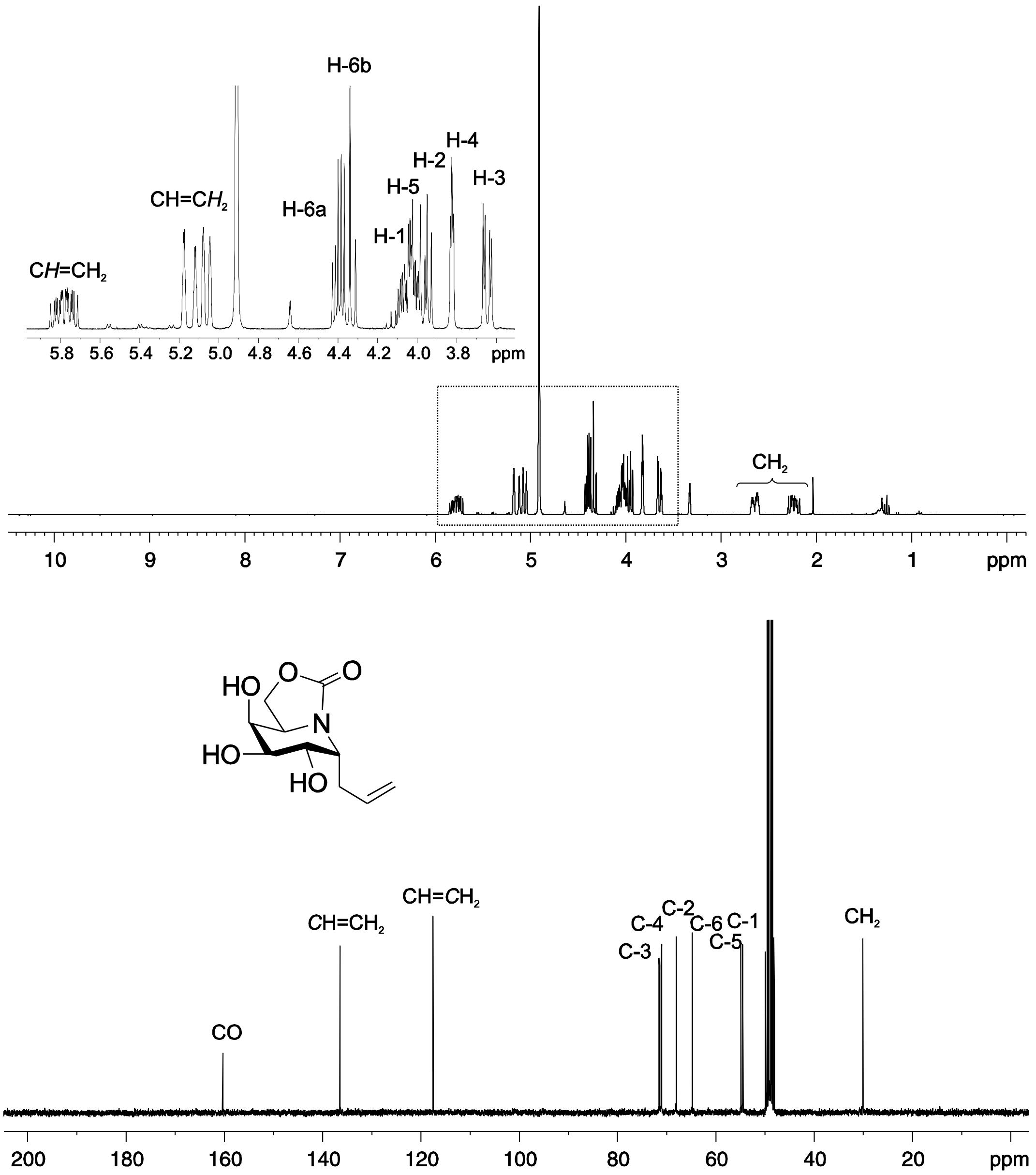

Figure S16. ^{1}H and ^{13}C NMR spectra (300 MHz and 75.5 MHz, CD_3OD) of **24**.

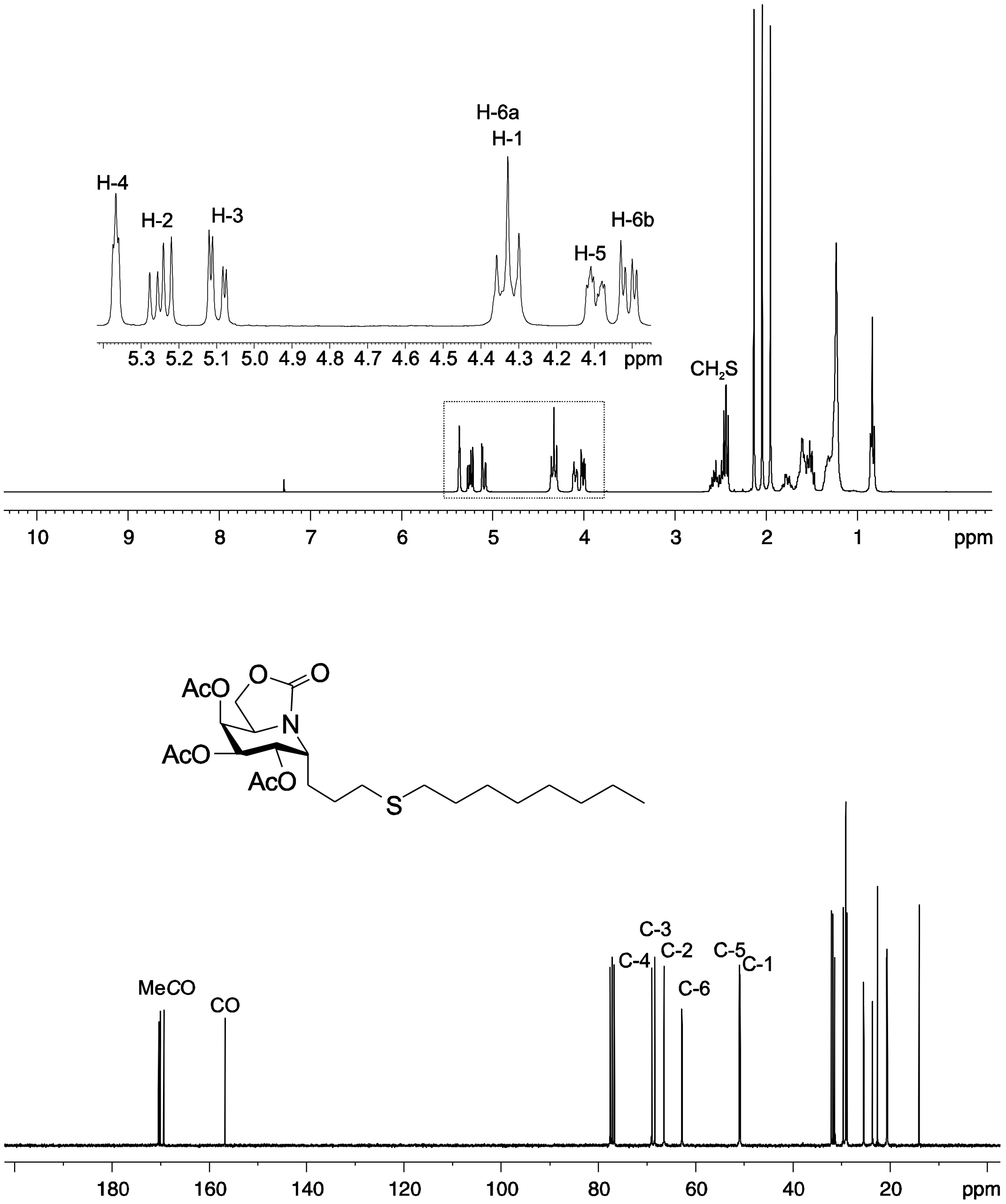

Figure S17. ^{1}H and ^{13}C NMR spectra (300 MHz and 75.5 MHz, $CDCl_3$) of **25**.

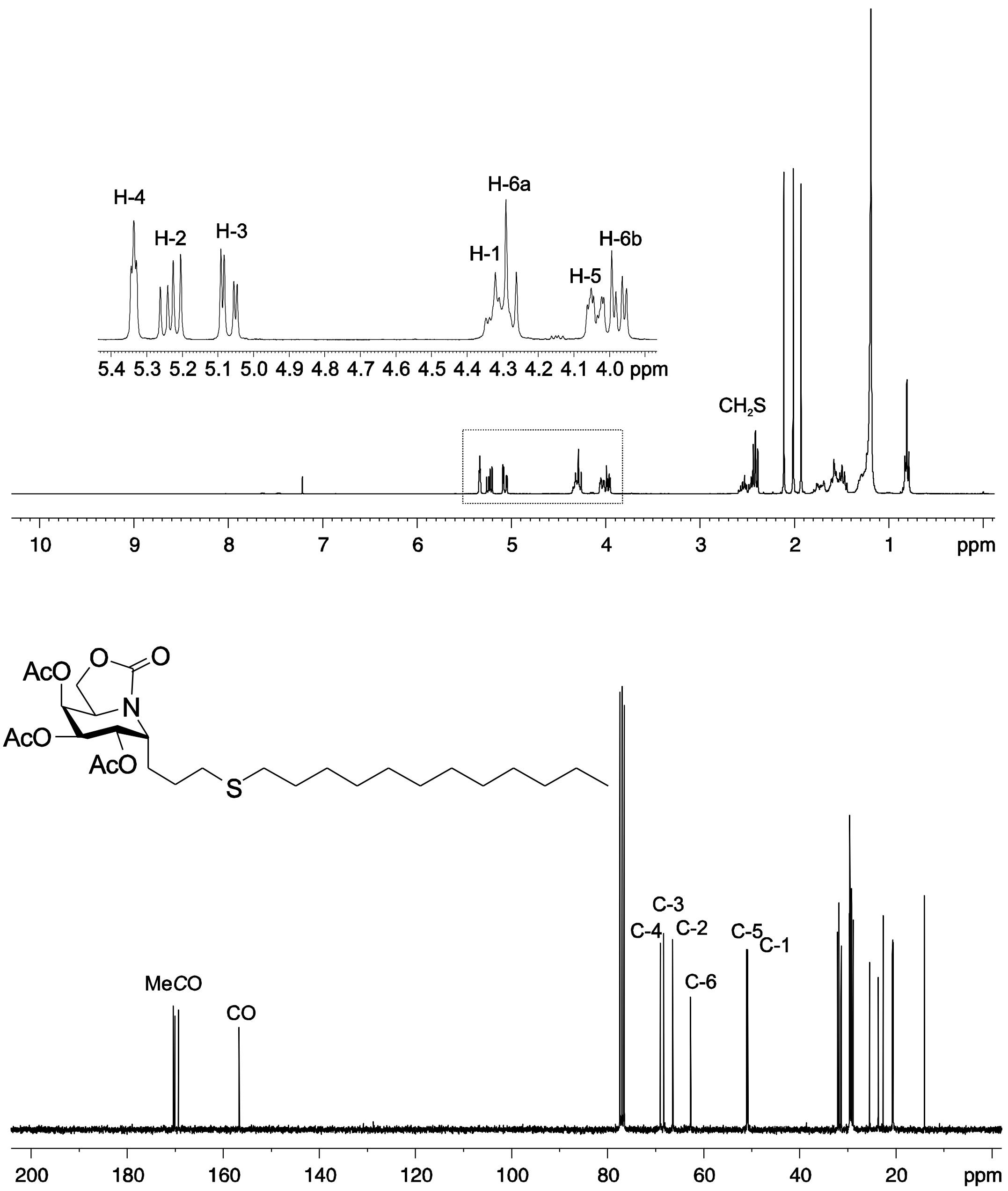

Figure S18. ^{1}H and ^{13}C NMR spectra (300 MHz and 75.5 MHz, $CDCl_3$) of **26**.

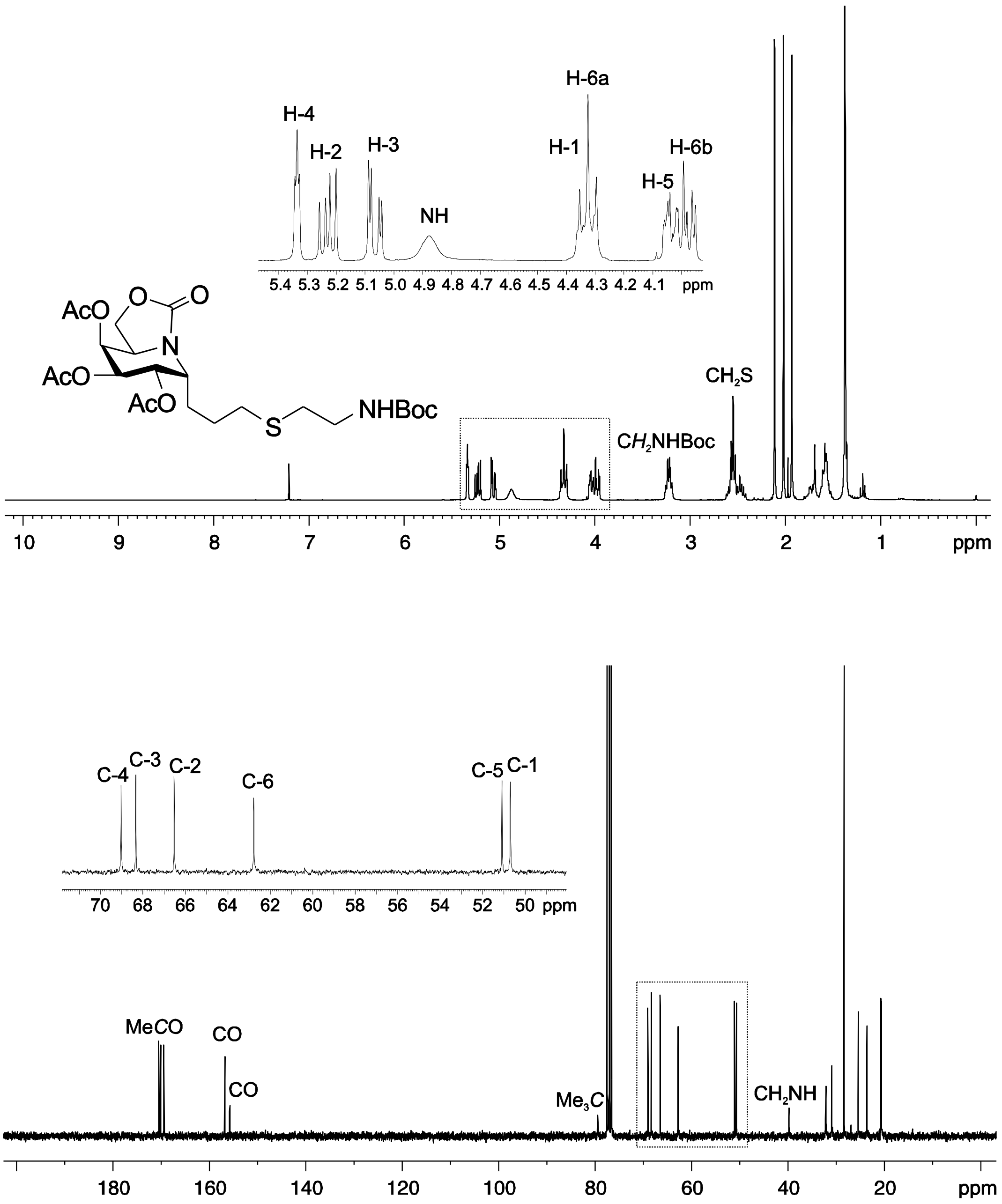

Figure S19. ^{1}H and ^{13}C NMR spectra (300 MHz and 75.5 MHz, $CDCl_3$) of **27**.

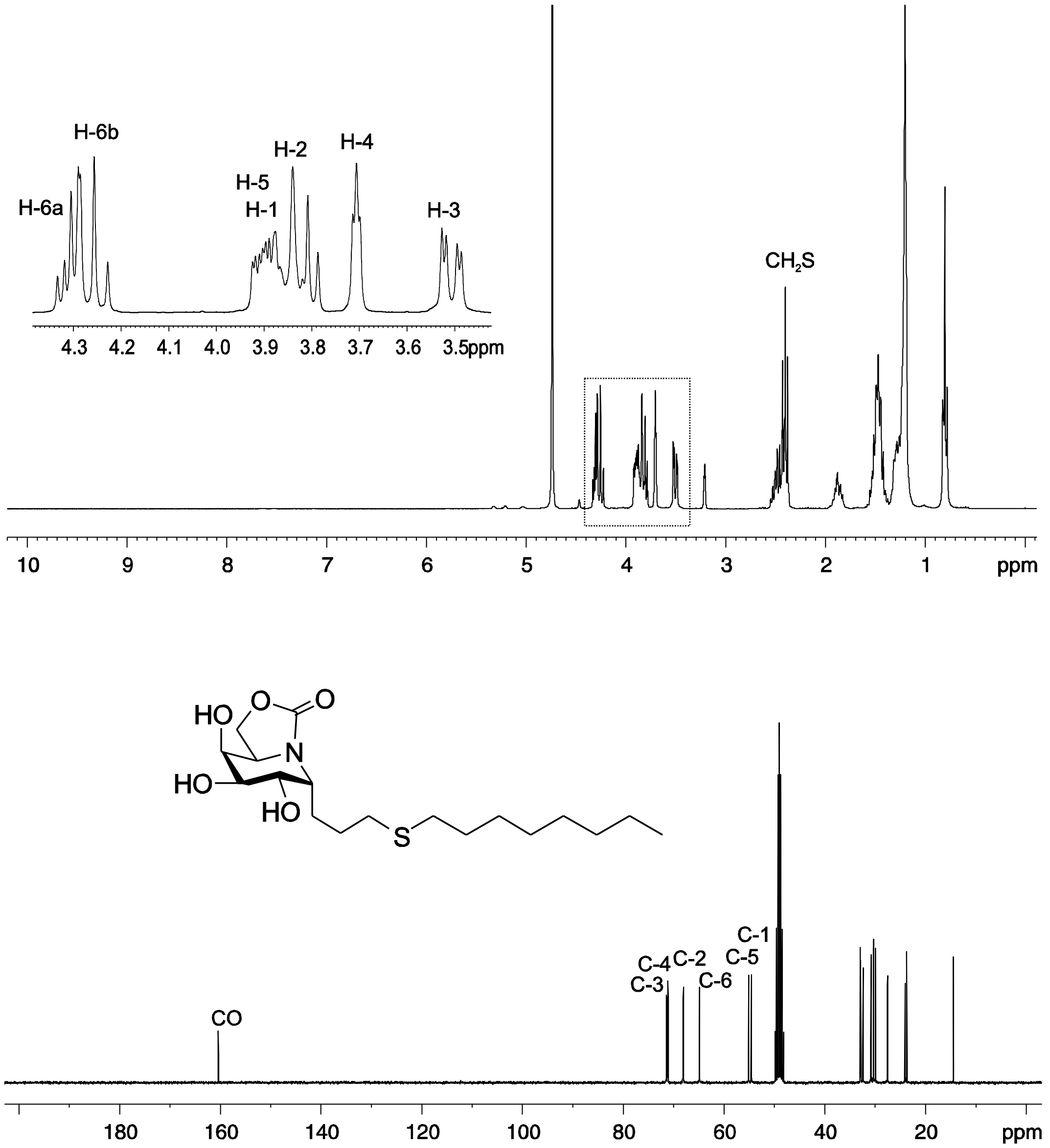

Figure S20. ^{1}H and ^{13}C NMR spectra (300 MHz and 75.5 MHz, CD_3OD) of **7**.

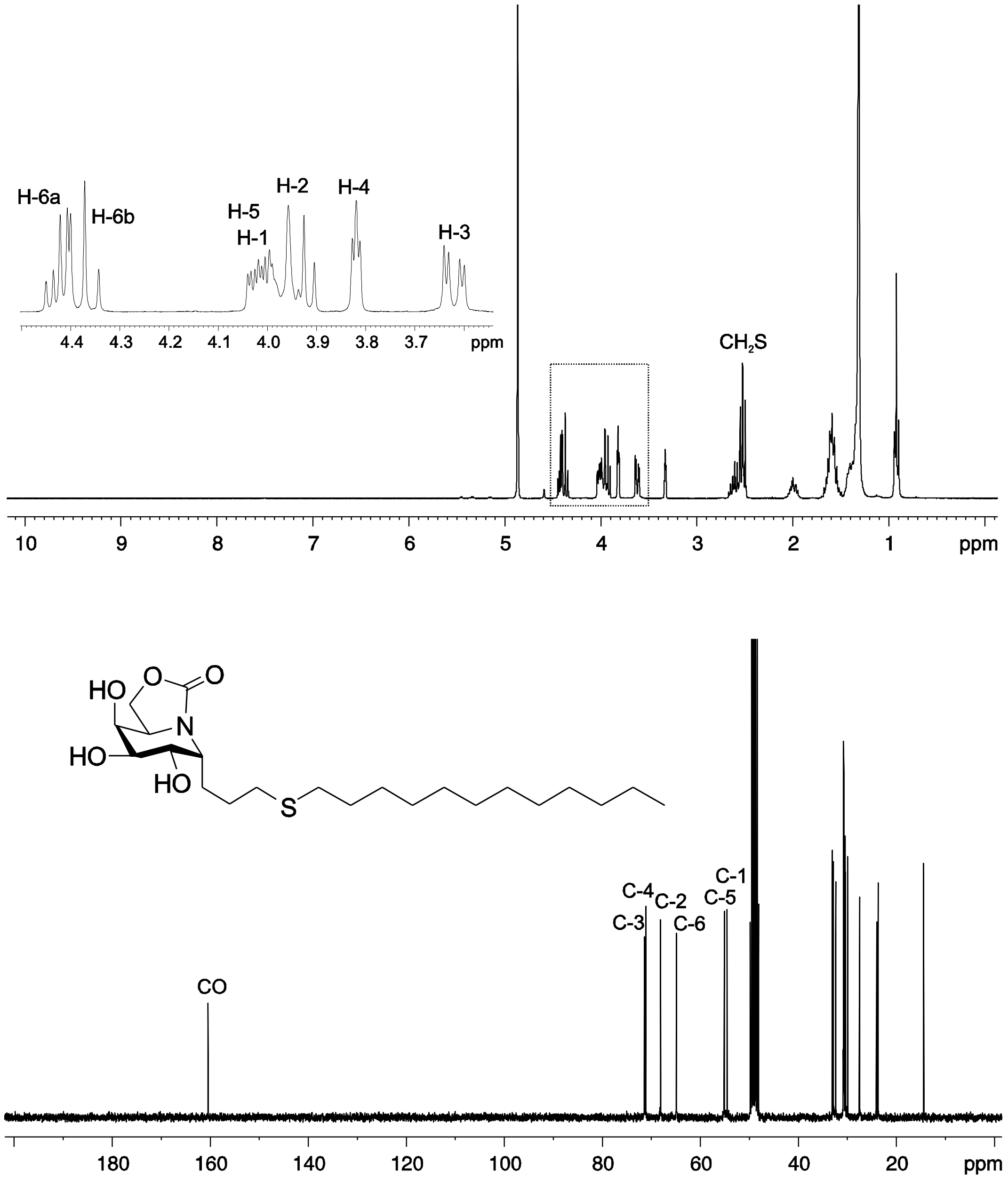

Figure S21. ^{1}H and ^{13}C NMR spectra (300 MHz and 75.5 MHz, CD_3OD) of **8**.

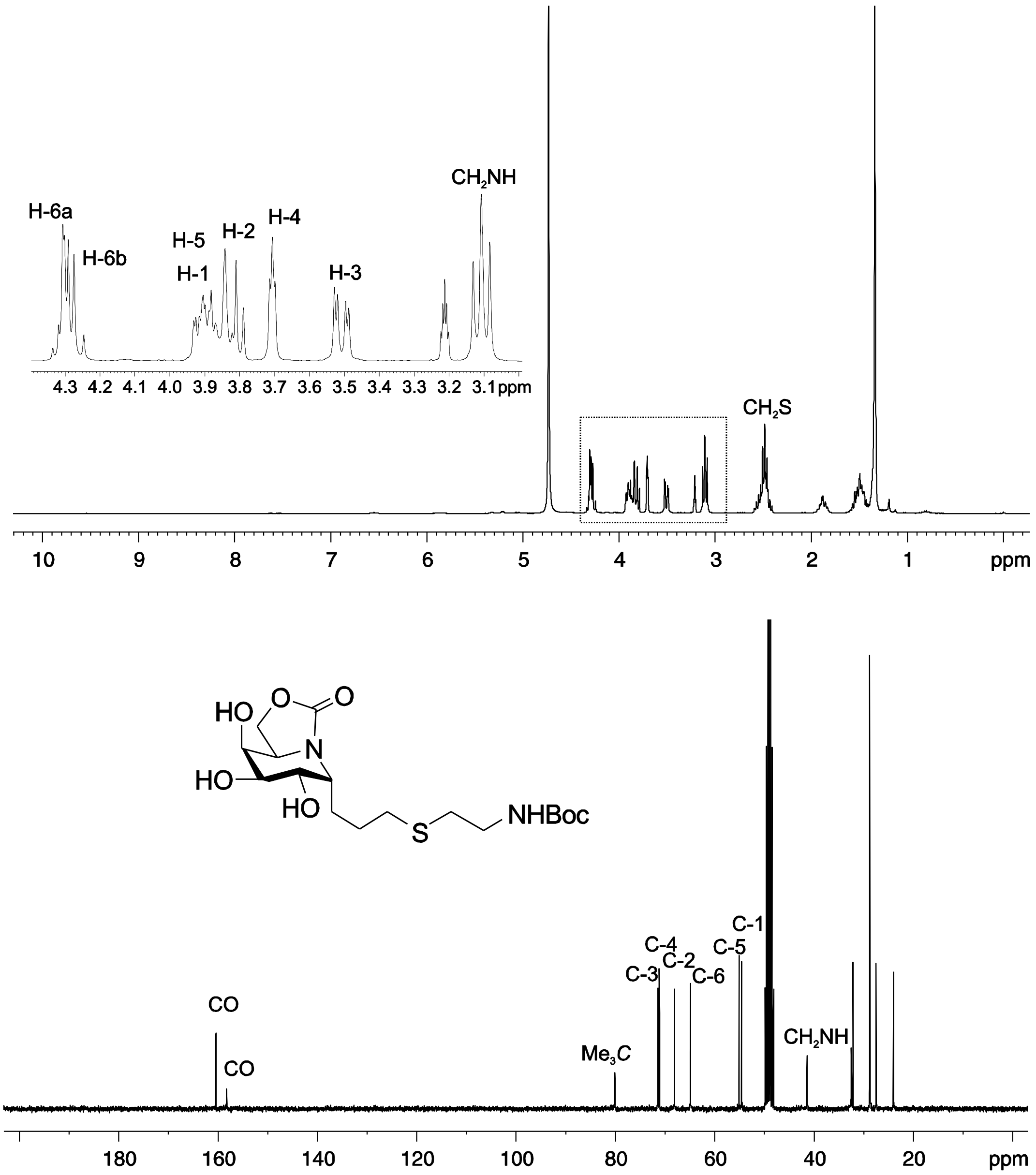

Figure S22. ^1H and ^{13}C NMR spectra (300 MHz and 75.5 MHz, CD_3OD) of **9**.

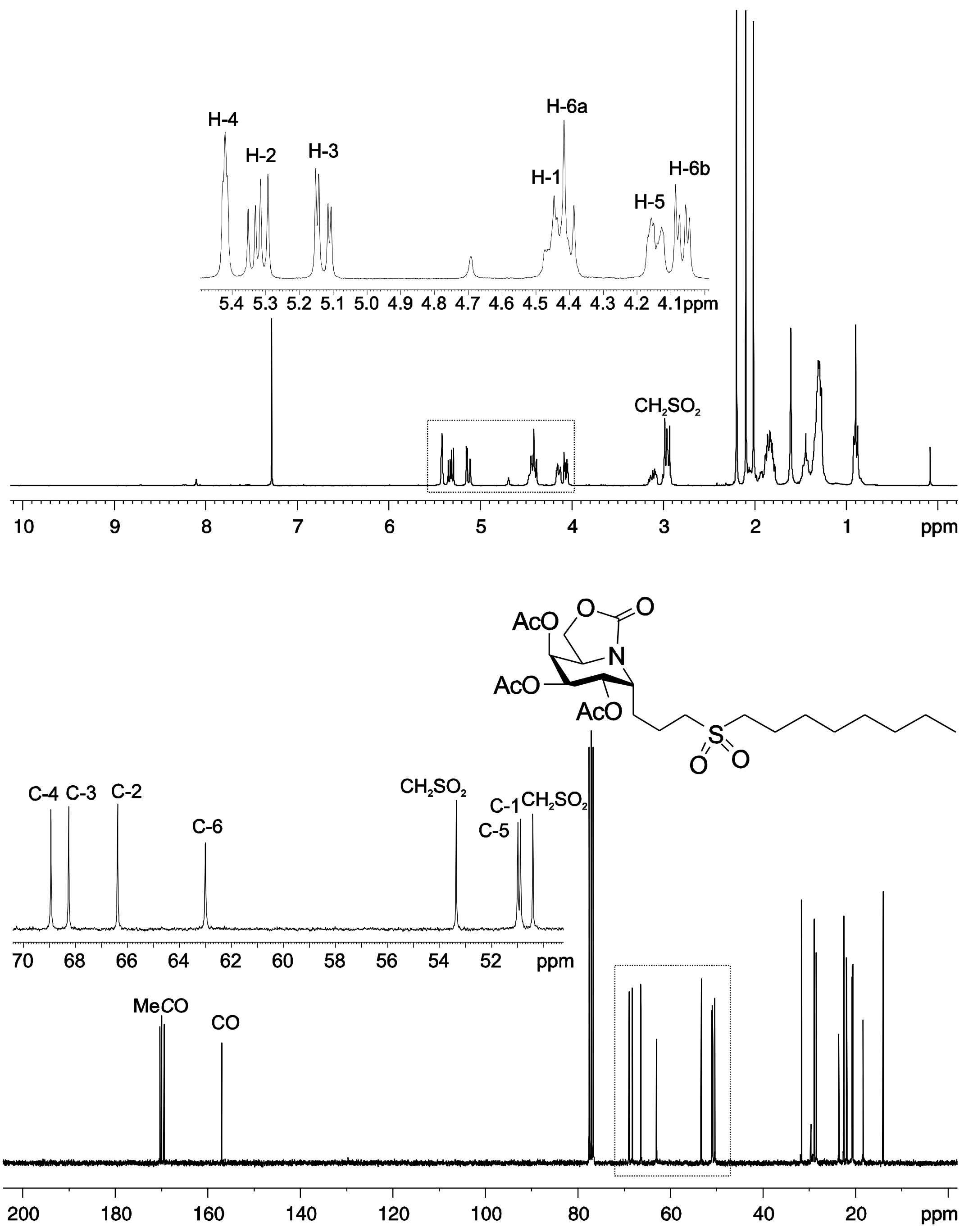

Figure S23. ^{1}H and ^{13}C NMR spectra (300 MHz and 75.5 MHz, $CDCl_3$) of **28**.

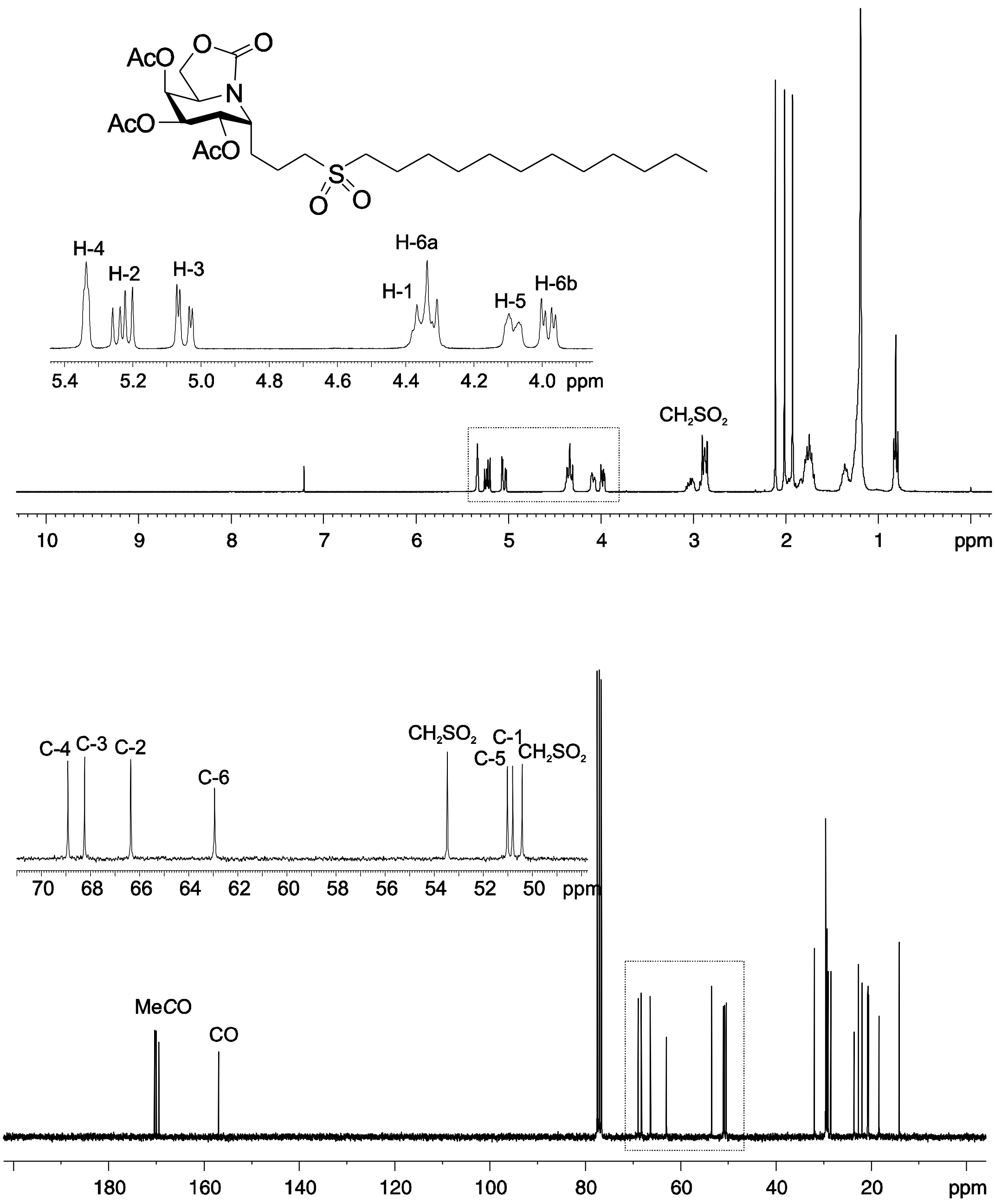

Figure S24. ^{1}H and ^{13}C NMR spectra (300 MHz and 75.5 MHz, CDCl$_3$) of **29**.

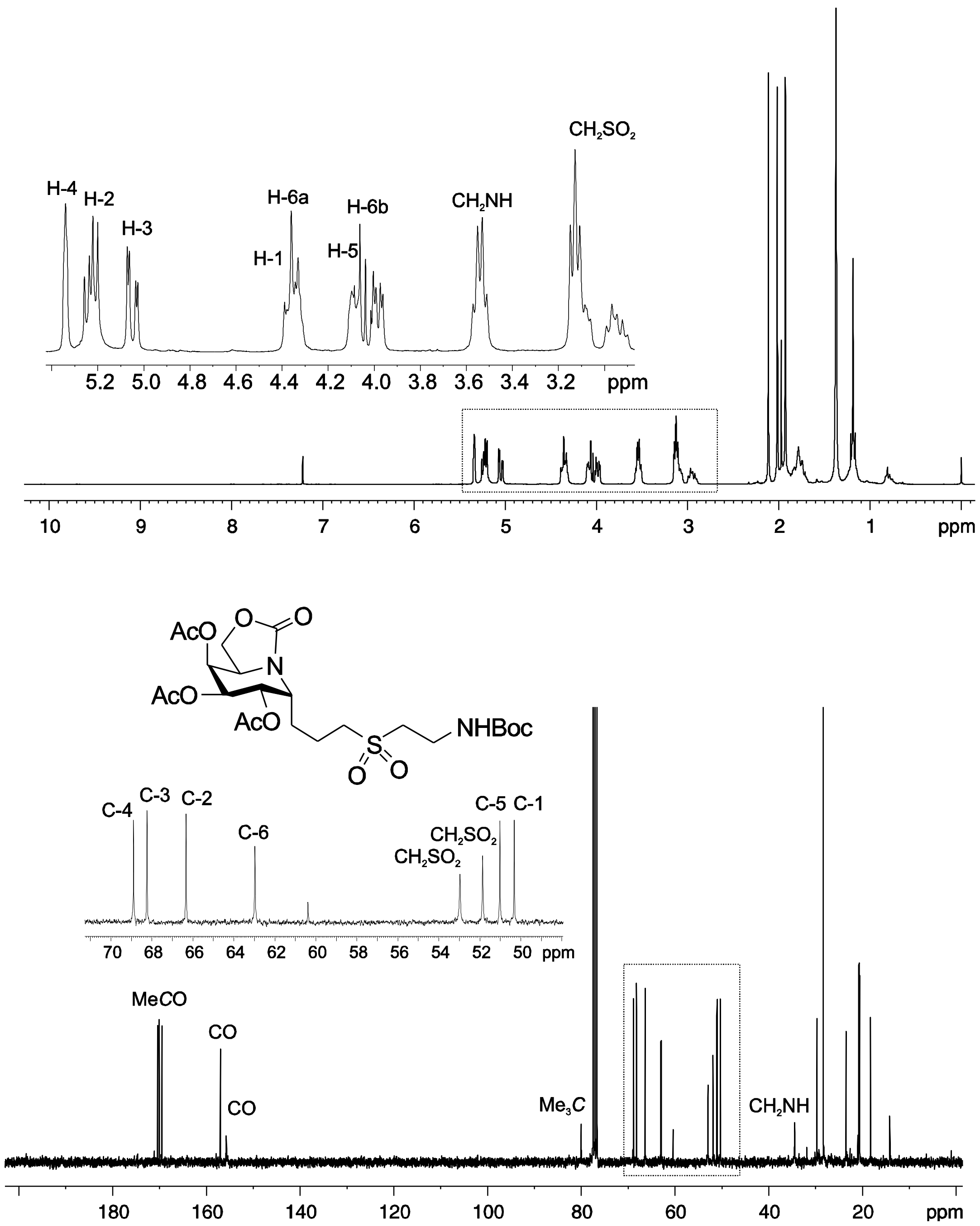

Figure S25. ^{1}H and ^{13}C NMR spectra (300 MHz and 75.5 MHz, $CDCl_3$) of **30**.

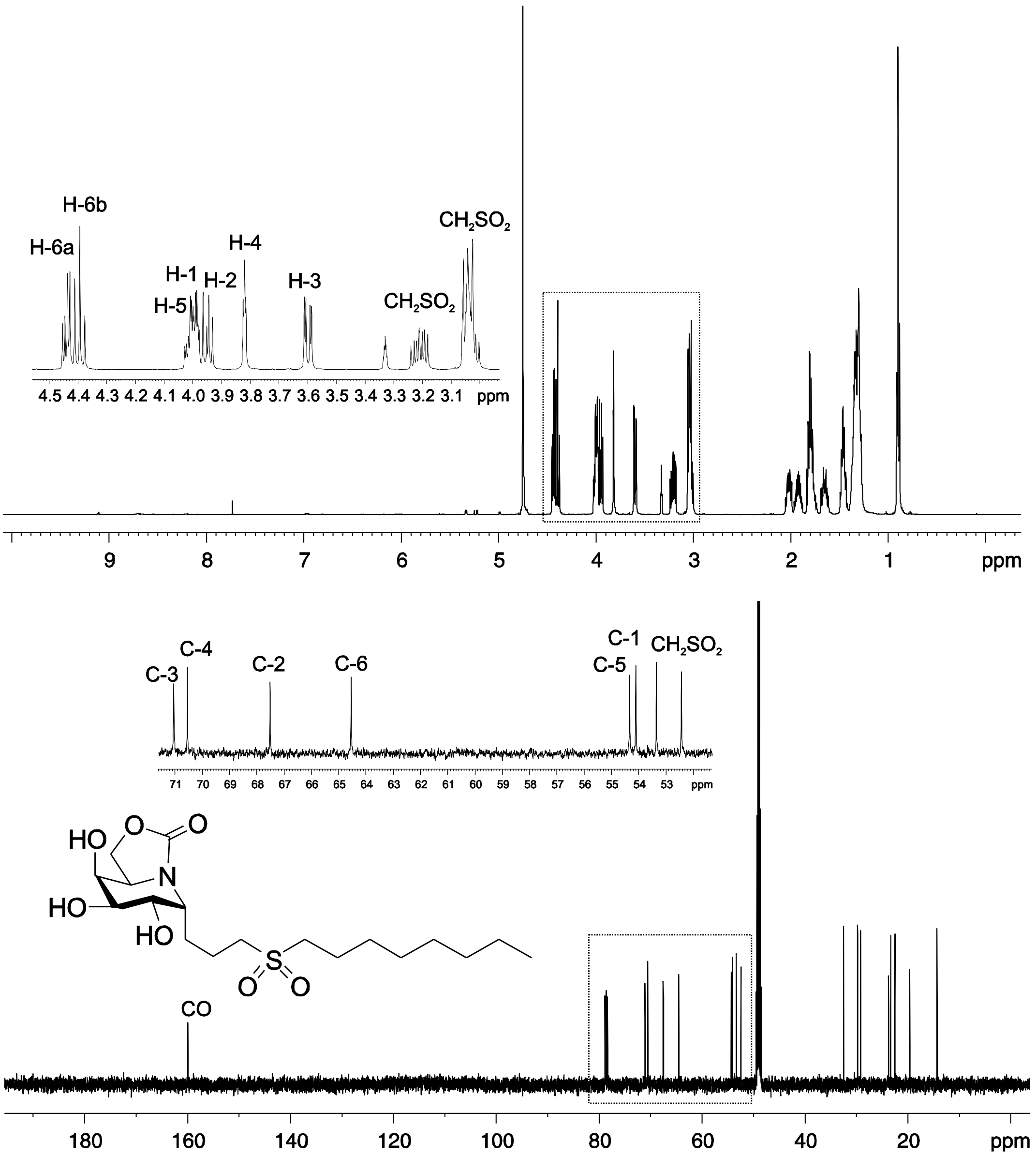

Figure S26. ^{1}H and ^{13}C NMR spectra (500 MHz and 125.7 MHz, 7:3 CD_3OD-$CDCl_3$) of **10**.

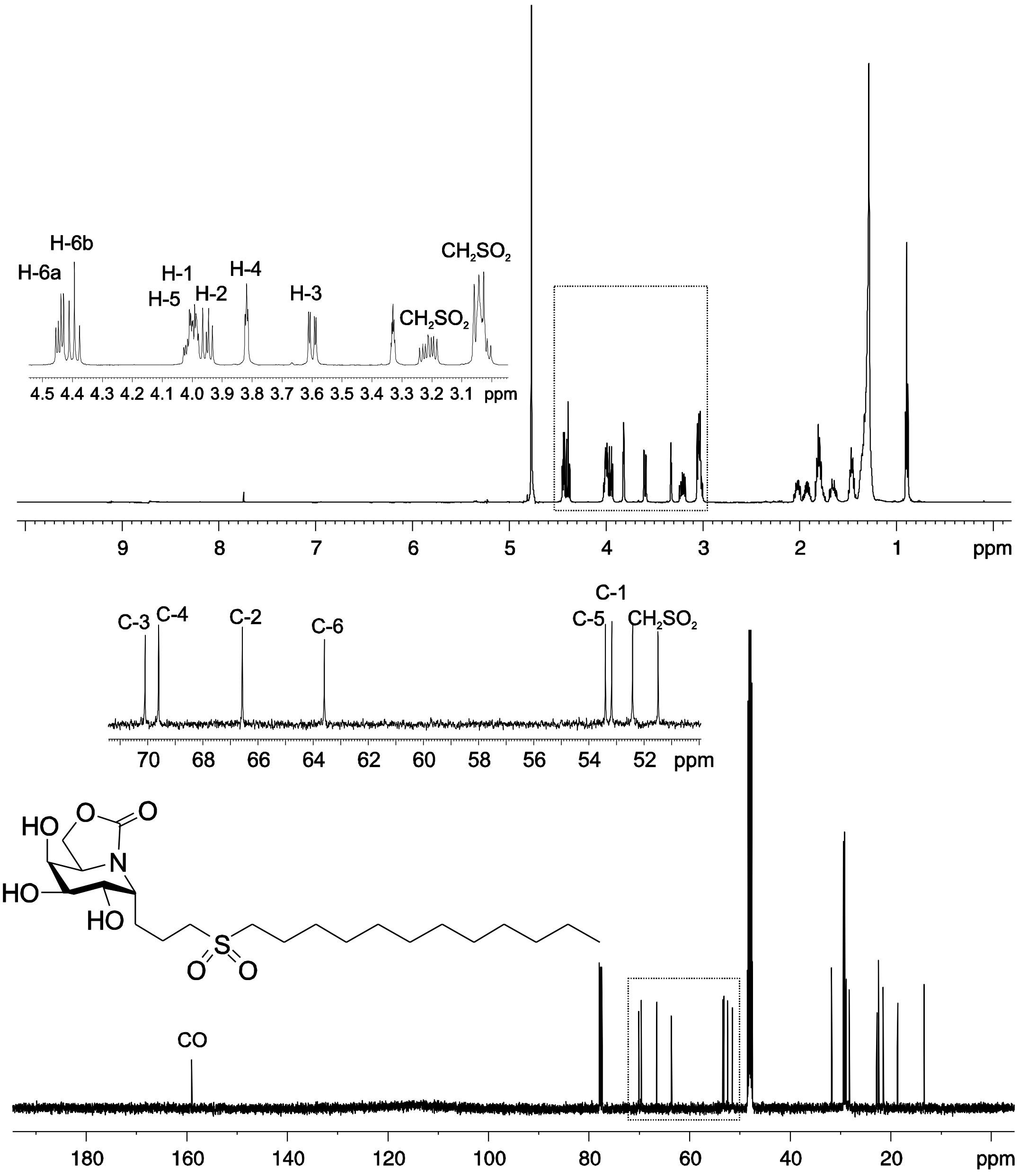

Figure S27. ^{1}H and ^{13}C NMR spectra (500 MHz and 125.7 MHz, 7:3 CD_3OD-$CDCl_3$) of **11**.

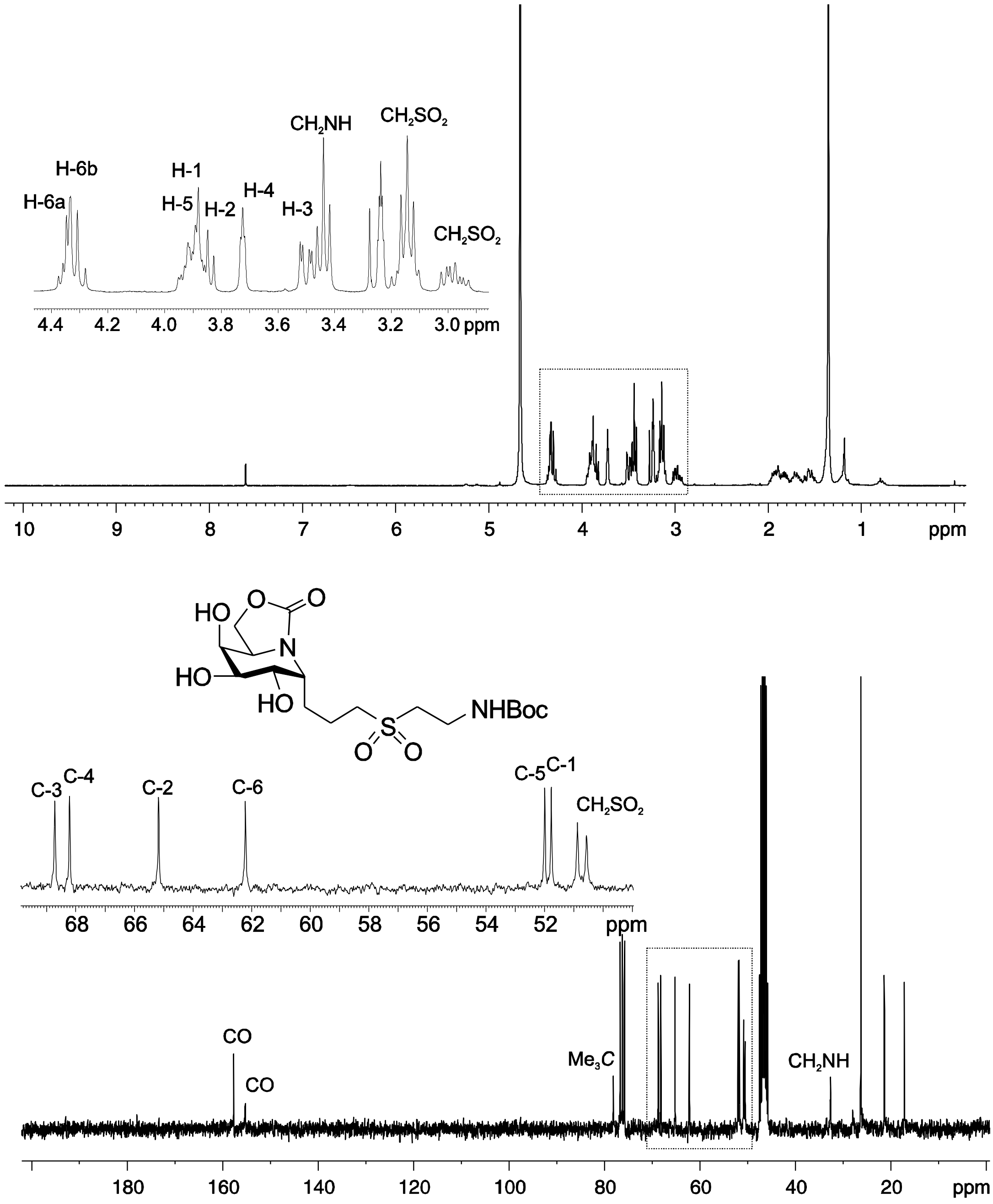

Figure S28. ^{1}H and ^{13}C NMR spectra (300 MHz and 75.5 MHz, 7:3 CD_3OD-$CDCl_3$) of **12**.

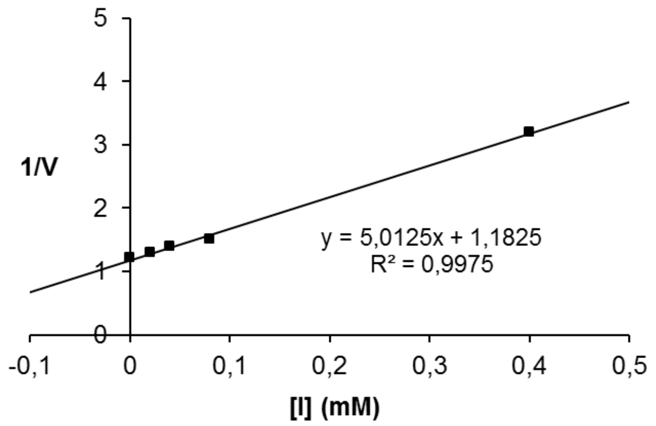

Figure S29. Dixon Plot for K_i determination (79 μM) of **15** against yeast maltase α-glucosidase.

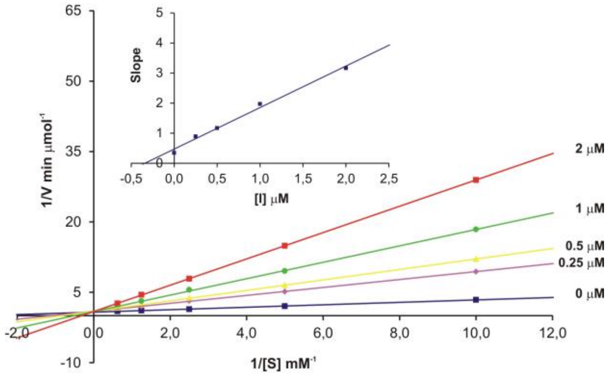

Figure S30. Lineweaver-Burk Plot for K_i determination (0.34 μM) of **1** against yeast maltase α-glucosidase.

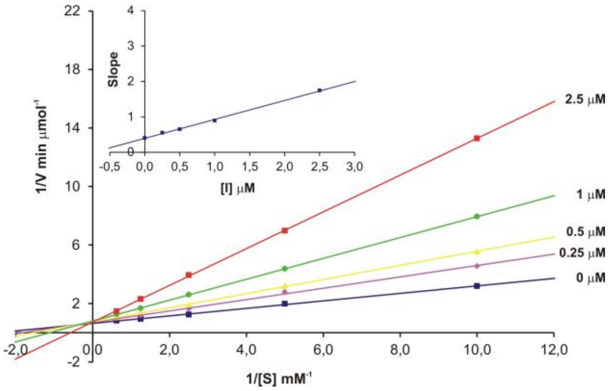

Figure S31. Lineweaver-Burk Plot for K_i determination (0.74 μM) of **2** against yeast maltase α-glucosidase.

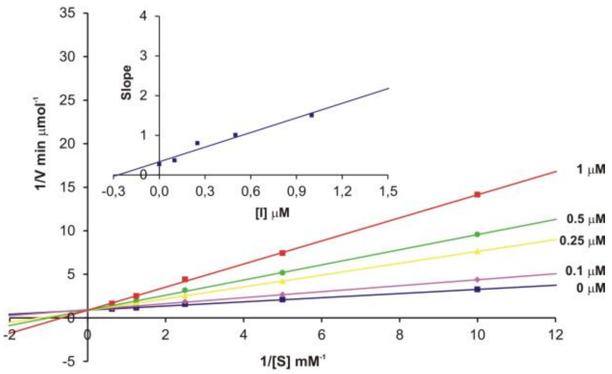

Figure S32. Lineweaver-Burk Plot for K_i determination (0.28 µM) of **3** against yeast maltase α-glucosidase.

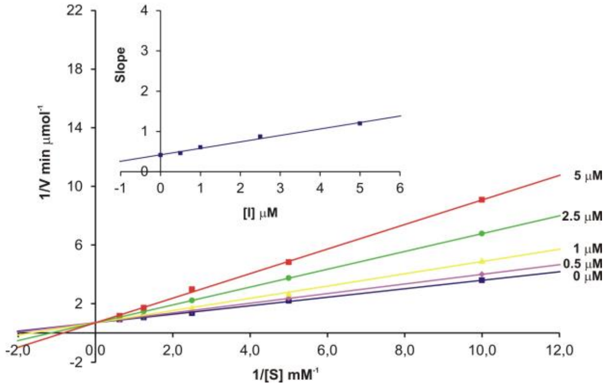

Figure S33. Lineweaver-Burk Plot for K_i determination (2.6 µM) of **4** against yeast maltase α-glucosidase.

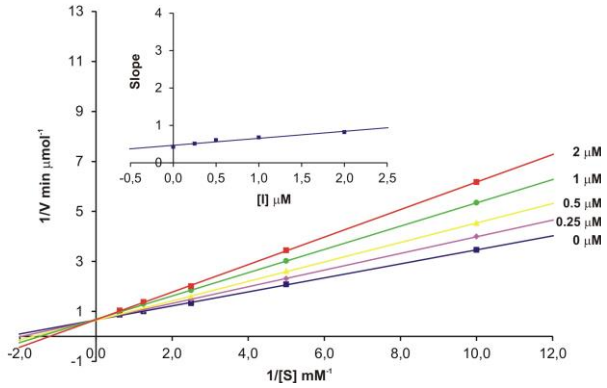

Figure S34. Lineweaver-Burk Plot for K_i determination (2.5 µM) of **5** against yeast maltase α-glucosidase.

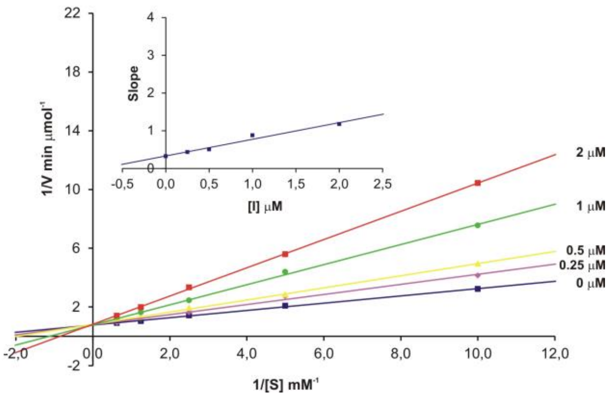

Figure S35. Lineweaver-Burk Plot for K_i determination (0.75 μM) of **6** against yeast maltase α-glucosidase.

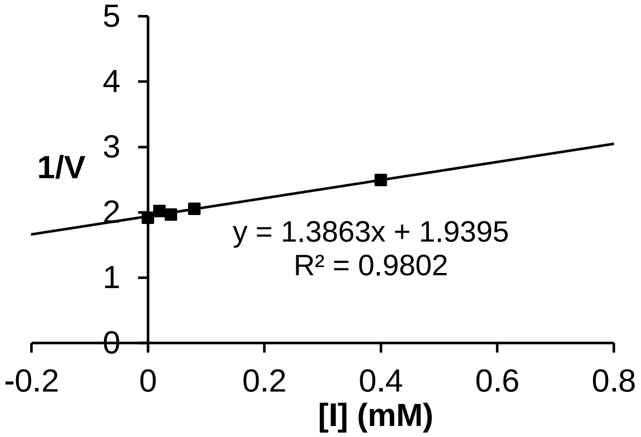

Figure S36. Dixon Plot for K_i determination (400 μM) of **1** against bovine liver β-glucosidase.

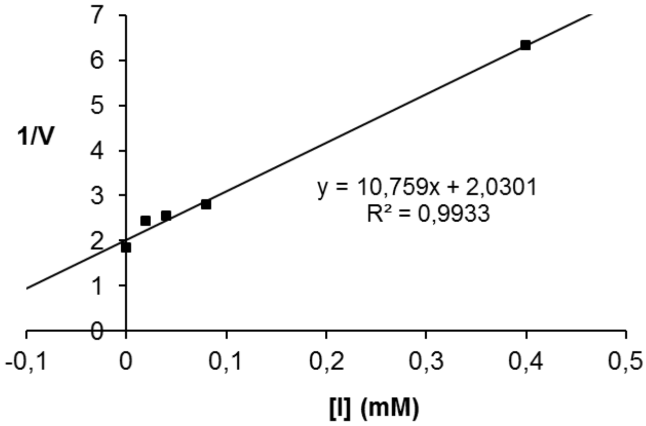

Figure S37. Dixon Plot for K_i determination (54 μM) of **2** against bovine liver β-glucosidase.

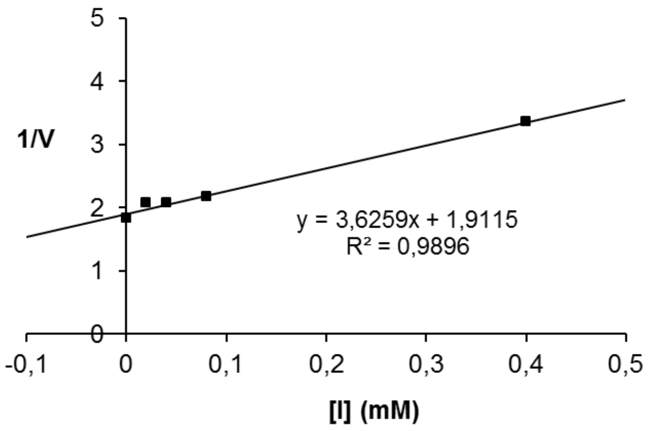

Figure S38. Dixon Plot for K_i determination (151 μM) of **3** against bovine liver β-glucosidase.

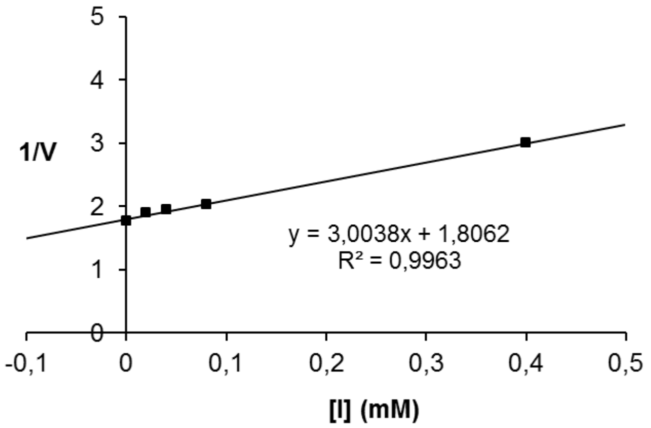

Figure S39. Dixon Plot for K_i determination (172 μM) of **4** against bovine liver β-glucosidase.

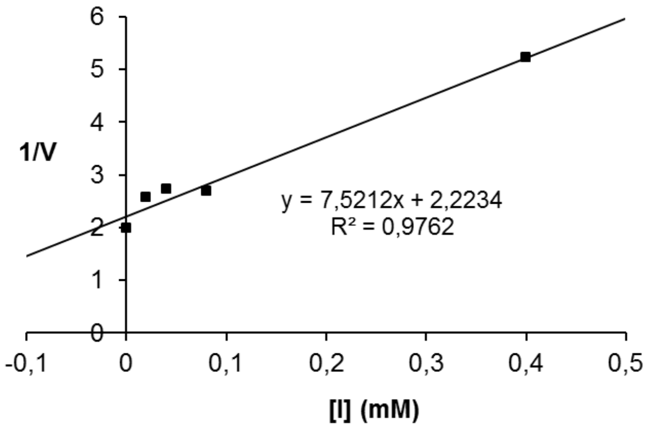

Figure S40. Dixon Plot for K_i determination (85 μM) of **5** against bovine liver β-glucosidase.

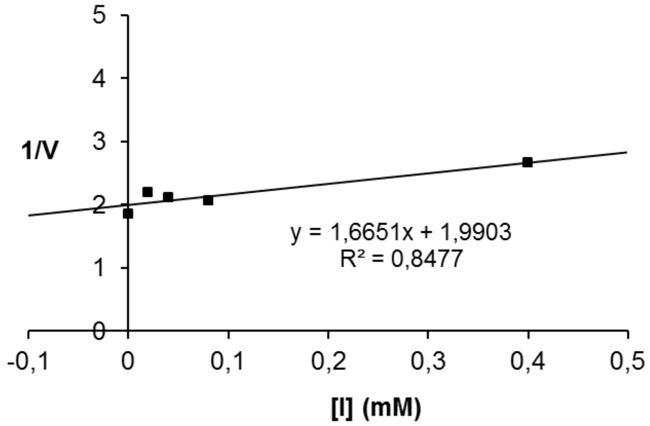

Figure S41. Dixon Plot for K_i determination (342 μM) of **6** against bovine liver β-glucosidase.

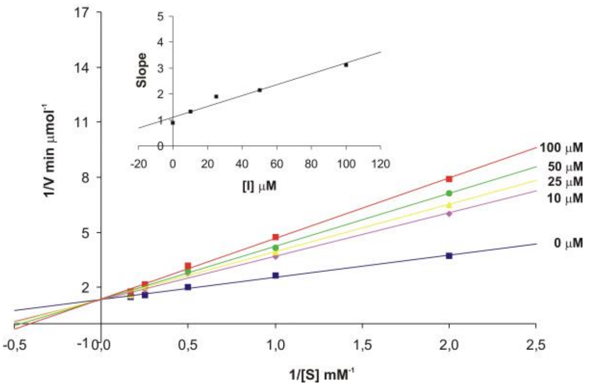

Figure S42. Lineweaver-Burk Plot for K_i determination (53 μM) of **8** against bovine liver β-glucosidase.

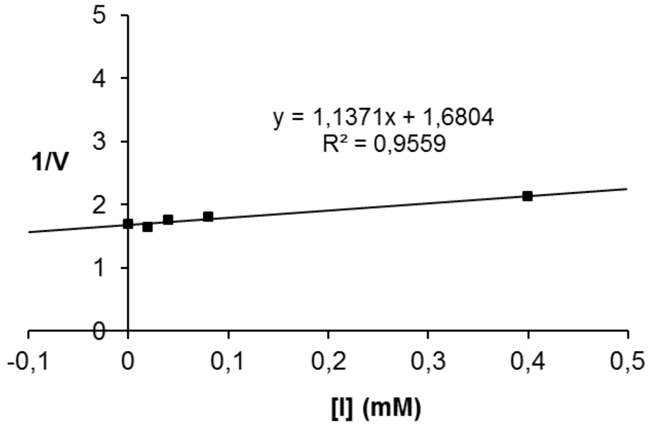

Figure S43. Dixon Plot for K_i determination (422 μM) of **10** against bovine liver β-glucosidase.

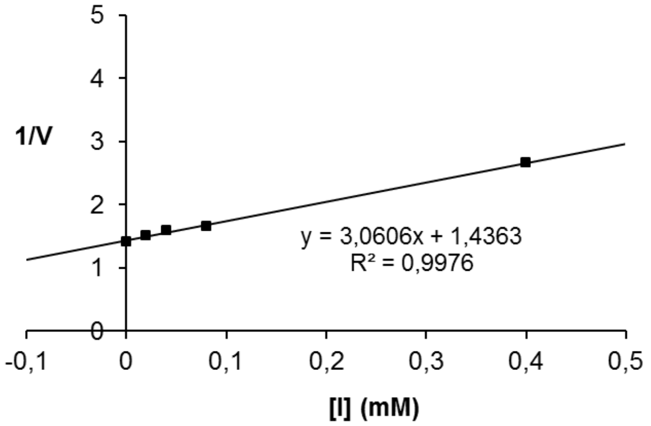

Figure S44. Dixon Plot for K_i determination (134 μM) of **11** against bovine liver β-glucosidase.

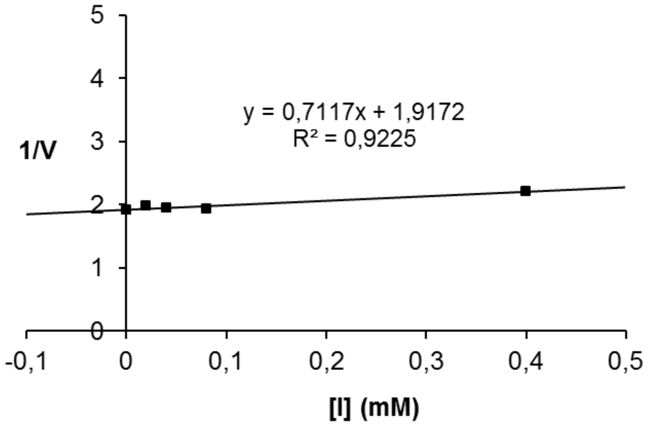

FigureS45. Dixon Plot for K_i determination (770 μM) of **12** against bovine liver β-glucosidase.

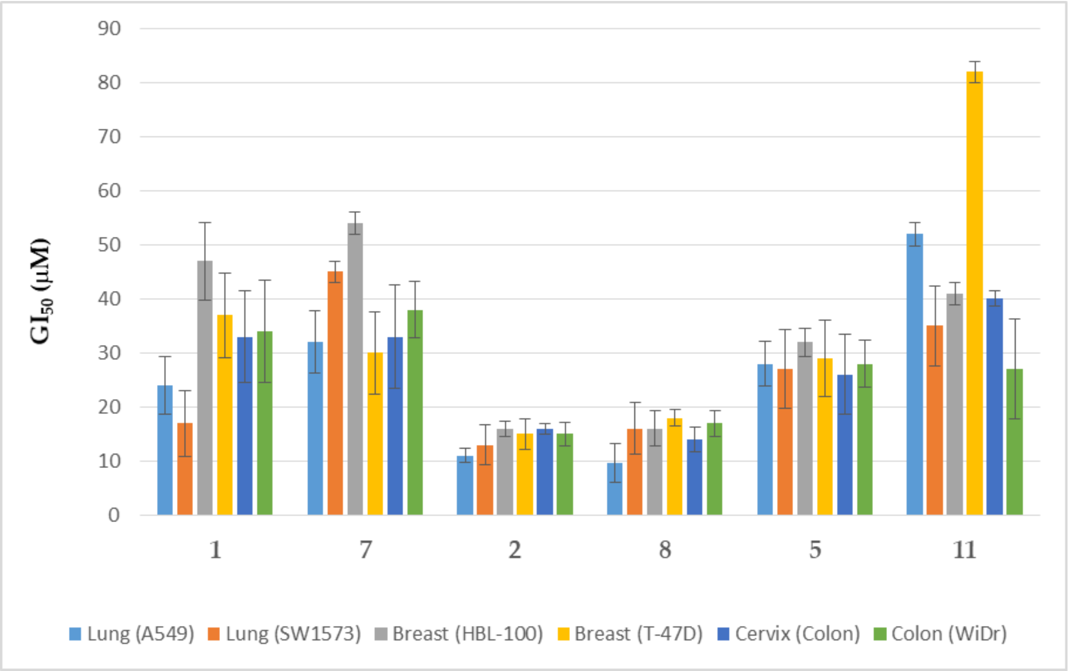

FigureS46. Anti-proliferative activity (GI_{50}) of **1**, **2**, **5**, **7**, **8**, **11** against different human solid tumor cell lines. Compounds **3**, **4**, **6**, **9**, **10** and **12** did not achieved 50% growth inhibition at the highest concentration tested (100 μM).
